# Quantification of intraskeletal histovariability in *Alligator mississippiensis* and implications for vertebrate osteohistology

**DOI:** 10.7717/peerj.422

**Published:** 2014-06-03

**Authors:** Holly N. Woodward, John R. Horner, James O. Farlow

**Affiliations:** 1 Department of Anatomy and Cell Biology, Oklahoma State University Center for Health Sciences, Tulsa, OK, USA; 2 Museum of the Rockies and Department of Earth Sciences, Montana State University, Bozeman, MT, USA; 3 Department of Geosciences, Indiana-Purdue University, Fort Wayne, IN, USA

**Keywords:** Histology, Ontogeny, Alligator, Paleontology, Intraskeletal, Growth rates, Variation

## Abstract

Bone microanalyses of extant vertebrates provide a necessary framework from which to form hypotheses regarding the growth and skeletochronology of extinct taxa. Here, we describe the bone microstructure and quantify the histovariability of appendicular elements and osteoderms from three juvenile American alligators (*Alligator mississippiensis*) to assess growth mark and tissue organization within and amongst individuals, with the intention of validating paleohistological interpretations. Results confirm previous observations that lamellar and parallel fibered tissue organization are typical of crocodylians, and also that crocodylians are capable of forming woven tissue for brief periods. Tissue organization and growth mark count varies across individual skeletal elements and reveal that the femur, tibia, and humerus had the highest annual apposition rates in each individual. Cyclical growth mark count also varies intraskeletally, but data suggest these inconsistencies are due to differing medullary cavity expansion rates. There was no appreciable difference in either diaphyseal circumference or cyclical growth mark circumferences between left and right element pairs from an individual if diaphyses were sampled from roughly the same location. The considerable intraskeletal data obtained here provide validation for long-held paleohistology assumptions, but because medullary expansion, cyclical growth mark formation, and variable intraskeletal growth rates are skeletal features found in tetrapod taxa living or extinct, the validations presented herein should be considered during any tetrapod bone microanalysis.

## Introduction

The study of fossil bone microstructure grows increasingly important for reconstructing extinct vertebrate life histories. To interpret the patterns observed in fossil bone microstructure, transverse diaphyseal sections of extant vertebrates are often assessed as a framework for comparison. However, the extent to which individual skeletal variation affects generalized paleohistological interpretations of a taxon’s growth history is largely unknown.

With that in mind, we examine the bone microstructure of left and right forelimb and hindlimb skeletal elements in three specimens of American alligator (*Alligator mississippiensis*) to achieve the following goals: (1) Address intraskeletal cyclical growth mark (CGM) counts to verify CGM utility in vertebrate paleohistology. Cortical bone tissue often possesses CGMs representing periodically slowed or arrested growth (de Ricqlès, 1980; Frylestam & von Schantz, 1977; Hemelaar & van Gelder, 1980; Hutton, 1986; Peabody, 1961; Tucker, 1997). The presence of CGMs is widespread in taxa requiring more than a single year to reach skeletal maturity, including mammals (e.g., Bourdon et al., 2009; Castanet et al., 2004; de Ricqlès, 1980; de Ricqlès, Padian & Horner, 2001; Köhler et al., 2012; Peabody, 1961; Turvey, Green & Holdaway, 2005). Because CGMs have an annual periodicity in extant vertebrates for which data exist (Castanet et al., 1993), the presence of CGMs is heavily relied upon for determination of annual growth rates and age estimates in extinct taxa. However, previous non-avian dinosaur bone microanalyses reveal that CGM count varies based on the particular limb bone sampled, and even by sampling locality along the diaphysis (Chinsamy, 1993; Horner, de Ricqlès & Padian, 1999; Horner, de Ricqlès & Padian, 2000). Finding such discrepancies in extant vertebrate bone tissue and their underlying causes will help determine the reliability of using CGMs for growth assessment and skeletochronology of not only dinosaurs but vertebrates in general.

(2) Compare differences in bone tissue organization and bone growth rates across different skeletal elements. The general consensus from examining ratites, ducks, and quail is that tissue organization and growth rates vary within the diaphysis of a bone, between diaphyses of different elements from the same individual, and between the diaphyses of homologous elements in different individuals (Castanet et al., 1996; Castanet et al., 2000; Starck & Chinsamy, 2002). Our study on alligators offers a complementary test of growth variability, thereby contributing information on intra- and interskeletal variability applicable to other vertebrate groups.

(3) Test assumptions concerning tissue organization, CGM presence, and CGM circumference. The incompleteness of the fossil record or imposed sampling restrictions often limits researchers to one bone of an element pair from an individual for histological examination. Thus, tissue organization, CGM count, CGM spacing, and CGM circumferences observed in the cortex of that bone are assumed equivalent in the contralateral element. Because such basic assumptions have far-reaching consequences for interpreting bone tissue microstructures in both extant and extinct vertebrates, they should be implicitly tested and confirmed. Our intraskeletal, paired element osteoanalysis of an extant taxon provides validation for paleohistology foundations and helps ensure that conclusions regarding the growth histories of any extinct vertebrate taxon are based on testable histological observations rather than long-held assumptions.

## Materials & Methods

### Collection

Paired limbs (excluding manus and pes), scapula, and coracoid, as well as the largest two nuchal (i.e., postoccipital) osteoderms were obtained from three immature male American alligators (*Alligator mississippiensis*). In February 2007, biological staff at the state-owned Rockefeller Wildlife Refuge (RWR) in Grand Chenier, Louisiana, USA, terminated three alligators from the refuge for use in research unassociated with our project or labs. Upon the request of the senior author, the biological staff salvaged limbs and nuchal osteoderms from the deceased alligators and supplied them for this study. Two individuals, MOR-OST 1649 (30.8 cm total length) and MOR-OST 1650 (141 cm total length), were raised in captivity at RWR, while MOR-OST 1648 (95.3 cm total length) was wild-caught on the refuge. Unfortunately, no snout-vent length, age at death, or body mass data were available, but the use of immature individuals minimizes the number of CGMs lost to cortical remodeling and medullary expansion so that accurate age determination is possible. Previously prepared diaphyseal thin sections from a hatchling alligator (MOR-OST 1647) were also included in this study to provide neonate cortical dimensions.

### Sampling methods

**Figure 1:**
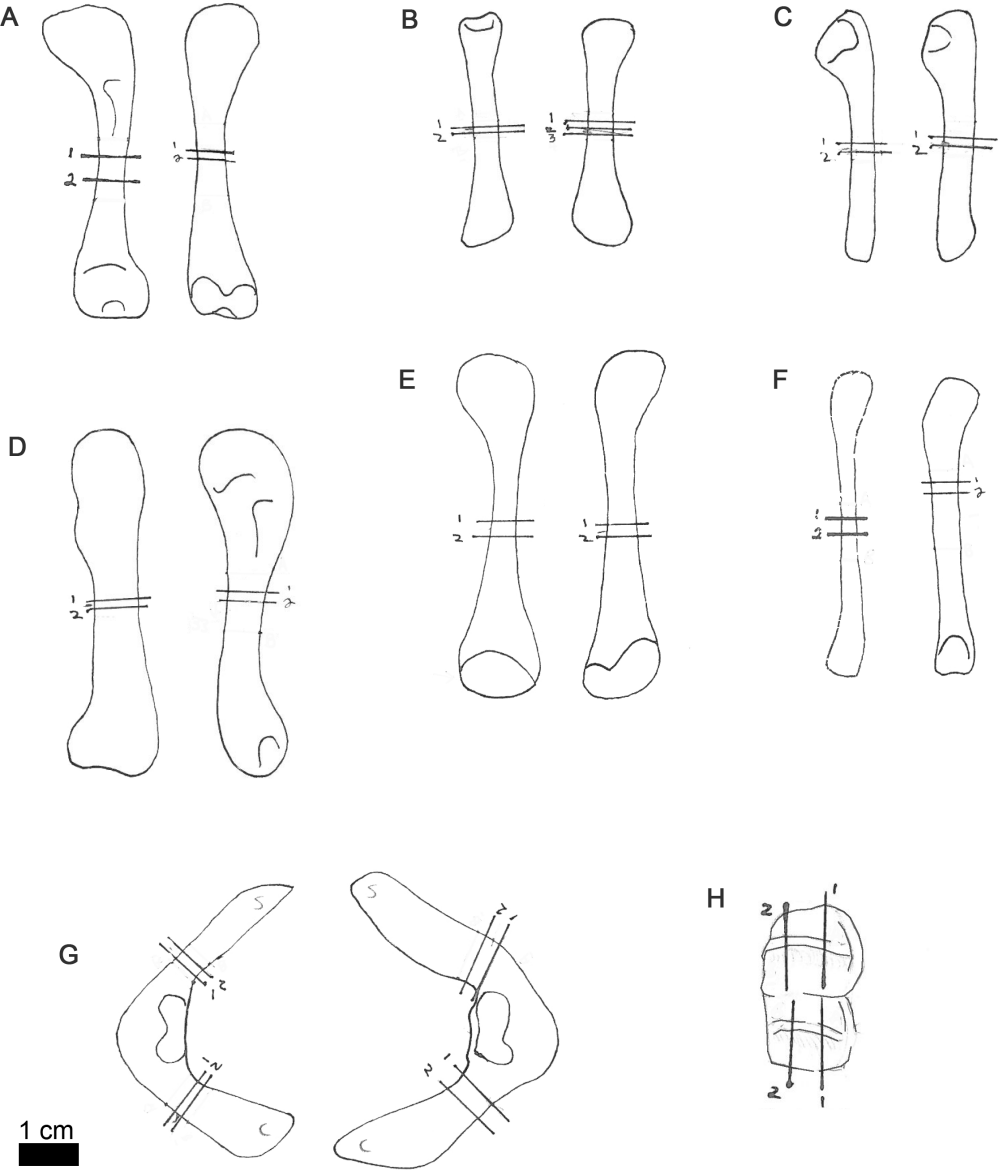
Line drawings of MOR-OST 1648 skeletal elements with approximate sampling locations indicated. Long bone elements are arranged left and right in pairs, with proximal ends towards the top of each sub-figure for humerus (A), radius (B), ulna (C), femur (D), tibia (E), and fibula (F). Left and right scapulocoracoids (G) are drawn in posterolateral view, the scapula indicated with “s”, and coracoid with “c”. Two nuchal osteoderms (H) were drawn with anterior to the right. Straight lines through the drawings indicate the approximate location for sampling, while the numbers correspond to the number and order of samples removed for processing.

**Figure 2:**
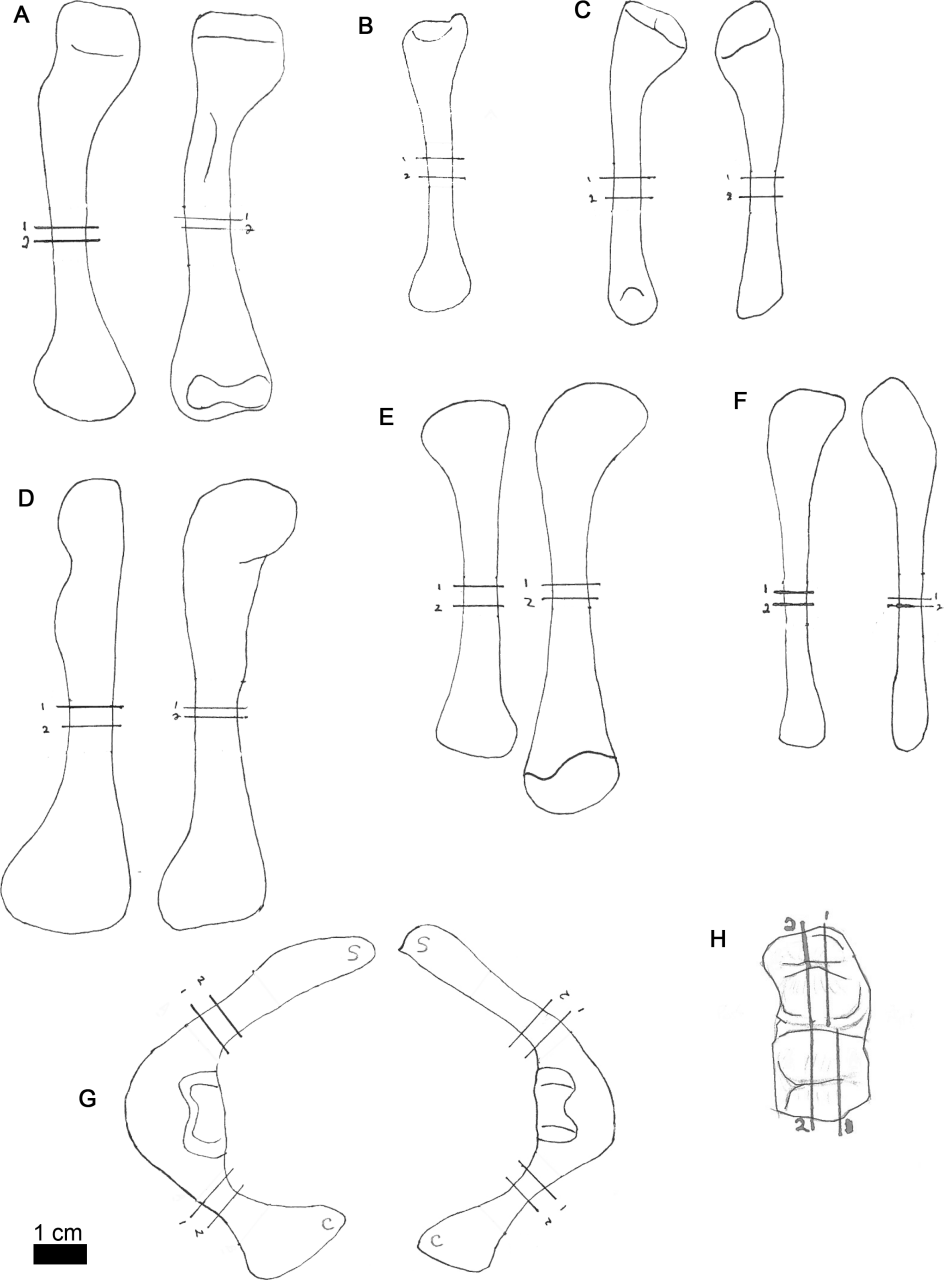
Line drawings of MOR-OST 1649 skeletal elements with approximate sampling locations indicated. Long bone elements are arranged left and right in pairs, with proximal ends towards the top of each sub-figure for humerus (A), radius ((B); only the left radius was sampled), ulna (C), femur (D), tibia (E), and fibula (F). Left and right scapulocoracoids (G) are drawn in posterolateral view, the scapula indicated with “s”, and coracoid with “c”. Two nuchal osteoderms (H) were drawn with anterior to the right. Straight lines through the drawings indicate the approximate location for sampling, while the numbers correspond to the number and order of samples removed for processing.

**Figure 3:**
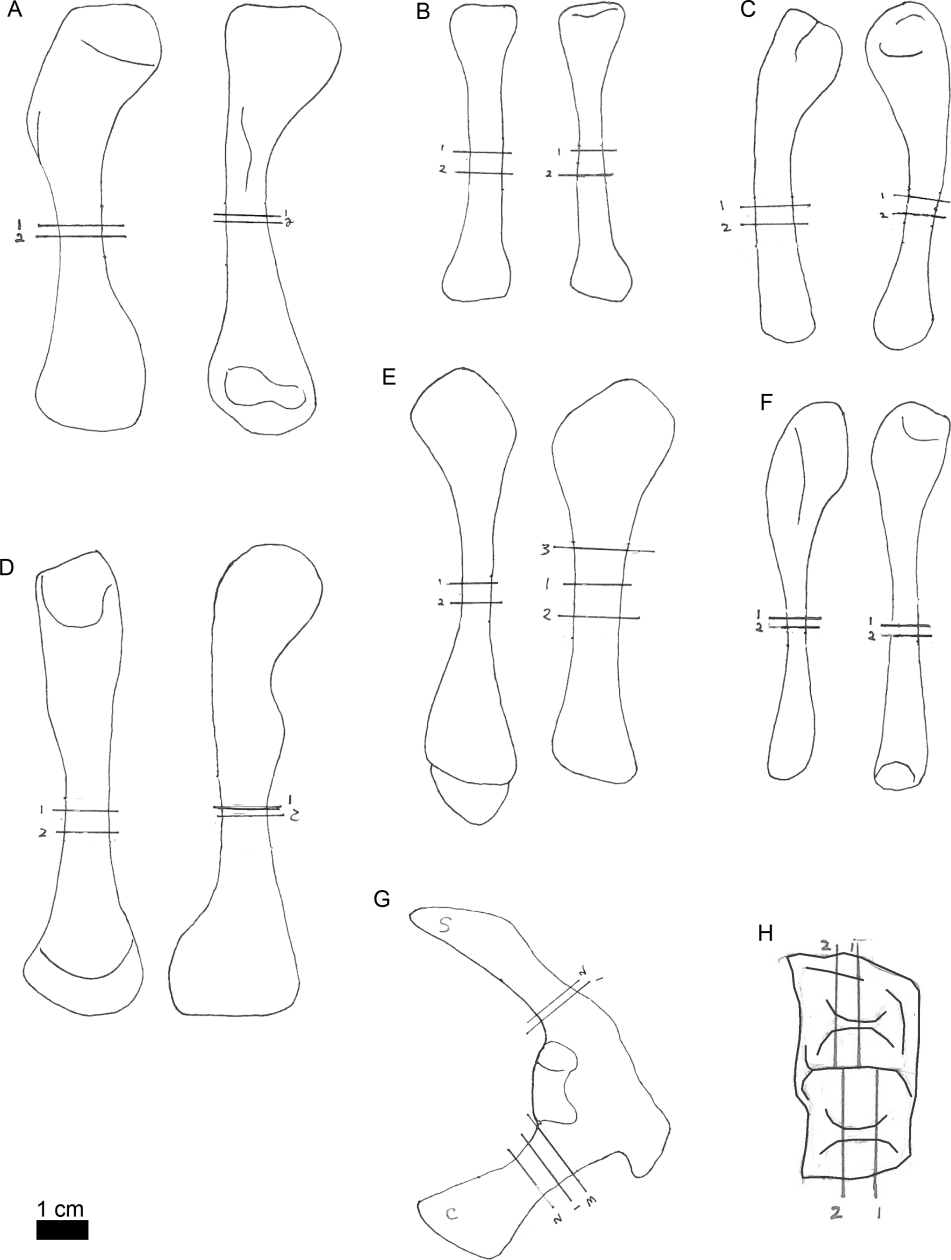
Line drawings of MOR-OST 1650 skeletal elements with approximate sampling locations indicated. Long bone elements are arranged left and right in pairs, with proximal ends towards the top of each sub-figure for humerus (A), radius ((B); only the left radius was sampled), ulna (C), femur (D), tibia (E), and fibula (F). The right scapulocoracoid (G) is drawn in posterolateral view, the scapula indicated with “s”, and coracoid with “c”. Two nuchal osteoderms (H) were drawn with anterior to the right. Straight lines through the drawings indicate the approximate location for sampling, while the numbers correspond to the number and order of samples removed for processing.

The specimens were prepared for osteohistology and examined at the Museum of the Rockies (MOR) in Bozeman, Montana, USA. Serial transverse sections were removed from two nuchal osteoderms as well as from the minimum diaphyseal circumference of long bones where cortex is thickest (Sander & Andrássy, 2006), and from the most circumferentially restricted region of scapula and coracoid blades (Figs. 1–3). Both left and right bones of each element were sampled, except the scapula and coracoid of MOR-OST 1650 and the radius of MOR-OST 1649. Only a single specimen was available for examination in those cases: the right scapulocoracoid of MOR-OST 1650 and the left radius of MOR-OST 1649. Thin sections were prepared using published techniques for extant mineralized (=undecalcified) crocodylian bone (Schweitzer et al., 2007), except that the bone samples removed were not cleared in xylene after undergoing the ethanol dehydration series prior to embedding. At least two slides were made from each diaphysis and polished until thin enough to observe bone fiber orientation, osteocyte lacunae, and CGMs using a petrographic transmitted light microscope. This was most often achieved at specimen thicknesses between 30 µm and 40 µm. One thin section from each diaphysis was etched in 1% formic acid for thirty seconds with agitation and rinsed with water. The etched slides were then immersed for five minutes in a warm (57 °C) Toluidine blue staining solution consisting of 1% stock solution of Toluidine blue (Aldrich Chemical Company, St. Louis, MO) and a pH = 8.0 phosphate buffer (0.075 mL toluidine blue : 1 mL phosphate buffer). Toluidine blue is often used to stain bone and cartilage because it reacts with proteoglycans and other proteins in basic solutions. In other skeletochronological studies, it has been shown to differentiate CGMs more clearly compared to other stains, such as hematoxylin and eosin (Waye & Gregory, 1998). Finally, thin section slides were cover-slipped using Poly-Mount (Polysciences, Inc., Warrington, PA) medium.

### Analysis

Every thin section was examined in polarized light with either 4 X or 10 X objectives using an Optiphot-Pol (Nikon Instruments Inc., Tokyo, Japan) polarizing microscope and either a circular polarizer, a ¼ lambda plate, or a full lambda (530 nm) plate. Images were obtained with a DS-Fi1 digital sight camera (Nikon Instruments Inc., Tokyo, Japan), and compiled using NIS-Elements BR 3.0 (Nikon Instruments Inc., Tokyo, Japan) software. Polarized light rather than bright field was chosen for analysis as the former tended to make fiber orientation and CGMs more evident. Mineralized bone fiber orientation was determined by using polarized light with a full lambda plate, as well as by using circularly polarized light for further confirmation. High resolution images of complete transverse sections for each thin section are digitally reposited online at MorphoBank (O’Leary & Kaufman, 2012), project P731. See Table S1 for a list of slides and accession numbers. Large-file, full resolution images of complete transverse sections can be obtained from the senior author.

**Table 1:** Data collected for the three alligator individuals included in this study. An asterisk appears next to measurements in which the outermost cyclical growth mark (CGM) was omitted from measurements. MOR, Museum of the Rockies, Montana, USA.

Specimen number	Element	Location	Left cortical area (mm^2^)	Right cortical area (mm^2^)	Left circumference (mm^2^)	Right circumference (mm^2^)	Average cortical thickness of left element (mm)
**MOR-OST** **1648**	Coracoid	Medullary cavity	4.79	4.45	14.95	9.90	–
		Hatchling	1.24	1.24	4.81	4.81	–
		CGM 1	Not observed	Not observed	Not observed	Not observed	–
		CGM 2	8.75	8.06	13.41	12.20	–
		CGM 3	10.69	10.58	13.62	13.60	–
		Surface	13.93	13.09	15.63	15.08	–
	Fibula	Medullary cavity	0.06	0.72	2.99	3.62	–
		Hatchling	0.43	0.43	3.55	3.55	0.37
		CGM 1	Not fully traceable	Not observed	Not fully traceable	Not observed	–
		CGM 2	Not fully traceable	Not observed	Not fully traceable	Not observed	1.05
		CGM 3	4.24	4.29	7.72	7.85	1.17
		Surface	5.27	5.40	8.63	8.81	1.30*
	Femur	Medullary cavity	2.55	3.40	6.08	7.01	–
		Hatchling	1.61	1.61	6.74	6.74	0.71
		CGM 1	Not fully traceable	Not fully traceable	Not fully traceable	Not fully traceable	–
		CGM 2	15.12	15.12	14.82	14.77	2.20
		CGM 3	18.52	18.34	16.45	16.29	2.43
		Surface	24.70	24.42	18.97	18.81	2.81
	Humerus	Medullary cavity	2.06	1.91	5.41	5.22	–
		Hatchling	1.02	1.02	4.06	4.06	0.57
		CGM 1	Not fully traceable	Not fully traceable	Not fully traceable	Not fully traceable	–
		CGM 2	Not fully traceable	Not fully traceable	Not fully traceable	Not fully traceable	–
		CGM 3	12.90	12.94	13.47	13.51	2.01
		Surface	17.31	17.67	15.61	15.76	2.35
	Radius	Medullary cavity	0.71	0.67	3.17	3.10	–
		Hatchling	0.42	0.42	3.42	3.42	0.37
		CGM 1	1.21	1.12	4.13	4.00	0.61
		CGM 2	3.21	3.16	6.76	6.82	0.99
		CGM 3	4.22	4.14	7.78	7.79	1.14
		Surface	5.47	5.45	8.86	8.98	1.30
	Scapula	Medullary cavity	1.85	4.08	8.70	9.46	–
		Hatchling	0.70	0.70	5.73	5.73	–
		CGM 1	Not fully traceable	Not fully traceable	Not fully traceable	Not fully traceable	–
		CGM 2	5.56	Not fully traceable	12.17	Not fully traceable	–
		CGM 3	7.40	Not fully traceable	13.70	Not fully traceable	–
		Surface	10.04	16.33	15.33	16.55	–
	Tibia	Medullary cavity	2.41	2.07	5.89	5.54	–
		Hatchling	0.96	0.96	3.95	3.95	0.55
		CGM 1	Not fully traceable	Not fully traceable	Not fully traceable	Not fully traceable	–
		CGM 2	10.10	9.92	11.99	11.90	1.76
		CGM 3	12.45	12.32	13.32	13.20	1.96
		Surface	16.13	16.08	15.11	15.10	2.24
	Ulna	Medullary cavity	0.85	0.99	3.63	3.96	–
		Hatchling	0.39	0.39	2.61	2.61	0.36
		CGM 1	Not fully traceable	Not fully traceable	Not fully traceable	Not fully traceable	–
		CGM 2	3.88	3.91	7.62	7.61	1.12
		CGM 3	5.28	5.35	8.89	8.91	1.31
		Surface	7.22	7.33	10.41	10.49	1.52
**MOR-OST** **1649**	Coracoid	Medullary cavity	5.41	5.42	15.61	16.67	–
		Hatchling	1.24	1.24	4.81	4.81	–
		CGM 1	5.29	Not fully traceable	11.27	Not fully traceable	–
		CGM 2	9.52	9.91	13.37	13.35	–
		CGM 3	15.08	15.85	16.07	16.18	–
		CGM 4	18.38	19.52	17.58	17.94	–
		CGM 5	Not fully traceable	Not observed	Not fully traceable	Not observed	–
		Surface	22.40	23.94	19.26	19.73	–
	Fibula	Medullary cavity	0.68	0.74	3.12	3.28	–
		Hatchling	0.43	0.43	3.55	3.55	0.37
		CGM 1	Not fully traceable	Not fully traceable	Not fully traceable	Not fully traceable	–
		CGM 2	3.51	3.84	7.03	7.37	1.04
		CGM 3	5.60	5.67	8.91	8.96	1.33
		CGM 4	8.21	8.16	10.83	10.81	1.61
		CGM 5	Not fully traceable	Not fully traceable	Not fully traceable	Not fully traceable	–
		Surface	11.06	11.02	12.57	12.63	1.86*
	Femur	Medullary cavity	6.47	5.03	12.05	11.43	–
		Hatchling	1.61	1.61	6.74	6.74	0.71
		CGM 1	Not observed	Not observed	Not observed	Not observed	–
		CGM 2	11.58	13.68	12.87	14.06	1.93
		CGM 3	22.54	24.65	17.99	18.78	2.71
		CGM 4	35.20	36.23	22.56	22.75	3.39
		CGM 5	Not fully traceable	Not observed	Not fully traceable	Not observed	–
		Surface	47.93	48.57	26.22	26.34	3.89*
	Humerus	Medullary cavity	3.47	3.01	7.35	6.92	–
		Hatchling	1.02	1.02	4.06	4.06	0.57
		CGM 1	Not fully traceable	Not fully traceable	Not fully traceable	Not fully traceable	–
		CGM 2	7.48	7.13	10.31	10.07	1.53
		CGM 3	14.44	14.42	14.29	14.26	2.14
		CGM 4	23.12	23.44	18.09	18.26	2.70
		CGM 5	31.25	Not observed	21.01	Not observed	3.14
		Surface	32.53	32.86	21.44	21.55	3.2*
	Radius	Medullary cavity	0.51	Not measured	2.82	Not measured	–
		Hatchling	0.42	Not measured	3.42	Not measured	0.37
		CGM 1	3.07	Not measured	6.67	Not measured	0.96
		CGM 2	4.95	Not measured	8.48	Not measured	1.26
		CGM 3	7.22	Not measured	10.20	Not measured	1.52
		CGM 4	10.12	Not measured	12.02	Not measured	1.78
		CGM 5	Not observed	Not measured	Not observed	Not measured	–
		Surface	10.91	Not measured	12.49	Not measured	1.84
	Scapula	Medullary cavity	2.26	5.73	12.03	16.12	–
		Hatchling	0.70	0.70	5.73	5.73	–
		CGM 1	Not fully traceable	Not fully traceable	Not fully traceable	Not fully traceable	–
		CGM 2	4.30	Not fully traceable	14.01	Not fully traceable	–
		CGM 3	8.35	Not fully traceable	16.79	Not fully traceable	–
		CGM 4	13.49	14.89	19.58	17.88	–
		CGM 5	Not fully traceable	Not fully traceable	Not fully traceable	Not fully traceable	–
		Surface	20.51	22.31	22.10	21.05	–
	Tibia	Medullary cavity	3.90	4.58	8.39	8.85	–
		Hatchling	0.96	0.96	3.95	3.95	0.55
		CGM 1	Not fully traceable	Not fully traceable	Not fully traceable	Not fully traceable	–
		CGM 2	7.98	7.56	10.87	10.47	1.63
		CGM 3	14.78	14.70	14.56	14.47	2.22
		CGM 4	22.28	22.72	17.81	17.98	2.70
		CGM 5	Not fully traceable	Not fully traceable	Not fully traceable	Not fully traceable	–
		Surface	30.24	30.58	20.73	20.86	3.15*
	Ulna	Medullary cavity	1.11	1.11	4.52	4.32	–
		Hatchling	0.39	0.39	2.61	2.61	0.36
		CGM 1	Not observed	Not observed	Not observed	Not observed	–
		CGM 2	Not observed	Not observed	Not observed	Not observed	–
		CGM 3	6.26	6.06	9.74	9.53	1.43
		CGM 4	9.45	9.30	11.91	11.90	1.76
		CGM 5	12.90	12.91	13.94	14.06	2.05
		Surface	14.05	14.05	14.51	14.59	2.14*
**MOR-OST** **1650**	Coracoid	Medullary cavity	Not measured	6.90	Not measured	16.27	–
		Hatchling	Not measured	1.24	Not measured	4.81	–
		CGM 1	Not measured	Not fully traceable	Not measured	Not fully traceable	–
		CGM 2	Not measured	6.01	Not measured	11.67	–
		CGM 3	Not measured	15.33	Not measured	15.97	–
		CGM 4	Not measured	17.47	Not measured	16.88	–
		CGM 5	Not measured	Not observed	Not measured	Not observed	–
		Surface	Not measured	28.93	Not measured	21.50	–
	Fibula	Medullary cavity	1.43	1.31	4.67	4.41	–
		Hatchling	0.43	0.43	3.55	3.55	0.37
		CGM 1	3.48	3.87	7.08	7.45	1.04
		CGM 2	5.23	5.50	8.66	8.88	1.29
		CGM 3	7.89	8.00	10.62	10.71	1.60
		CGM 4	9.22	9.43	11.56	11.69	1.73
		CGM 5	Not fully traceable	Not fully traceable	Not fully traceable	Not fully traceable	–
		Surface	14.31	14.27	14.47	14.37	2.17
	Femur	Medullary cavity	5.56	4.47	9.10	8.50	–
		Hatchling	1.61	1.61	6.74	6.74	0.71
		CGM 1	15.18	12.46	15.20	13.60	2.16
		CGM 2	21.45	19.82	17.73	16.86	2.57
		CGM 3	31.63	31.49	21.30	21.21	3.11
		CGM 4	37.36	38.03	23.13	23.26	3.39
		CGM 5	Not observed	Not observed	Not observed	Not observed	–
		Surface	56.97	59.47	28.50	29.11	4.16
	Humerus	Medullary cavity	3.23	2.09	7.33	5.55	–
		Hatchling	1.02	1.02	4.06	4.06	0.57
		CGM 1	6.78	6.78	9.91	9.78	1.46
		CGM 2	12.67	12.48	13.37	13.28	2.02
		CGM 3	21.07	21.27	17.25	17.36	2.61
		CGM 4	25.05	24.68	18.82	18.69	2.83
		CGM 5	Not observed	Not observed	Not observed	Not observed	–
		Surface	41.56	41.02	24.25	24.04	3.65
	Radius	Medullary cavity	0.68	0.42	3.28	2.54	–
		Hatchling	0.42	0.42	3.42	3.42	0.37
		CGM 1	3.26	2.50	6.92	5.97	1.04
		CGM 2	4.72	4.49	8.30	8.03	1.25
		CGM 3	7.22	7.19	10.20	10.16	1.52
		CGM 4	8.68	8.71	11.18	11.17	1.66
		CGM 5	12.28	14.05	13.88	14.26	2.04
		Surface	13.78	15.65	14.10	14.99	2.09*
	Scapula	Medullary cavity	Not measured	1.06	Not measured	11.40	-
		Hatchling	Not measured	0.70	Not measured	5.73	–
		CGM 1	Not measured	5.50	Not measured	16.59	–
		CGM 2	Not measured	7.66	Not measured	17.89	–
		CGM 3	Not measured	12.43	Not measured	21.52	–
		CGM 4	Not measured	15.39	Not measured	22.58	–
		CGM 5	Not measured	Not observed	Not measured	Not observed	–
		Surface	Not measured	26.25	Not measured	26.87	–
	Tibia	Medullary cavity	4.22	3.98	7.83	7.63	–
		Hatchling	0.96	0.96	3.95	3.95	0.55
		CGM 1	7.24	Not fully traceable	10.17	Not fully traceable	1.50
		CGM 2	13.16	13.60	13.71	13.99	2.06
		CGM 3	21.65	22.96	17.70	18.04	2.64
		CGM 4	25.24	27.10	18.94	19.58	2.85
		CGM 5	Not observed	Not observed	Not observed	Not observed	–
		Surface	40.24	43.56	23.93	24.87	3.57
	Ulna	Medullary cavity	2.47	2.00	6.44	5.71	–
		Hatchling	0.39	0.39	2.61	2.61	0.36
		CGM 1	Not observed	Not observed	Not observed	Not observed	–
		CGM 2	6.05	5.73	9.60	9.38	1.35
		CGM 3	9.50	9.05	11.83	11.62	1.74
		CGM 4	11.37	10.82	12.96	12.66	1.87
		CGM 5	18.53	17.57	16.46	16.04	2.45
		Surface	19.27	18.58	16.74	16.57	2.5*

Trends in osteocyte lacunae appearance were noted, but determination of fiber organization patterns was not dependent upon them. Fiber organization terminology is based on Francillon-Vieillot et al. (1990) and is explicitly defined here. Lamellar fibers are highly organized in parallel within the transverse plane of section, resulting in a plywood pattern of high birefringence and anisotropy and osteocyte lacunae within the lamellar tissue are sparse and flattened. Parallel fibered tissue is similar in anisotropic appearance to lamellar tissue, but with no plywood effect and a more fibrous appearance to the tissue. We apply the term “loosely parallel fibered tissue” when there is an obvious parallel arrangement to the fibers in the transverse plane of section, but when the organization of fibers is somewhat more isotropic than parallel fibered tissue as defined by Francillon-Vieillot et al. (1990). Within both kinds of parallel fibered tissue, the long axes of most osteocyte lacunae are also arranged in parallel. Finally, using the terminology of Francillon-Vieillot et al. (1990), woven tissue is largely isotropic, and no overall arrangement of fiber orientation is detected (but see Stein & Prondvai (2014) for detailed discussion of woven bone and fibrolamellar arrangement). Osteocyte lacunae density is high, and lacunae are scattered at random.

**Figure 4:**
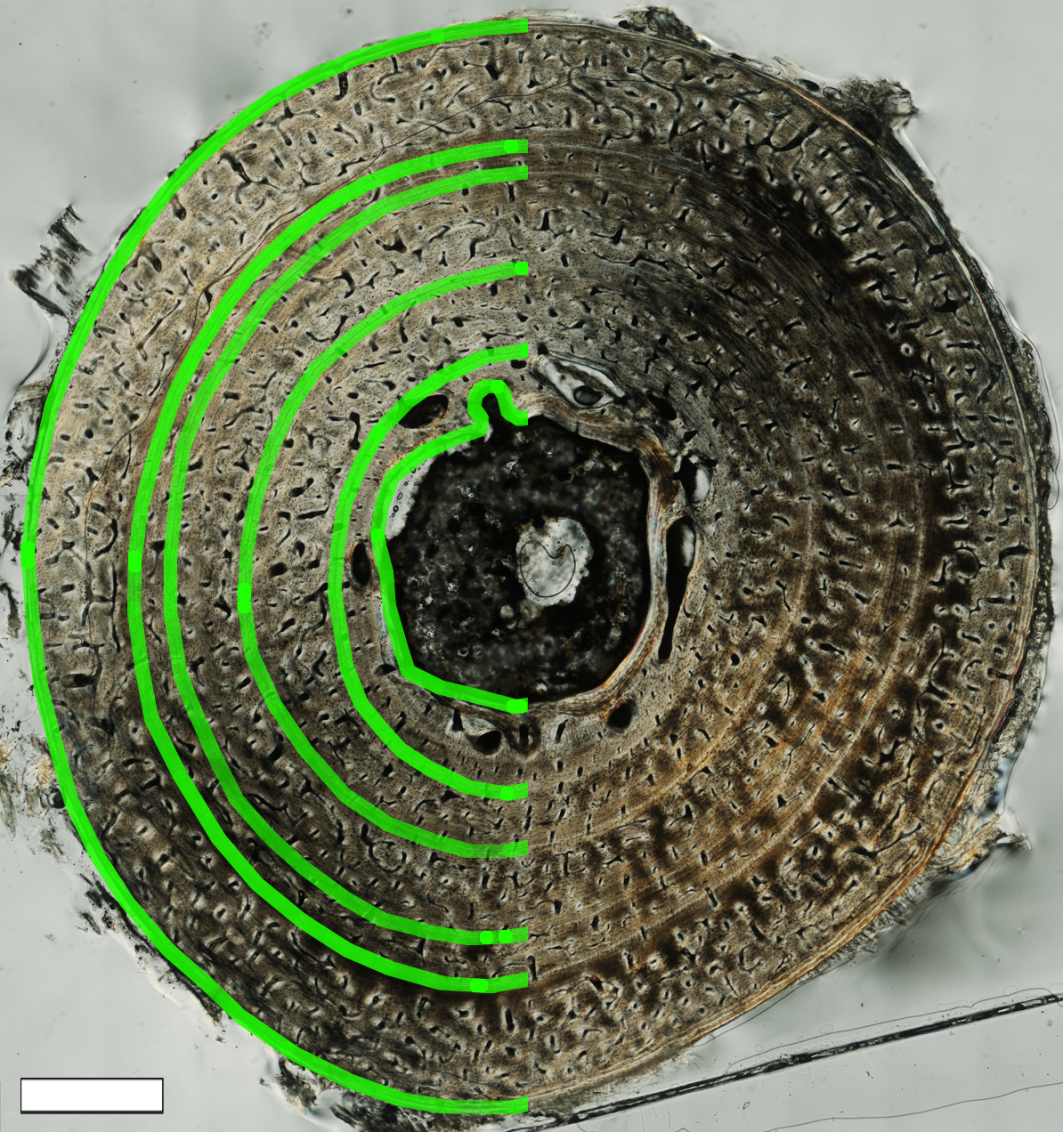
Transverse thin section of MOR-OST 1650 left humerus illustrating the histological features that were digitally traced, including medullary cavity circumference, CGM circumferences, and periosteal surface circumference. On the right side of the image, CGMs in the form of annuli are easily observed as thin white lines. The left side of the image shows each CGM, as well as the medullary cavity boundary and the periosteal surface partially traced in green. The green tracings are exaggerated for clarity, and actual tracings were done using 5 pixel-wide lines. Scale bar, 1 mm.

For each transverse section, the diaphyseal circumference as well as the circumference of each CGM was traced (Fig. 4) in Adobe Photoshop CS3 (Adobe Systems Inc.). CGMs were identified as either lines of arrested growth (LAGs) or annuli. A LAG appears as a thin line and indicates an area of hypermineralized tissue formed due to a temporary cessation of growth (Castanet et al., 1993). An abrupt decrease rather than a pause in apposition is reflected by an often white or translucent band of well-organized parallel fibers (Castanet et al., 1993), containing flattened osteocytes and little to no vascularization. This band of tissue is termed an annulus (Francillon-Vieillot et al., 1990). It is also possible to observe one or more annuli immediately followed by a LAG, indicating a period of slowed growth before eventual growth cessation. CGMs were counted and numbered beginning first with the mark nearest the medullary cavity and proceeding outward towards the bone surface. There were no instances of “double LAGs” (Caetano & Castanet, 1993; Castanet et al., 1993) or LAGs that split or merged. However, regional color variation (i.e., “bands” of darker tissue) about the cortex sometimes imitated CGMs, especially in lamellar tissue. But in such instances the bands were thicker than annuli or LAGs and would fade and blend into the surrounding cortex if traced far enough. And despite the typical layered appearance of lamellar tissue that often makes CGM identification difficult, annuli were recognized as white or translucent rings, and LAGs by thin black lines. This distinction was made more obvious when observing the samples stained with Toulidine blue because the dense lamellar cortical bone stained blue or purple, annuli remained white or translucent, and LAGs would stain very dark purple. In some cases where the cortex was largely lamellar, several thin, faint annuli were found within a zone leading up to a thicker and better defined annulus, and the pattern repeated in following zones.

**Figure 5:**
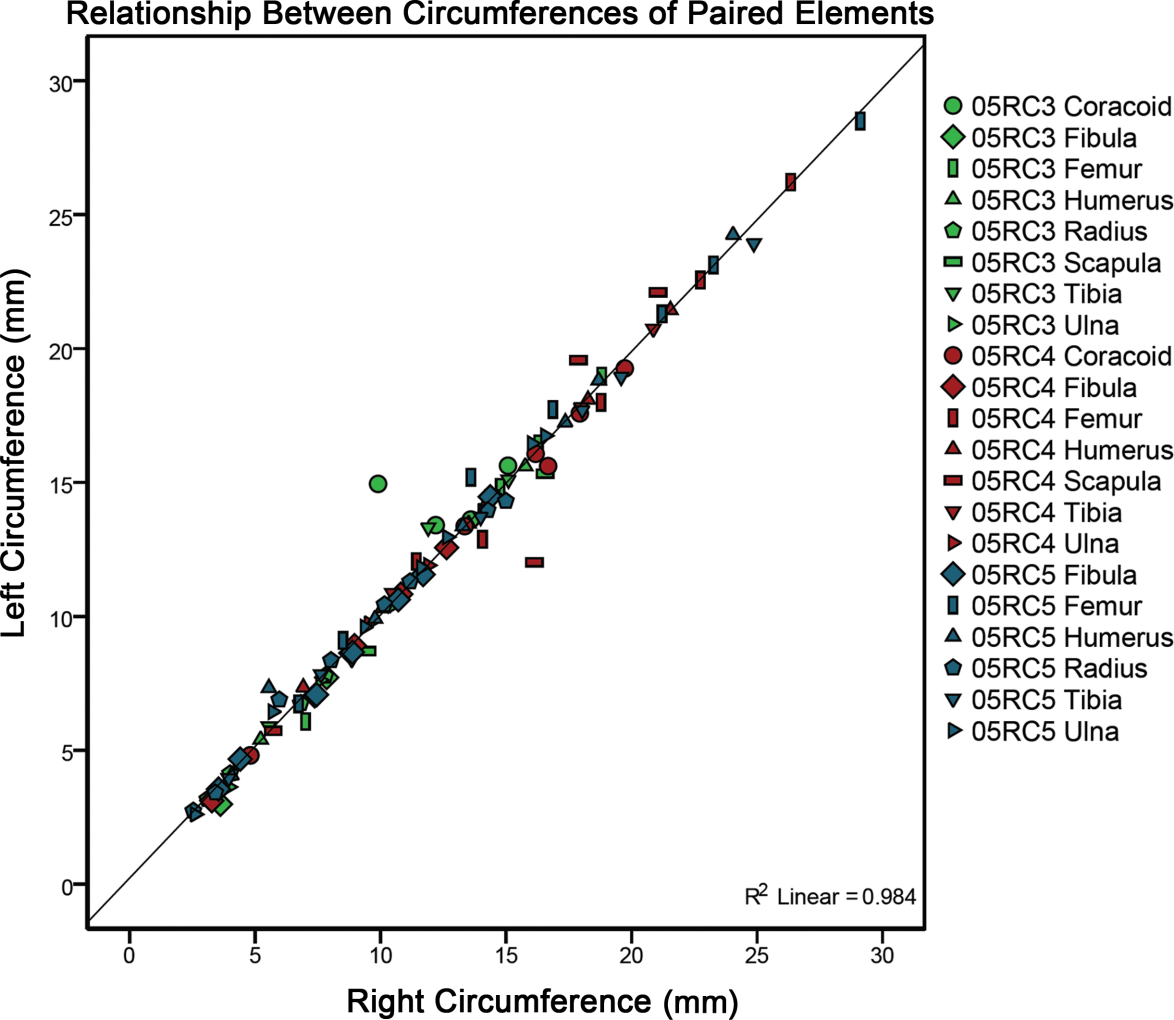
A plot comparing left versus right cyclical growth mark (CGM) and surface diaphyseal circumferences of element pairs. The fitted linear regression with an *R*^2^ value of 0.984 and a slope of 1 demonstrates no significant difference between left and right growth mark circumferences or bone surface circumferences between left and right element pairs from a single individual. Two outliers (a scapula and a coracoid) may be the result of dissimilar sampling locations between left and right elements (see Materials and Methods).

LAGs and annuli were often easily completely traced about the transverse section of the cortex. If the structures in the cortex fit the criteria of CGMs discussed above but were truncated by resorption due to medullary expansion or drift, or became difficult to completely trace due to the mark becoming faint, the CGM was still counted and included in the total. In these cases, circumference measurements were not made and these instances are noted in Table 1 as “Not Fully Traceable”. Diaphyseal, medullary, and CGM circumference tracings, as well as cortical area and thickness between each CGM, were quantified using the BoneJ plugin (Doube et al., 2010) for NIH ImageJ (Rasband, 1997–2012). Measurements can be found in Table 1. Geometric centroid and principal axes of transverse sections were also determined using the BoneJ plugin. Paired Student’s *t*-tests (sample for means, alpha = 0.05) were performed to compare growth mark as well as diaphyseal surface circumferences between left and right pairs of elements within an individual (Table 2). To visually represent the results of the *t*-test as well as to obtain an *R*^2^ value and a regression equation, a plot of cyclical growth marks and diaphyseal circumferences of left versus right pairs of elements from all three individuals was also constructed (Fig. 5).

**Table 2:** Results of Student’s *t*-tests performed on cyclical growth mark and diaphyseal circumference measurements of paired homologous elements. Tests were performed on cyclical growth mark and diaphyseal circumference measurements of paired homologous elements. MOR, Museum of the Rockies, Montana, USA.

Specimen number	Skeletal element	Mean difference between circumferences (mm)	Standard deviation	Number (*n*) of paired circumferences	*P*-value	Confidence level	Reject null hypothesis?
**MOR-OST 1648**	Coracoid	0.59	0.60	3	0.23	1.48	No
	Fibula	0.16	0.04	2	0.10	0.33	No
	Femur	0.16	0.00	2	0.01	0.03	No
	Humerus	0.10	0.08	2	0.34	0.75	No
	Radius	0.02	0.11	4	0.77	0.17	No
	Tibia	0.07	0.06	3	0.15	0.14	No
	Ulna	0.03	0.05	3	0.35	0.11	No
**MOR-OST 1649**	Coracoid	0.21	0.24	3	0.28	0.60	No
	Fibula	0.11	0.16	4	0.27	0.25	No
	Femur	0.57	0.51	4	0.11	0.81	No
	Humerus	0.00	0.18	4	0.99	0.29	No
	Scapula	1.38	0.46	2	0.15	4.11	No
	Tibia	0.05	0.26	4	0.74	0.42	No
	Ulna	0.00	0.15	4	0.99	0.23	No
**MOR-OST 1650**	Fibula	0.14	0.17	5	0.14	0.22	No
	Femur	0.36	0.88	5	0.41	1.09	No
	Humerus	0.09	0.12	5	0.17	0.15	No
	Radius	0.00	0.62	6	1.00	0.58	No
	Tibia	0.55	0.30	4	0.04	0.48	Yes
	Ulna	0.27	0.10	5	0.00	0.12	Yes

**Figure 6:**
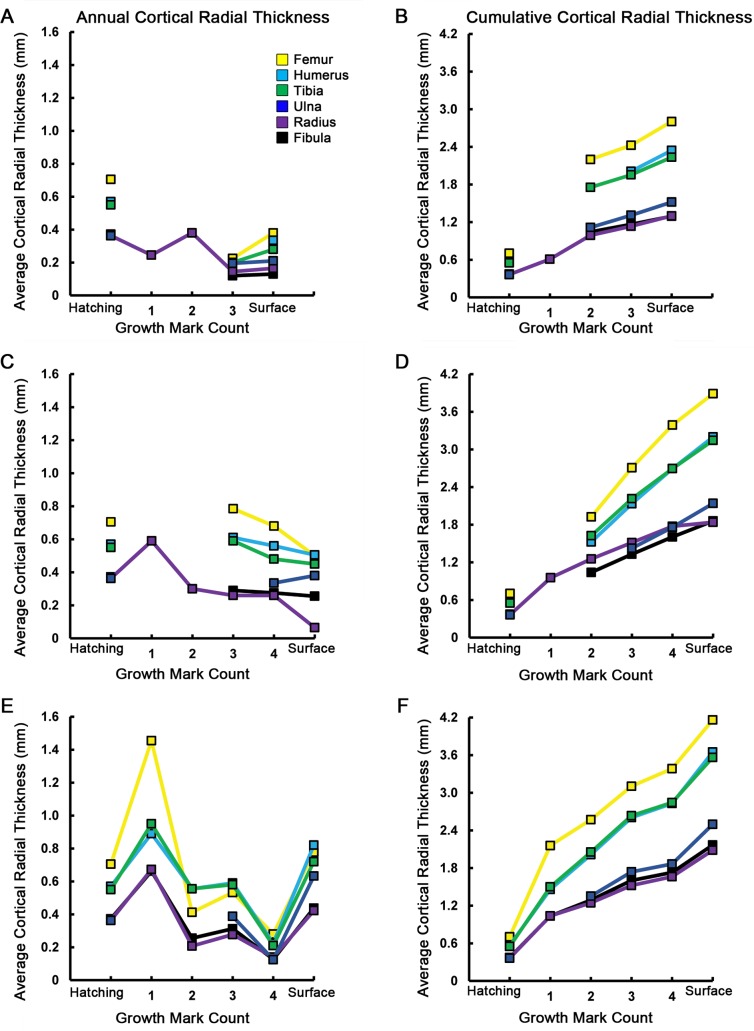
Averages and cumulative averages of annual cortical radial thicknesses. Averaged radial measurements taken along the major and minor axes from the geometric centroid to each consecutive CGM provide annual cortical radial thicknesses (A, C, E), while summing consecutive average thicknesses provides cumulative (ontogenetic) measurements (B, D, F). These measurements provide a linear record of annual and ontogenetic growth in the elements sampled. The annual increase of cortical thickness was in general greatest in the femur, humerus, and tibia of MOR-OST 1648 (A, B), MOR-OST 1649 (C, D), and MOR-OST 1650 (E, F). Considerable variability exists with regard to yearly and cumulative addition of cortical thickness when comparing homologous elements across individuals, especially between MOR-OST 1649 (C, D) and MOR-OST 1650 (E, F), the two captive alligators. Lines with no connection to the origin indicate growth marks that were either wholly or partially obliterated by medullary cavity expansion and could not be accurately measured. As exact age of the alligators is unknown, it is more appropriate to plot growth mark count rather than numerical age on the *x*-axis. Scapula and coracoid measurements were omitted because the frequent change in cross sectional shape from year to year prevented averaging of major and minor axes along cyclical growth marks.

**Figure 7:**
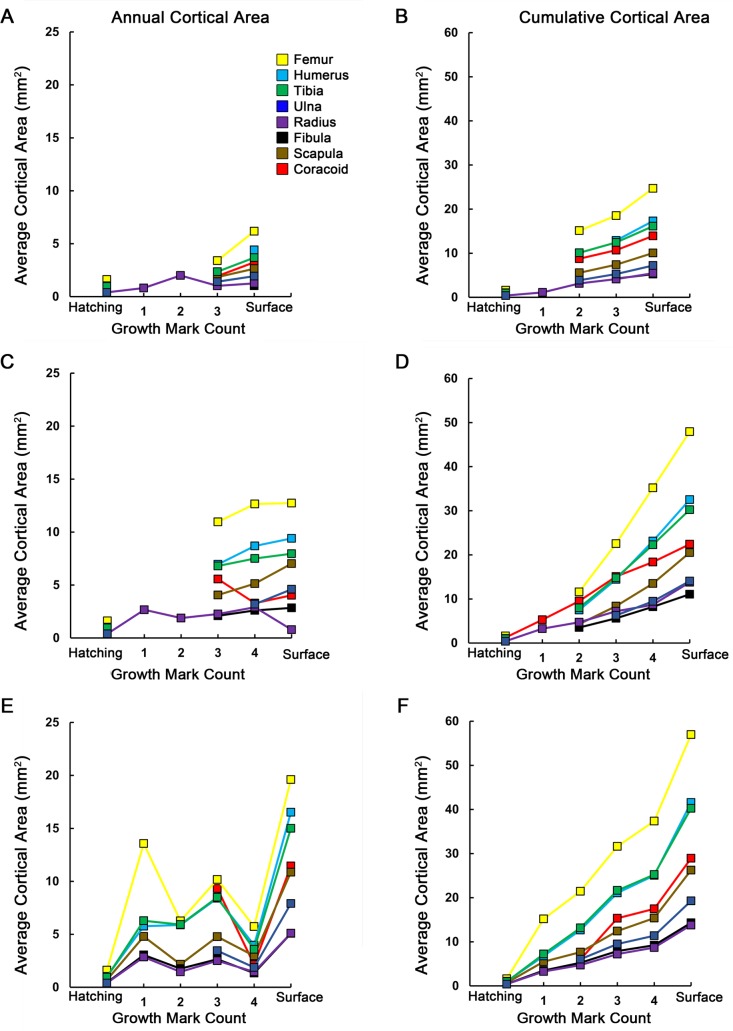
Averages and cumulative averages of annual cortical areas. Annual average cortical area is the area of a zone between two CGMs (A, C, E), while cumulative average cortical area is the additive area of zones after each year of growth (B, D, F). These measurements provide a two-dimensional record of annual and ontogenetic growth in the elements sampled. In general, skeletal elements of MOR-OST 1648 (A, B) grew at lower rates than either MOR-OST 1649 (C, D) or MOR-OST 1650 (E, F). However, the femur, tibia, and humerus consistently had the highest annual growth rates in each alligator. Lines with no connection to the origin indicate growth marks that were either wholly or partially obliterated by medullary cavity expansion and could not be accurately measured. As exact age of the alligators is unknown, it is more appropriate to plot growth mark count rather than numerical age on the *x*-axis.

Finally, average daily apposition rates were calculated (Table 3) and graphs of increase in cortical thickness as well as increase in cortical area were constructed (Figs. 6 and 7). Annual apposition rates were obtained by measuring the distance from the geometric centroid of the thin section to each successive growth mark along principal axes. At each CGM, the four measurements were averaged to obtain cumulative cortical radial thickness. Annual average cortical radial thickness was obtained by subtracting cumulative thickness at CGM *n* from the cumulative thickness at CGM *n* + 1. Average daily bone apposition rates were obtained by dividing annual average cortical radial thicknesses by an estimated 214 growing days in a year (Joanen & McNease, 1987). Annual average cortical area was similarly determined, by subtracting the cumulative cortical area enclosed by CGM *n* from the cumulative cortical area enclosed by CGM *n* + 1. If growth marks or their principal axes could not be accurately traced, corresponding measurements were omitted from the relevant data tables. Because considerable cortical drift in scapulae and coracoids prevented measurements of annual cortical thickness from a geometric centroid, apposition rates and cortical thicknesses were not determined for those elements. In some cases, a CGM was present very close to or merging with the periosteal surface. Because the zone of growth between such a CGM and the periosteal surface represents less than a year of growth (see Discussion), corresponding measurements are omitted from the graphs and apposition rate calculations.

**Table 3:** Annual average skeletal apposition rates for the three alligator individuals included in this study. An asterisk appears next to measurements in which the outermost cyclical growth mark (CGM) was omitted. MOR, Museum of the Rockies, Bozeman, Montana, USA.

Specimen number	Element	Location	Average cumulative cortical thickness (mm)	Average annual cortical thickness (mm)	Average apposition rate (µm/day)
**MOR-OST 1648**	Fibula	Hatchling	0.37	0.37	–
		CGM 1	–	–	–
		CGM 2	1.05	–	–
		CGM 3	1.17	0.12	0.56
		CGM 4	–	–	–
		Surface	1.30	0.13	0.61*
	Femur	Hatchling	0.71	0.71	–
		CGM 1	–	–	–
		CGM 2	2.20	–	–
		CGM 3	2.43	0.23	1.05
		CGM 4	–	–	–
		Surface	2.81	0.38	1.78
	Humerus	Hatchling	0.57	0.57	–
		CGM 1	–	–	–
		CGM 2	–	–	–
		CGM 3	2.01	–	–
		CGM 4	–	–	–
		Surface	2.35	0.34	1.57
	Radius	Hatchling	0.37	0.36	–
		CGM 1	0.61	0.25	1.14
		CGM 2	0.99	0.38	1.78
		CGM 3	1.14	0.15	0.68
		CGM 4	–	–	–
		Surface	1.30	0.17	0.77
	Tibia	Hatchling	0.55	0.55	–
		CGM 1	–	–	–
		CGM 2	1.76	–	–
		CGM 3	1.96	0.20	0.93
		CGM 4	–	–	–
		Surface	2.24	0.28	1.31
	Ulna	Hatchling	0.36	0.36	–
		CGM 1	–	–	–
		CGM 2	1.12	–	–
		CGM 3	1.31	0.20	0.91
		CGM 4	–	–	–
		Surface	1.52	0.21	0.98
**MOR-OST 1649**	Fibula	Hatchling	0.37	0.37	–
		CGM 1	–	–	–
		CGM 2	1.04	–	–
		CGM 3	1.33	0.29	1.36
		CGM 4	1.61	0.28	1.29
		CGM 5	–	–	–
		Surface	1.86	0.26	1.19*
	Femur	Hatchling	0.71	0.71	–
		CGM 1	–	–	–
		CGM 2	1.93	–	–
		CGM 3	2.71	0.79	3.67
		CGM 4	3.39	0.68	3.18
		CGM 5	–	–	–
		Surface	3.89	0.50	2.34*
	Humerus	Hatchling	0.57	0.57	–
		CGM 1	–	–	–
		CGM 2	1.53	–	–
		CGM 3	2.14	0.61	2.85
		CGM 4	2.70	0.56	2.62
		CGM 5	3.14	–	–
		Surface	3.20	0.50	2.36*
	Radius	Hatchling	0.37	0.36	–
		CGM 1	0.96	0.59	2.76
		CGM 2	1.26	0.30	1.40
		CGM 3	1.52	0.26	1.21
		CGM 4	1.78	0.26	1.21
		CGM 5	–	–	–
		Surface	1.84	0.06	0.30
	Tibia	Hatchling	0.55	0.55	–
		CGM 1	–	–	–
		CGM 2	1.63	–	–
		CGM 3	2.22	0.59	2.76
		CGM 4	2.70	0.48	2.24
		CGM 5	–	–	–
		Surface	3.15	0.45	2.10*
	Ulna	Hatchling	0.36	0.36	–
		CGM 1	–	–	–
		CGM 2	–	–	–
		CGM 3	1.43	–	–
		CGM 4	1.76	0.34	1.57
		CGM 5	2.05	–	–
		Surface	2.14	0.38	1.78*
**MOR-OST 1650**	Fibula	Hatchling	0.37	0.37	–
		CGM 1	1.04	0.66	3.10
		CGM 2	1.29	0.26	1.19
		CGM 3	1.60	0.31	1.46
		CGM 4	1.73	0.13	0.60
		CGM 5	–	–	–
		Surface	2.17	0.44	2.04
	Femur	Hatchling	0.71	0.71	–
		CGM 1	2.16	1.46	6.80
		CGM 2	2.57	0.41	1.93
		CGM 3	3.11	0.53	2.49
		CGM 4	3.39	0.28	1.31
		CGM 5	–	–	–
		Surface	4.16	0.78	3.63
	Humerus	Hatchling	0.57	0.57	–
		CGM 1	1.46	0.89	4.16
		CGM 2	2.02	0.56	2.59
		CGM 3	2.61	0.59	2.76
		CGM 4	2.83	0.23	1.05
		CGM 5	–	–	–
		Surface	3.65	0.82	3.83
	Radius	Hatchling	0.37	0.36	–
		CGM 1	1.04	0.67	3.14
		CGM 2	1.25	0.21	0.97
		CGM 3	1.52	0.28	1.30
		CGM 4	1.66	0.14	0.65
		CGM 5	2.04	–	–
		Surface	2.09	0.42	1.97*
	Tibia	Hatchling	0.55	0.55	–
		CGM 1	1.50	0.95	4.44
		CGM 2	2.06	0.56	2.59
		CGM 3	2.64	0.58	2.71
		CGM 4	2.85	0.21	0.98
		CGM 5	–	–	–
		Surface	3.57	0.72	3.36
	Ulna	Hatchling	0.36	0.36	–
		CGM 1	–	–	–
		CGM 2	1.35	–	–
		CGM 3	1.74	0.39	1.81
		CGM 4	1.87	0.13	0.58
		CGM 5	2.45	–	–
		Surface	2.50	0.63	2.96*

Estimates of neonate minimum diaphyseal circumferences and cortical areas were obtained by measuring thin sections from the hatchling alligator MOR-OST 1647. Although the numerical ages of the juvenile alligators are unknown, the diaphyseal circumferences of skeletal elements from the neonate alligator establish whether any CGMs were lost to medullary expansion in the juveniles: if the medullary cavity circumference from a juvenile bone was larger than the diaphyseal circumference of the homologous hatchling bone, the possibility that medullary expansion destroyed the earliest CGMs was considered. Based on this comparison, in at least one skeletal element in each individual the CGM record could be tracked from the end of the first year until termination. These complete growth records provide starting points from which to retrocalculate the number of missing CGMs in other elements from the same individual.

## Results

The bone tissue microstructure of crocodylians is extensively studied. Analyses particularly relevant to our study include de Ricqlès (1976), who observed CGMs within a cortex of parallel fibered and sometimes woven tissue with longitudinal vascular canals, and more recently, Lee (2004) provided a detailed ontogenetic description of *A. mississippiensis* femoral microstructure and found predominately longitudinal vascularity in parallel fibered tissue; still other researchers reported woven fibered tissue in bones of both captive and wild alligators (Chinsamy & Hillenius, 2004; Padian, Horner & Ricqlès, 2004; Reid, 1984; Reid, 1997; Tumarkin-Deratzian, 2007); studies of captive Siamese crocodiles (de Buffrenil, 1980; de Buffrénil & Castanet, 2000) revealed a close correlation between the number of CGMs and individual age, and showed osteoderms are useful for estimating the age of wild crocodylians (Hutton, 1986; Tucker, 1997); a study by Klein, Scheyer & Tütken (2009) compared the skeletochronology recorded in the osteoderms and limbs of a skeletally mature captive female alligator and confirmed that osteoderms are poor age indicators in breeding females, while also reporting on tissue organization ranging from lamellar to poorly organized parallel fibered bone.

Several observations summarize the bone microstructure patterns seen in our thin section samples, and many of these observations support the findings of the aforementioned studies. Medullary cavities are sometimes enclosed by an endosteal layer consisting of flattened osteocytes embedded within highly organized lamellar tissue (i.e., inner circumferential lamellae). Bone tissue organization found in elements of the early juvenile alligators includes lamellar, parallel fibered, loosely parallel fibered, and even woven, although the predominant tissue organization often varies within and across elements. Regions of woven tissue were found within the humerus, ulna, femur, tibia, and fibula of wild-captured MOR-OST 1648, but only in the zone between the first and second CGM. Elements of MOR-OST 1649 were completely lacking woven tissue, and only the tibia of MOR-OST 1650 contained regions of woven tissue between the first and second CGM, as well as between the fourth CGM and the periosteal surface.

Vascular canals are incorporated directly into the bone matrix (simple primary canals) or encircled by lamellar tissue (primary osteons; see Discussion). They are most often oriented longitudinal to the transverse plane of section, but are frequently connected by radial or oblique anastomosing vascular canals.

Thick fiber bundles are scattered throughout the cortex and most are arranged radially, but some are also circumferential to the transverse plane of section. Radial fibers are often especially concentrated along the circumference of cortical growth marks. These fibers are commonly referred to as Sharpey’s fibers, although it must be noted that histological differentiation between fibers anchoring tendon to bone, and fibers anchoring periost to bone, is difficult (Hall, 2005). While we emphasize there is continued debate over how the term should be applied, we use Sharpey’s fibers in the general sense, encompassing all attachment fibers found embedded within limb bone cortex (Francillon-Vieillot et al., 1990; Hall, 2005; Ham & Cormack, 1979).

Cyclical growth marks are present in all specimens in the form of either lines of arrested growth (LAGs) or annuli. No pattern related to ontogeny or captivity status is evident regarding which kind of growth marks, annuli or LAGs, are formed within the bone. CGMs in the form of LAGs were most commonly found in the ulna, fibula, and coracoid, but not restricted to these elements. Annuli are more frequent than LAGs across all specimens.

To determine if enough medullary expansion occurred to fully destroy the oldest CGMs in a bone and result in intraskeletal CGM count discrepancies, diaphyseal circumferences of hatchling alligator (MOR-OST 1647) thin sections were compared with the medullary circumferences of homologous elements from each of the three juvenile alligators (Table 1). In many elements, the circumference of the medullary cavity is less than or nearly equal to the periosteal surface circumference of homologous hatchling elements. In such instances no growth marks are completely destroyed by bone resorption associated with medullary cavity enlargement, so that the innermost CGM observed is the first to have formed.

Because thin section processing was done by hand, sometimes one thin section out of several made from an element was polished thinner than the others. It was only in sections polished too thin that CGMs became vague and difficult to trace, seeming to disappear into the surrounding bone fabric. When this occurred, often slides stained with Toluidine blue aided in locating the faint marks. Our observations suggest that researchers should consider errors in procedure when observing inconsistent intraskeletal CGM counts if medullary expansion or secondary remodeling is not evident.

### Qualitative descriptions

The general bone histology of crocodylians is well known, but descriptions of bone microstructure specific to each appendicular element are lacking. Therefore, detailed qualitative histologic descriptions and figures of each skeletal element examined are provided in the Supplemental Information. Unless noted, no great differences in tissue organization were observed between left and right element pairs in the same individual.

### Quantification of results

Quantitative analyses document trends in skeletal growth rates and variability. A Student’s *t*-test (Table 2) was performed on paired element measurements from all three alligators, for a total of twenty pairs. With only two exceptions (the tibia and ulna of MOR-OST 1650), there was no significant difference in the circumferences of CGMs or in diaphyseal circumferences between left and right pairs in each individual. Plotting left versus right CGMs and diaphyseal surface circumferences for each element (Fig. 5) results in a fitted linear curve (*y* = 1*x*−0.047) producing an *R*^2^ value of 0.98. Additionally, average daily bone apposition rates (Table 3), annual and cumulative average cortical radial thicknesses (Fig. 6), and annual and cumulative average cortical areas (Fig. 7) demonstrate trends as well as variability in intraskeletal bone growth across individuals.

## Discussion

### Woven tissue

Woven tissue is commonly found in dinosaurs, birds, and large-bodied mammals, and occurs infrequently in crocodylians. When present in crocodylians, it is usually found in individuals raised in artificially optimum conditions encouraging fast growth, or in fast growing early juveniles (Padian, Horner & Ricqlès, 2004). Woven tissue has also been reported in wild alligators (Chinsamy & Hillenius, 2004; Reid, 1984; Reid, 1997; Tumarkin-Deratzian, 2007). However, woven tissue deposition is not sustained throughout ontogeny and a strong “fibrolamellar complex” like that observed in dinosaurs, birds, and mammals is not present. It is interesting that a wild alligator (MOR-OST 1648), living in sub-optimal conditions, produced woven tissue in more elements than either MOR-OST 1649 or MOR-OST 1650, which were raised in captivity. Differences in tissue organization spanning both captive and wild animals therefore suggest individual variation in growth unrelated to environmental stresses.

**Figure 8:**
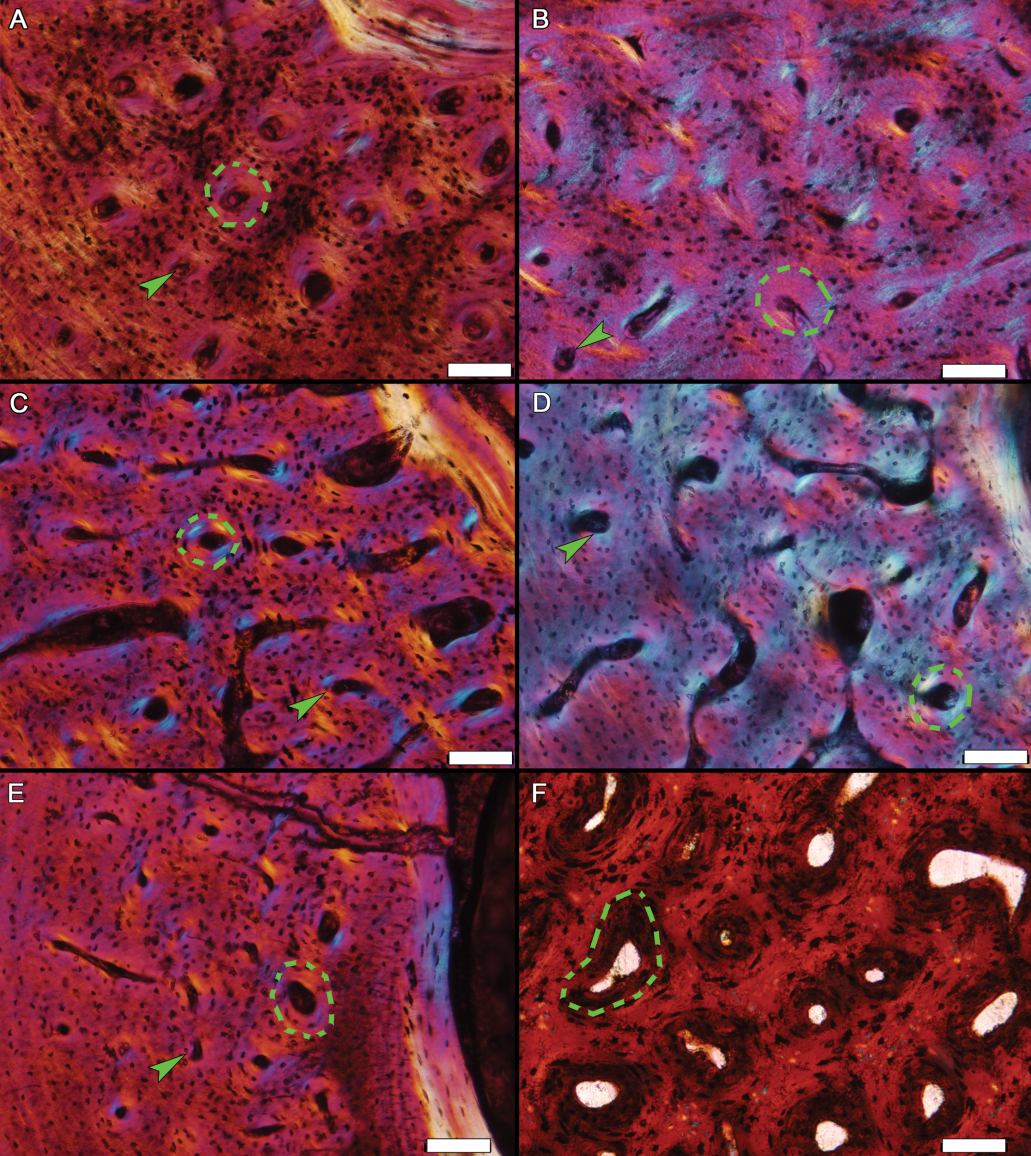
Examples of woven tissue and primary osteons within diaphyseal sections. Primary osteons (examples in dashed outlines) are embedded within a woven tissue matrix in (A) the ulna of MOR-OST 1648, (B) the tibia of MOR-OST 1650, (C) the humerus of MOR-OST 1648, (D) the tibia of MOR-OST 1648, (E) the fibula of MOR-OST 1648, and (F) the tibia of the non-avian dinosaur *Maiasaura* (MOR 005 T11-3) for comparison. The primary osteons of the alligators have fewer lamellae and are more acellular than those from the *Maiasaura*. Simple primary canals (examples indicated by arrows) are scattered amongst the primary osteons in the alligator tissue but are not present in the *Maiasaura* section. Scale bars, 100 µm.

The woven-fibered regions within elements of MOR-OST 1648 and MOR-OST 1650 not only contain simple primary canals, but also vessels enclosed by a nearly acellular, lamellated parallel fibered tissue (i.e., primary osteons) (Fig. 8). The combination of woven-fibered tissue and primary osteons, which is previously reported in crocodylians (e.g., Chinsamy & Hillenius, 2004; de Ricqlès, 1983; de Ricqlès, Padian & Horner, 2001; Horner, Padian & Ricqlès, 2001; Padian, Horner & Ricqlès, 2004; Reid, 1984; Reid, 1987; Reid, 1990; Tumarkin-Deratzian, 2007), is commonly described as fibrolamellar. In our study, the areas of woven tissue are patchy or discontinuous, and simple primary canals are also scattered amongst the primary osteons. Simple primary canals are rarely, if ever, reported in well-developed fibrolamellar tissue. Also, primary osteons found within fibrolamellar tissue of many non-avian dinosaurs (Fig. 8F) and extant endotherms are relatively large structures made of multiple lamellae, often encompassing more than one vascular canal, and contain numerous osteocytes (Padian & Horner, 2004). This contrasts with the small size of the alligator primary osteons, which contain few lamellae and infrequent osteocytes. Klein (2010) and Konietzkoo-Meier & Klein (2013) described similar structures in the early juvenile bone tissues of certain eusauropterygians and temnospondyls, respectively. These authors termed such tissue “incipient fibrolamellar”. Perhaps the incipient fibrolamellar tissue in the alligators observed here and in previous alligator studies results from temporary, unsustained rapid growth in those individuals. In this context, the presence of such tissue organization supports the hypothesis that under certain conditions crocodylians retain the primitive archosaur capacity for growing at elevated rates (Cubo et al., 2012; Legendre, Segalen & Cubo, 2013), possibly as a result of their efficient respiratory system (Cubo et al., 2012; Farmer & Sanders, 2010). On the other hand, existence of this incipient fibrolamellar tissue in temnospondyls implies the ability for brief rapid growth in predominantly slow growing taxa may be a primitive characteristic of tetrapods in general.

Regardless of the evolutionary history or causes of incipient fibrolamellar tissue formation, it is important to distinguish characteristics associated with temporary rapid growth observed in alligators from tissue characteristics associated with sustained rapid growth observed in many mammals and birds today. Unfortunately, use of the term “fibrolamellar” has become quite subjective in recent years (Stein & Prondvai, 2014) as authors infrequently define or provide images to explain their intended meaning. To avoid confusion, it is best to clearly define the terminology used or to refrain from subjective terms such as fibrolamellar in favor of simply describing tissue organization and structures present (Werning, 2012).

### Cyclical growth marks

Generally, the kind of CGMs present (i.e., LAG, annulus, or annulus followed by a LAG) vary individually and by year rather than corresponding to a particular element, tissue arrangement, or even to captivity status. Variation in CGM number was observed across different elements from an individual but CGM count was consistent amongst the serial thin sections taken from the diaphysis of the same bone.

**Figure 9:**
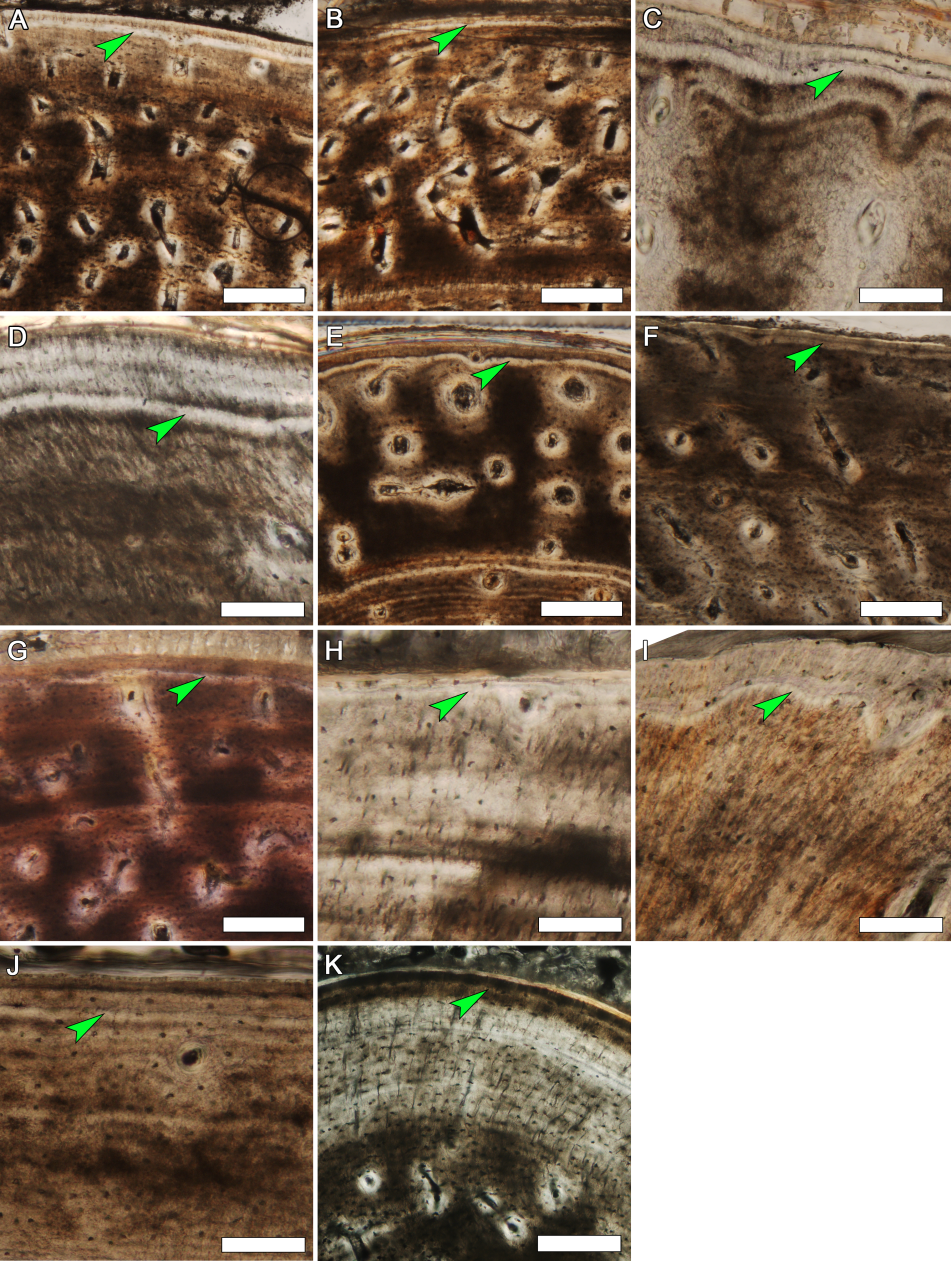
Detail of the outer cortex from elements of MOR-OST 1649 and MOR-OST 1650 showing a fifth cyclical growth mark. An arrow indicates this outermost fifth growth mark, observed in many elements of the two captive alligators. (A) the humerus of MOR-OST 1649. Scale bar, 500 µm; (B) the humerus of MOR-OST 1650. Scale bar, 500 µm; (C) the radius of MOR-OST 1650. Scale bar, 100 µm; (D) the ulna of MOR-OST 1649. Scale bar, 100 µm; (E) the ulna of MOR-OST 1650. Scale bar, 500 µm; (F) the femur of MOR-OST 1649. Scale bar, 500 µm; (G) the tibia of MOR-OST 1649. Scale bar, 500 µm; (H) the fibula of MOR-OST 1649. Scale bar, 100 µm; (I) the scapula of MOR-OST 1649. Scale bar, 100 µm; (J) the scapula of MOR-OST 1650. Scale bar, 100 µm.

Several bones from the captive alligators displayed an annulus very close to, and often merging with, the periosteal surface while other bones (including contralateral bones of the same individual in some cases) lacked this extra mark. This particular discrepancy likely has a simple explanation. Alligators in Louisiana annually enter a state of greatly decreased activity and appetite beginning in October and ending in March regardless of captivity status (Chabreck & Joanen, 1979; Coulson, Coulson & Hernandez, 1973; Joanen & McNease, 1987). Specimens used in this study were terminated during the month of February. It is possible that as the alligators began to emerge from their torpid state some elements resumed apposition before others, making an extra CGM visible due to the small addition of new tissue (Fig. 9). Our preliminary findings suggest this is an avenue for focused research as this hypothesis has implications for interpreting CGMs found near the periosteal surface in fossils.

**Figure 10:**
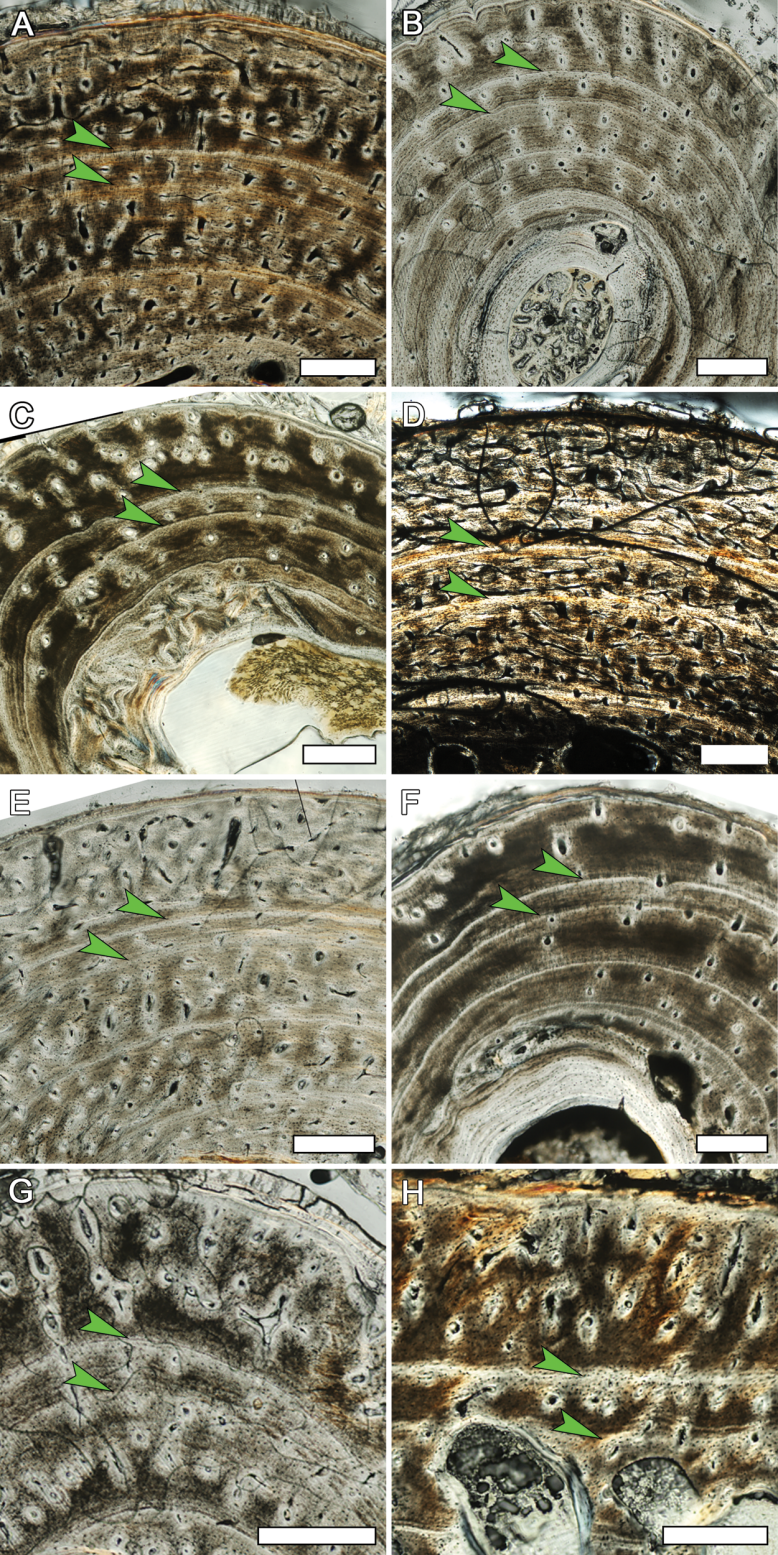
The third and fourth cyclical growth marks in every sampled element of MOR-OST 1650. These growth marks are unusually closely spaced in all sampled elements, providing a natural “marker” within the cortex. They are indicated by arrows in (A) the humerus, (B) radius, (C) ulna, (D) femur, (E) tibia, (F) fibula, (G) coracoid, and (H) scapula. Scale bars, 100 µm.

Previous histologic studies on reptiles reveal that medullary expansion results in CGM count discrepancies (Griffiths, 1961; Hutton, 1986), and that in older crocodylians and particularly in breeding females, secondary remodeling (Schweitzer et al., 2007) and conversion from compact to cancellous bone (Hutton, 1986) also contributes to CGM loss. Due to the early juvenile status of the alligators in our study, no significant amount of secondary remodeling within the cortex or conversion of cortex to cancellous bone was observed. Only in instances where the medullary cavity circumference was larger than the hatchling diaphyseal circumference did a reduced number of CGMs occur, suggesting that medullary expansion accounts for differences we observed in CGM number within the cortex across elements. This observation was independently tested using the bone microstructure of MOR-OST 1650. In every bone the third and fourth CGMs were closely spaced, outlining a narrow zone of primary tissue within the cortex (Fig. 10). This is not considered an instance of a single hiatus represented by a “double LAG” (Caetano & Castanet, 1993; Castanet et al., 1993) because the tissue organization within the narrow zone indicates no significant decrease in apposition occurred during that time. Additionally, the two CGMs do not follow a similar pathway as is typical in double LAGs and are instead independent of each other. This relatively small zone may instead indicate a particularly long period of arrested growth during the fourth year of life. In this way the narrow zone provides a natural “label”, so that even if medullary expansion obliterated inner CGMs, the number missing could be determined using the narrow zone as a landmark and complete growth records from other bones of MOR-OST 1650 as guides. The histology of MOR-OST 1650 therefore provides strong evidence that it is the action of medullary expansion (as well as cortical remodeling in older animals) causing CGM counts to vary within the cortex, and it is not because some elements form CGMs yearly while others do not.

Although tissue organization and cyclical growth mark counts varied across different skeletal elements within an individual, Sharpey’s fibers (e.g., Fig. S1) were present in every thin section examined. The fibers were often especially dense near CGMs. During the period of slowed or arrested growth indicated by CGMs, the periosteum and mineralization front were essentially stationary. Since Sharpey’s fibers anchor the periosteum and tendons to the bone matrix (Francillon-Vieillot et al., 1990; Hall, 2005; Ham & Cormack, 1979), it follows that there would be a concentration of fibers if the periosteum was periodically static. Locating dense bands of Sharpey’s fibers may therefore aid in identifying vague or faint growth marks both in extant and extinct animals.

### Statistical analyses and growth curves

Because of the immature nature of the alligator specimens, the number of corresponding left and right CGMs as well as left and right diaphyseal circumference measurements (*n*) included in the Student’s *t*-test of each skeletal element was low (the largest sample set was *n* = 6), making the chance for statistical errors quite high (including incorrect rejection of the null hypothesis of no difference (Type I) and failure to reject a false null hypothesis (Type II)). Out of twenty paired elements tested only the tibia and ulna from MOR-OST 1650 rejected the null hypothesis of no difference between left and right CGM circumferences (Table 2). In the case of the tibia, the right sample measured was located more proximal along the diaphysis relative to the sample measured from the left tibia, which may account for the discrepancy. Because eighteen of the twenty pairs statistically demonstrated a result of no difference, it is very possible that rejection of the null hypothesis for the ulna of MOR-OST 1650 is the result of a Type I error.

In addition to a *t*-test, left versus right CGM and surface circumference measurements for each skeletal element sampled were plotted to assess any relationship between the measurements (Fig. 5). The result (*R*^2^ = 0.984) provides the first quantitative validation that there is no appreciable variation in CGM circumferences or surface diaphyseal circumferences between left and right pairs in an individual if sampled from roughly the same location.

Average daily apposition rates were determined for each element from an individual (Table 3) assuming a 214 day growing season (Joanen & McNease, 1987). Rates ranged between 0.3 and 6.8 µm/day, which is consistent with measurements obtained in previous crocodylian studies (Cubo et al., 2012; Padian, Horner & Ricqlès, 2004; Padian, Ricqlès & Horner, 2001; Roberts et al., 1988). The femur, humerus, and tibia achieved the highest annual rates within each individual. The highest apposition rate recorded was 6.8 µm/day, from the femur of MOR-OST 1650 during its first year of life. Comparing skeletal apposition rates (Table 3), annual cortical radial thickness (Fig. 6), and annual cortical area (Fig. 7) from the three alligators shows the growth rate of wild-captured MOR-OST 1648 was in general lower than either captive raised MOR-OST 1649 or MOR-OST 1650. Captive alligators provided with a reliable food source grow more rapidly than their wild counterparts, often attaining twice the length of a wild alligator of the same age in a single year (de Ricqlès, 1983). Regardless, there was variability in rates both within and across individuals. Apposition rates for all elements of wild-caught MOR-OST 1648 at the end of the third year of growth were lower than those for either captive alligator, but at the end of the fourth year several elements of MOR-OST 1648 had higher apposition rates than corresponding elements of MOR-OST 1650. This is likely due to the unusually long growth hiatus experienced by MOR-OST 1650 during the fourth year, as this would artificially depress the apposition rate.

In elements containing a complete annual growth record, apposition rates were highest during the first year of growth and generally did not return to those rates during the time interval recorded in this study. The exception to this observation is the radius of MOR-OST 1648, in which the highest apposition rate (1.78 µm/day) occurred during the second year of life. The second year of life is also when woven tissue was present in many elements of MOR-OST 1648, although not in the radius. Trends in annual average cortical area often mirrored those observed in annual average apposition and cortical radial thickness measurements. For example, elements with high annual average cortical thicknesses also had higher annual average cortical areas, but this relationship does not always hold true. Although cortical thickness decreased in every element except the ulna during the fifth year of life in MOR-OST 1649, cortical area was still higher than the previous year for every element except the radius. This is because elements with larger circumferences (e.g., femur, humerus, tibia) require only a small addition of cortex to increase in cortical area while elements with smaller circumferences, such as the radius, require a large addition to cortical thickness to greatly increase cortical area.

These quantitative data are supported by bone fiber organization. The femur, tibia, and humerus had the highest apposition rates and were the most vascularized with either parallel fibered tissue or a combination of parallel fibered and woven tissue. The radius and fibula had the lowest yearly apposition rates in each individual, correlating with reduced vascularity and highly organized lamellar or parallel fibered tissue.

Based on osteohistology the femur, tibia, and humerus are most useful for determining the maximum growth rates of alligators. These elements did tend to have considerable medullary expansion, however, making them less desirable for skeletochronology. Comparatively less medullary expansion and drift resulted in a consistently high number of complete CGMs in the radius, making it preferable for alligator skeletochronology. The ulna, scapula, and coracoid also resembled the radius in tissue organization, but medullary expansion and drift often obscured or obliterated inner CGMs, making these elements less desirable for skeletochronology. But as demonstrated by MOR-OST 1650, it is possible for many elements to retain inner CGMs for an extended duration, suggesting rates of medullary expansion also vary considerably between homologous elements in different individuals.

### Osteoderms

Unlike endochondral skeletal elements, growth data were not successfully collected from osteoderms. Hutton (1986), as well as Tucker (1997), achieved success by using osteoderms for skeletochronology in older, larger, and less actively growing crocodylians, but both authors noted that the osteoderms of breeding females often experienced greater conversion of compact bone to cancellous bone which obscured growth marks. Osteoderms were poor indicators of age in the present study because the disorganized tissue and abundant Sharpey’s fibers obscured growth marks. This disorganization was likely due in part to the immature status of the individuals, but may also be due to sectioning transversely across the keel rather than parallel to it. Our result implies that the processes obscuring CGMs in the young alligators (tissue disorganization and abundant Sharpey’s fibers) are different than the processes resulting in obfuscation of CGMs in adults, especially in the case of sexually mature females. The histology does demonstrate the highly porous and richly vascularized nature of osteoderms, supporting hypotheses that besides armored defense, osteoderms serve additional functions such as aiding in thermoregulation or for muscular bracing (e.g., Farlow, Hayashi & Tattersall, 2010; Frey, 1988).

## Conclusions

Our study addressed intraskeletal variability in alligator bone microstructure and also compared the growth rates and microstructures of homologous elements across three alligator individuals. The small interskeletal sample size of three means additional studies are needed to confirm that observations hold true for crocodylians and vertebrates in general, but our preliminary results hint at the potential extent of individual variation that may go unaccounted for in bone tissue microanalyses.

Individual intraskeletal variability is seldom addressed in fossil or modern bone histology, but is an important factor to consider when making growth history generalizations based on bone microstructure. Extant vertebrate intraskeletal studies aid in understanding individual variability as well as help validate long-standing paleohistologic assumptions. Using *Alligator mississipiensis* skeletal elements, we demonstrated that the intraskeletal CGM count discrepancies reported in previous paleohistology studies are largely the result of differing medullary expansion rates and secondary remodeling, and not because CGMs form inconsistently from year to year. Therefore CGMs do seem a reliable form of skeletochronology independent of appendicular element sampled, provided that medullary cavity expansion rate and secondary remodeling is slow, or that age retrocalculations are based on superimposing an ontogenetic series of CGMs to reconstruct growth history.

Medullary expansion rates and secondary remodeling may affect CGM counts between elements in an individual, but CGM counts were consistent across serial thin sections taken within the diaphysis of the same bone. Diaphyseal surface circumferences, cortical CGM circumferences, and cortical histology were also consistent between left and right homologous elements within an individual, providing quantitative evidence for long-held paleohistology assumptions.

We also demonstrate that the humerus, tibia, and femur are optimal for studying maximum alligator growth rates while the radius is more useful for skeletochronology. Thus, there are likely optimal elements in other vertebrate taxa for use in growth rate studies or skeletochronology.

*Alligator mississippiensis* intraskeletal bone tissue microstructures were described and quantified to validate preexisting assumptions used by paleohistologists for interpreting the growth histories of extinct taxa. The validations presented here therefore contribute to the quantitative framework necessary for confirming the importance and reliability of CGMs and tissue organization in studies on any vertebrate taxon, living or extinct.

## Supplemental Information

10.7717/peerj.422/supp-1 Supplemental Information 1 Qualitative descriptions of *Alligator mississippiensis* osteohistology

10.7717/peerj.422/supp-2 Table S1 MorphoBank P731 accession numbers for high-resolution images of Alligator skeletal elements and manuscript figures. MOR, Museum of the Rockies, Montana, USA

10.7717/peerj.422/supp-3 Figure S1 Osteohistology of the mid-diaphyseal humerus of alligator MOR-OST 1648. (A) Transverse section Vascular canal density is low in the mid cortex and higher towards the periosteal surface. The arrow indicates periosteal attachment fibers running parallel to growth mark. Scale bar, 1 mm. (B) Enlargement of the area from (A) within the green box, photographed using a full lambda (530 nm) plate to reveal fiber orientation, but which also tends to obscure growth marks. The endosteal layer (green arrow) cuts across the well vascularized and woven primary tissue of the inner cortex. The mid cortex is less well vascularized and is parallel-fibered, while the outer cortex is again well vascularized but remains parallel-fibered. Periosteal attachment fibers (white arrow) are arranged perpendicular to bone tissue orientation. Scale bar, 500 µm.

10.7717/peerj.422/supp-4 Figure S2 Osteohistology of the mid-diaphyseal humerus of alligator MOR-OST 1649 (A) Transverse section. Scale bar, 1 mm. (B) Enlargement of the area from (A) within the green box, photographed using a full lambda (530 nm) plate to reveal fiber orientation. Three growth marks are in view (green arrows). Resorption cavities (white arrow) are present near the medullary cavity, and tissue is parallel-fibered. Vascular canal density is uniform throughout the cortex, consisting of longitudinal as well as obliquely anastomosing canals. Scale bar, 500 µm.

10.7717/peerj.422/supp-5 Figure S3 Osteohistology of the mid-diaphyseal humerus of alligator MOR-OST 1650 (A) Transverse section. Scale bar, 1 mm. (B) Enlargement of the area from (A) within the green box, photographed using a full lambda (530 nm) plate to reveal fiber orientation, but which also tends to obscure growth marks. Regardless, three growth marks (arrows) are visible in this portion of the cortex. The cortex is parallel-fibered throughout, and vascular density is uniform. Vascular orientation is mostly longitudinal within the inner cortex, becoming predominately obliquely anastomosing by mid cortex. Scale bar, 500 µm.

10.7717/peerj.422/supp-6 Figure S4 Osteohistology of the mid-diaphyseal radius of alligator MOR-OST 1648 (A) Transverse section. Scale bar, 1 mm. (B) Enlargement of the area from (A) within the green box, photographed using a full lambda (530 nm) plate to reveal fiber orientation. Two growth marks are visible in this enlargement (arrows). The cortex is of highly organized lamellar tissue with a sparse scattering of longitudinal vascular canals. Scale bar, 100 µm.

10.7717/peerj.422/supp-7 Figure S5 Osteohistology of the mid-diaphyseal radius of alligator MOR-OST 1649 (A) Transverse section. Scale bar, 1 mm. (B) Enlargement of the area from (A) within the green box, photographed using a full lambda (530 nm) plate to reveal fiber orientation. The cortex is of highly organized lamellar tissue and is nearly avascular. Green arrows point to four LAGs in this region. A well-developed endosteal layer (left) cuts across primary tissue and in places consists of secondary osteons, indicating medullary drift. Scale bar, 500 µm.

10.7717/peerj.422/supp-8 Figure S6 Osteohistology of the mid-diaphyseal radius of alligator MOR-OST 1650 (A) Transverse section. The well-developed endosteal layer cuts across primary tissue and in some areas is replaced by secondary osteons, indicating medullary drift. Scale bar, 1 mm. (B) Enlargement of the area from (A) within the green box, photographed using a full lambda (530 nm) plate to reveal fiber orientation. The cortex is made of highly organized lamellar tissue containing scattered longitudinal simple primary canals and primary osteons. Three CGMs (arrows) are evident in the enlargement. Scale bar, 100 µm.

10.7717/peerj.422/supp-9 Figure S7 Osteohistology of the mid-diaphyseal ulna of alligator MOR-OST 1648 (A) Transverse section. Periosteal attachment fibers (arrow) are evident even at low magnification. Scale bar, 1 mm. (B) Enlargement of the area from (A) within the green box, photographed using a full lambda (530 nm) plate to reveal fiber orientation. Here the cortex is lamellar, with sparsely scattered longitudinal vascular canals. The innermost LAG is partially destroyed by medullary expansion and two more are visible within the cortex (green arrows). Scale bar, 100 µm.

10.7717/peerj.422/supp-10 Figure S8 Osteohistology of the mid-diaphyseal ulna of alligator MOR-OST 1649 (A) Transverse section. The high degree of endosteal remodeling is apparent even at low magnification, especially in the region highlighted within the green box. Scale bar, 1 mm. (B) Enlargement of the area from (A) within the green box, photographed using a full lambda (530 nm) plate to reveal fiber orientation. Secondary reconstruction occurred within the inner cortex due to medullary drift, resulting in large, overlapping secondary osteons. Scale bar, 100 µm.

10.7717/peerj.422/supp-11 Figure S9 Osteohistology of the mid-diaphyseal ulna of alligator MOR-OST 1650 (A) Transverse section. Scale bar, 1 mm. (B) Enlargement of the area from (A) within the green box, photographed using a full lambda (530 nm) plate to reveal fiber orientation. Two LAGs are marked by green arrows. Medullary expansion was active in this region as indicated by the partially resorbed endosteal layer (white arrow) and resorption cavity within the inner cortex. Tissue organization within the cortex is lamellar, and vascularity is longitudinal. Scale bar, 500 µm.

10.7717/peerj.422/supp-12 Figure S10 Osteohistology of the mid-diaphyseal femur of alligator MOR-OST 1648 (A) Transverse section shows a combination of anastomosing and radial vascular canals. Scale bar, 1 mm. (B) Enlargement of the area from (A) within the green box, photographed using a full lambda (530 nm) plate to reveal fiber orientation. Tissue within the innermost cortex is woven but is parallel-fibered by mid cortex. Scale bar, 500 µm.

10.7717/peerj.422/supp-13 Figure S11 Osteohistology of the mid-diaphyseal femur of alligator MOR-OST 1649 (A) Transverse section, stained with Toluidine chloride. Scale bar, 1 mm. (B) Enlargement of the area from (A) within the green box, photographed using a full lambda (530 nm) plate to reveal fiber orientation. The cortical tissue is loosely parallel-fibered. Scale bar, 500 µm.

10.7717/peerj.422/supp-14 Figure S12 Osteohistology of the mid-diaphyseal femur of alligator MOR-OST 1650 (A) Transverse section photographed using circularly polarized light. Medullary drift is evident by the concentration of secondary tissue endosteally, and active medullary expansion is revealed by large resorption cavities within the inner cortex. Scale bar, 1 mm. (B) Enlargement of the area from (A) within the green box, photographed using a full lambda (530 nm) plate to reveal fiber orientation. The cortical tissue is loosely parallel-fibered. Scale bar, 500 µm.

10.7717/peerj.422/supp-15 Figure S13 Osteohistology of the mid-diaphyseal tibia of alligator MOR-OST 1648 (A) Transverse section. Scale bar, 1 mm. (B) Enlargement of the area from (A) within the green box, photographed using a full lambda (530 nm) plate to reveal fiber orientation. Fibers are woven within the inner cortex but are parallel-fibered by mid cortex. Vascular canals are obliquely anastomosing. Scale bar, 500 µm.

10.7717/peerj.422/supp-16 Figure S14 Osteohistology of the mid-diaphyseal tibia of alligator MOR-OST 1649 (A) Transverse section, stained with Toluidine chloride. Scale bar, 1 mm. (B) Enlargement of the area from (A) within the green box, photographed using a full lambda (530 nm) plate to reveal fiber orientation. Medullary expansion is evident by the resorbed region of inner cortex (white arrow) and the partial destruction of the innermost annulus (green arrow). Two more annuli (green arrows) are present within the cortex. Tissue is parallel-fibered and most vascular canals are either longitudinal or somewhat anastomosing. Scale bar, 500 µm.

10.7717/peerj.422/supp-17 Figure S15 Osteohistology of the mid-diaphyseal tibia of alligator MOR-OST 1650 (A) Transverse section. Scale bar, 1 mm. (B) Enlargement of the area from (A) within the green box, photographed using a full lambda (530 nm) plate to reveal fiber orientation. Here, tissue fibers are arranged loosely in parallel. Vascular canals are longitudinal and anastomosing. Scale bar, 500 µm.

10.7717/peerj.422/supp-18 Figure S16 Osteohistology of the mid-diaphyseal fibula of alligator MOR-OST 1648 (A) Transverse section. The innermost cortex is partially destroyed by medullary drift. Scale bar, 1 mm. (B) Enlargement of the area from (A) within the green box, photographed using a full lambda (530 nm) plate to reveal fiber orientation. This region is largely avascular and the lamellar tissue contains numerous periosteal attachment fibers. Scale bar, 100 µm.

10.7717/peerj.422/supp-19 Figure S17 Osteohistology of the mid-diaphyseal fibula of alligator MOR-OST 1649 (A) Transverse section. Scale bar, 1 mm. (B) Enlargement of the area from (A) within the green box, photographed using a full lambda (530 nm) plate to reveal fiber orientation. Highly organized lamellar fibers contain scattered osteocytes. Periosteal attachment fibers are abundant. Circles on the right of the image are preparation artifacts. Scale bar, 100 µm.

10.7717/peerj.422/supp-20 Figure S18 Osteohistology of the mid-diaphyseal fibula of alligator MOR-OST 1650 (A) Transverse section. Medullary expansion and resorption cavities removed the innermost primary cortex. Scale bar, 1 mm. (B) Enlargement of the area from (A) within the green box, photographed using a full lambda (530 nm) plate to reveal fiber orientation. Two LAGs are visible in this region (arrows), partially destroyed by medullary expansion. Tissue within the cortex is lamellar to somewhat parallel-fibered and vascular canals are longitudinal. Scale bar, 100 µm.

10.7717/peerj.422/supp-21 Figure S19 Osteohistology of the coracoid blade of alligator MOR-OST 1648 (A) Transverse section. Due to the size of the medullary cavity, it is likely some CGMs were lost to resorption. Scale bar, 1 mm. (B) Enlargement of the area from (A) within the green box, photographed using a full lambda (530 nm) plate to reveal fiber orientation. The lower half of the image contains a strut of remodeled bone within the medullary cavity, and the upper half shows the largely avascular inner cortex. Scale bar, 200 µm.

10.7717/peerj.422/supp-22 Figure S20 Osteohistology of the coracoid blade of alligator MOR-OST 1649 (A) Transverse section. The innermost annulus (arrow) is partially destroyed by expansion of the large medullary cavity. Scale bar, 1 mm. (B) Enlargement of the area from (A) within the green box, photographed using a full lambda (530 nm) plate to reveal fiber orientation. Tissue in the region is avascular and lamellar. Periosteal attachment fibers radiate outward within the cortex. Scale bar, 500 µm.

10.7717/peerj.422/supp-23 Figure S21 Osteohistology of the coracoid blade of alligator MOR-OST 1650 (A) Transverse section. The innermost CGM is nearly destroyed by the large marrow cavity and surrounding resorption cavities. Missing cortex in the bottom of the image is an artifact of preparation. Scale bar, 1 mm. (B) Enlargement of the area from (A) within the green box, photographed using a full lambda (530 nm) plate to reveal fiber orientation. Tissue in this area is lamellar to parallel-fibered. Periosteal attachment fibers (arrows) radiate frequently within the cortex. Scale bar, 200 µm.

10.7717/peerj.422/supp-24 Figure S22 Osteohistology of the scapula blade of alligator MOR-OST 1648 (A) Transverse section. Scale bar, 1 mm. (B) Enlargement of the area from (A) within the green box, photographed using a full lambda (530 nm) plate to reveal fiber orientation. The lamellar cortex contains scattered vascular canals, but the mid cortex is largely avascular. Scale bar, 500 µm.

10.7717/peerj.422/supp-25 Figure S23 Osteohistology of the scapula blade of alligator MOR-OST 1649 (A) Transverse section. Medullary expansion has partially destroyed several CGMs within the cortex. Scale bar, 1 mm. (B) Enlargement of the area from (A) within the green box, photographed using a full lambda (530 nm) plate to reveal fiber orientation. This region is predominately avascular and lamellar with scattered osteocytes. Five annuli followed by LAGs (arrows) appear as lighter bands within the cortex. Scale bar, 500 µm.

10.7717/peerj.422/supp-26 Figure 24 Osteohistology of the scapula blade of alligator MOR-OST 1650 (A) Transverse section. In several areas CGMs are partially destroyed by large resorption cavities. Scale bar, 1 mm. (B) Enlargement of the area from (A) within the green box, photographed using a full lambda (530 nm) plate to reveal fiber orientation. Near the center of the image, remodeled secondary tissue borders the medullary cavity, and resorption cavities are present in the lower half. Primary tissue in this area is fairly avascular and lamellar. Four annuli followed by LAGs (arrows) appear as light bands within the cortex. Scale bar, 500 µm.

10.7717/peerj.422/supp-27 Figure S25 Osteohistology of a nuchal osteoderm from alligator MOR-OST 1648 (A) Transverse section, stained with Toluidine chloride. Scale bar, 1 mm. (B) Enlargement of the area from (A) within the green box, photographed using a full lambda (530 nm) plate to reveal fiber orientation. Tissue is highly disorganized and richly vascularized. The vascular canals appear transparent and do not have a common orientation. Periosteal attachment fibers are also frequent and disorganized. Scale bar, 500 µm.

10.7717/peerj.422/supp-28 Figure 26 Osteohistology of a nuchal osteoderm from alligator MOR-OST 1649 (A) Transverse section, stained with Toluidine chloride. Three to four CGMs are observed near the ventral surface at low magnification. Scale bar, 1 mm. (B) Enlargement of the area from (A) within the green box, photographed using a full lambda (530 nm) plate to reveal fiber orientation. Two dense lines of periosteal attachment fibers may indicate the location of CGMs. These fibers are also frequent and disorganized within the tissue matrix. Scale bar, 500 µm.

10.7717/peerj.422/supp-29 Figure S27 Osteohistology of a nuchal osteoderm from alligator MOR-OST 1650 (A) Transverse section, stained with Toluidine chloride. One CGM is observed near the ventral surface at low magnification, seeming to outline the shape of the osteoderm earlier in ontogeny. Scale bar, 1 mm. (B) Enlargement of the area from (A) within the green box, photographed using a full lambda (530 nm) plate to reveal fiber orientation. The region near the apex of the keel is richly vascularized and bone is cancellous. Scale bar, 500 µm.

## Additional Information and Declarations

### Data Deposition

The following information was supplied regarding the deposition of related data: MorphoBank project number P731.

## References

[ref-1] Bourdon E, Castanet J, Ricqlès Ad, Scofield P, Tennyson A, Lamrous H, Cubo J (2009). Bone growth marks reveal protracted growth in New Zealand kiwi (Aves, Apterygidae). Biology Letters.

[ref-2] Caetano MH, Castanet J (1993). Variability and microevolutionary patterns in *Triturus marmoratus* from Portugal: age, size, longevity and individual growth. Amphibia-Reptilia.

[ref-3] Castanet J, Croci S, Aujard F, Perret M, Cubo J, Margerie Ed (2004). Lines of arrested growth in bone and age estimation in a small primate: *Microcebus murinus*. Journal of Zoology, London.

[ref-4] Castanet J, Francillon-Vieillot H, Meunier PJ, Ricqlès Ad, Hall BK (1993). Bone and individual aging. Bone.

[ref-5] Castanet J, Grandin A, Abourachid A, de Ricqlès A (1996). Expression de la dynamique de croissance dans la structure de l’os périostique chez *Anas plathyrhynchos*. Comptes Rendus de l’Academie des Sciences Serie III Sciences de la Vie.

[ref-6] Castanet J, Rogers KC, Cubo J, Jacques-Boisard J (2000). Periosteal bone growth rates in extant ratites (ostriche and emu). Implications for assessing growth in dinosaurs. Comptes Rendus de l’Academie des Sciences Serie III Sciences de la Vie.

[ref-7] Chabreck RH, Joanen T (1979). Growth rates of American alligators in Louisiana. Herpetologica.

[ref-8] Chinsamy A (1993). Image analysis and the physiological implications of the vascularisation of femora in archosaurs. Modern Geology.

[ref-9] Chinsamy A, Hillenius WJ, Weishampel DB, Dodson P, Osmólska H (2004). Physiology of nonavian dinosaurs. The dinosauria.

[ref-10] Coulson TD, Coulson RA, Hernandez T (1973). Some observations on the growth of captive alligators. Zoologica.

[ref-11] Cubo J, Le Roy N, Martinez-Maza C, Montes L (2012). Paleohistological estimation of bone growth rate in extinct archosaurs. Paleobiology.

[ref-12] de Buffrenil V (1980). Preliminary data on the structure of growth marks among living and fossil crocodilians. Bulletin de la Socit Zoologique de France.

[ref-13] de Buffrénil V, Castanet J (2000). Age estimation by skeletochronology in the Nile monitor (*Varanus niloticus*), a highly exploited species. Journal of Herpetology.

[ref-14] de Ricqlès A, Bellairs AdA, Cox CB (1976). On bone histology of fossil and living reptiles, with comments on its functional and evolutionary significance. Morphology and biology of reptiles.

[ref-15] de Ricqlès A, Thomas RDK, Olson EC (1980). Tissue structures of dinosaur bone: functional significance and possible relation to dinosaur physiology. A cold look at the warm blooded dinosaurs.

[ref-16] de Ricqlès A (1983). Cyclical growth in the long limb bones of a sauropod dinosaur. Acta Palaeontologica Polonica.

[ref-17] de Ricqlès A, Padian K, Horner JR, Gauthier J, Gall LF (2001). The bone histology of basal birds in phylogenetic and ontogenetic perspectives. New perspectives on the origin and early evolution of birds.

[ref-18] Doube M, Kłosowski MM, Arganda-Carreras I, Cordelières F, Dougherty RP, Jackson J, Schmid B, Hutchinson JR, Shefelbine SJ (2010). BoneJ: free and extensible bone image analysis in ImageJ. Bone.

[ref-19] Farlow JO, Hayashi S, Tattersall GJ (2010). Internal vascularity of the dermal plates of *Stegosaurus* (Ornithischia, Thyreophora). Swiss Journal of Geoscience.

[ref-20] Farmer CG, Sanders K (2010). Unidirectional airflow in the lungs of alligators. Science.

[ref-21] Francillon-Vieillot H, de Buffrenil V, Castanet J, Geraudie J, Meunier FJ, Sire JY, Zylberberg L, de Ricqlès A, Carter JG (1990). Microstructure and mineralization of vertebrate skeletal tissues. Skeletal biomineralization patterns, processes and evolutionary trends.

[ref-22] Frey TvE (1988). Das Tragsystem der Krododile- eine biomechanische und phylogenetische Analyse. Stuttgarter Beiträge zur Naturkunde Serie A (Biologie).

[ref-23] Frylestam B, von Schantz T (1977). Age determination of European hares based on periosteal growth lines. Mammal Review.

[ref-24] Griffiths I (1961). Skeletal lamellae as an index of age in Heterothermous Tetrapods. Annals and Magazine of Natural History: Series 13.

[ref-25] Hall BK (2005). Bones and cartilage: developmental and evolutionary skeletal biology.

[ref-26] Ham AW, Cormack DH (1979). Histophysiology of cartilage, bone and joints.

[ref-27] Hemelaar ASM, van Gelder JJ (1980). Annual growth rings in phalanges of *Bufo bufo* (Anura, Amphibia) from the Netherlands and their use for age determination. Netherlands Journal of Zoology.

[ref-28] Horner JR, de Ricqlès A, Padian K (1999). Variation in dinosaur skeletochronology indicators: implications for age assessment and physiology. Paleobiology.

[ref-29] Horner JR, de Ricqlès A, Padian K (2000). Long bone histology of the hadrosaurid dinosaur *Maiasaura peeblesorum*: growth dynamics and physiology based on an ontogenetic series of skeletal elements. Journal of Vertebrate Paleontology.

[ref-30] Horner JR, Padian K, Ricqlès Ad (2001). Comparative osteohistology of some embryonic and perinatal archosaours: developmental and behavioral implications for dinosaurs. Paleobiology.

[ref-31] Hutton JM (1986). Age determination of living Nile crocodiles from the cortical stratification of bone. Copeia.

[ref-32] Joanen T, McNease L, Grahame JW, Webb S, Manolis C, Whitehead PJ (1987). Alligator farming research in Louisiana, USA. Wildlife management: crocodiles and alligators.

[ref-33] Klein N (2010). Long bone histology of sauropterygia from the lower muschelkalk of the Germanic Basin provides unexpected implications for phylogeny. PLoS ONE.

[ref-34] Klein N, Scheyer T, Tütken T (2009). Skeletochronology and isotopic analysis of a captive individual of *Alligator mississippiensis* Daudin, 1802. Fossil Record.

[ref-35] Köhler M, Marin-Moratalla N, Jordana X, Aanes R (2012). Seasonal bone growth and physiology in endotherms shed light on dinosaur physiology. Nature.

[ref-36] Konietzkoo-Meier D, Klein N (2013). Unique growth pattern of Metoposaurus diagnosticus krasiejowensis (Amphibia, Temnospondyli) from the Upper Triassic of Krasiejów, Poland. Palaeogeography, Palaeoclimatology, Palaeoecology.

[ref-37] Lee AH (2004). Histological organization and its relationship to function in the femur of *Alligator mississippiensis*. Journal of Anatomy.

[ref-38] Legendre LJ, Segalen L, Cubo J (2013). Evidence for high bone growth rate in Euparkeria obtained using a new paleohistological inference model for the humerus. Journal of Vertebrate Paleontology.

[ref-39] O’Leary MA, Kaufman SG (2012). http://www.morphobank.org.

[ref-40] Padian K, Horner JR, Weishampel DB, Dodson P, Osmólska H (2004). Dinosaur physiology. The dinosauria.

[ref-41] Padian K, Horner JR, Ricqlès Ad (2004). Growth in small dinosaurs and pterosaurs: the evolution of archosaurian growth strategies. Journal of Vertebrate Paleontology.

[ref-42] Padian K, Ricqlès Ad, Horner JR (2001). Dinosaurian growth rates and bird origins. Nature.

[ref-43] Peabody FE (1961). Annual growth zones in living and fossil vertebrates. Journal of Morphology.

[ref-44] Rasband WS (1997–2012). http://imagej.nih.gov/ij/.

[ref-45] Reid RH (1984). Primary bone and dinosaurian physiology. Geological Magazine.

[ref-46] Reid RH (1987). Bone and dinosaurian “endothermy”. Modern Geology.

[ref-47] Reid RH (1990). Zonal “growth rings” in dinosaurs. Modern Geology.

[ref-48] Reid RH, Farlow JO, Brett-Surman MK (1997). How dinosaurs grew. The complete dinosaur.

[ref-49] Roberts ED, Matlock CL, Joanen T, McNease L, Bowen M (1988). Bone morphometrics and tetracycline marking patterns in young growing American alligators. Journal of Wildlife Diseases.

[ref-50] Sander PM, Andrássy P (2006). Lines of arrested growth and long bone histology in Pleistocene large mammals from Germany: what do they tell us about dinosaur physiology?. Palaeontographica A.

[ref-51] Schweitzer MH, Elsey RM, Dacke CG, Horner JR, Lamm E-T (2007). Do egg-laying crocodilian (*Alligator mississippiensis*) archosaurs form medullary bone?. Bone.

[ref-52] Starck JM, Chinsamy A (2002). Bone microstructure and developmental plasticity in birds and other dinosaurs. Journal of Morphology.

[ref-53] Stein K, Prondvai E (2014). Rethinking the nature of fibrolamellar bone: an integrative biological revision of sauropod plexiform bone formation. Biological Reviews of the Cambridge Philosophical Society.

[ref-54] Tucker AD (1997). Validation of skeletochronology to determine age of freshwater crocodiles (*Crocodylus johnstoni*). Australian Journal of Marine and Freshwater Research.

[ref-55] Tumarkin-Deratzian AR (2007). Fibrolamellar bone in wild adult *Alligator mississippiensis*. Journal of Herpetology.

[ref-56] Turvey ST, Green OR, Holdaway R (2005). Cortical growth marks reveal extended juvenile development in New Zealand moa. Nature.

[ref-57] Waye HL, Gregory PT (1998). Determining the age of garter snakes (*Thamnophis spp.*) by means of skeletochronology. Canadian Journal of Zoology.

[ref-58] Werning S (2012). The ontogenetic osteohistology of *Tenontosaurus tilletti*. PLoS ONE.

