# Supplementary material for: Quantification of intraskeletal histovariability in Alligator mississippiensis and implications for vertebrate osteohistology
Source: PeerJ. 2014 Jun 3;2:e422. doi: 10.7717/peerj.422 (PMC4060058; doi:10.7717/peerj.422)
Supplement: Supplemental Information 1 [file peerj-02-422-s001.docx]

Humerus

MOR-OST 1648

In the left humerus (Fig. S1), three CGMs are visible but only the outermost is fully traceable. The innermost annulus was partially eroded by medullary expansion and the second annulus is too faint to accurately trace. Medullary expansion in the right humerus also partially obliterated the presence of the innermost growth mark. The outermost growth mark on both humerii consists of an annulus terminating in a LAG and is easily traced. The innermost cortex is vascularized by longitudinal and radially anastomosing canals. These are found within a mineralized matrix of woven fibers. Vascular canal density is sharply reduced in the mid cortex near the second annulus, and bone fibers are parallel. The outer cortex is also parallel fibered but resembles the inner cortex in vascular canal density.

MOR-OST 1649

Small resorption cavities are scattered within the inner cortex (Fig. S2) indicating active medullary expansion at the time of death. Cortical bone tissue consists of mineralized fibers arranged in parallel. Longitudinal simple primary canals dominate the sample, but anastomosing canals are also common beyond the inner cortex, especially approaching the periosteal surface. Within the cortex five annuli are present, but the innermost cannot be fully traced. The outermost annulus is very close to the periosteal surface and is easily overlooked, but can be fully traced in the left humerus.

MOR-OST 1650

The cortex is entirely parallel fibered (Fig. S3), although close to the periosteal surface the fibers are more loosely parallel. Resorption cavities are scattered within the innermost cortex. Five annuli are present. The third and fourth annuli are closely spaced and the fifth is so close to the periosteal surface that it is difficult to fully trace. Vascular canal density is uniform throughout, consisting largely of longitudinal simple primary canals prior to the first annulus and of longitudinal canals often connected by radial anastomoses thereafter.

Radius

MOR-OST 1648

The cortical matrix is lamellar (Fig. S4). There is a concentration of longitudinal simple primary canals in the inner cortex. Mid cortex vascularity is made of sparsely scattered longitudinal simple primary canals, and the outermost cortex is nearly avascular. Three CGMs are visible in the cortex: one partially resorbed innermost LAG, an annulus in the mid cortex, and an annulus followed by a LAG in the outer cortex.

MOR-OST 1649

Most of the cortex is avascular (Fig. S5) save for sparsely scattered longitudinal simple primary canals, and the cortical matrix is completely lamellar. The endosteal layer is well developed. Four annuli are visible.

MOR-OST 1650

The cortex consists of highly organized lamellar tissue (Fig. S6). The endosteal layer is thick and portions of the innermost cortex are composed of secondarily remodeled tissue indicating a previously larger medullary cavity that has since been filled in. Vascular canals are sparse but when present are longitudinal simple primary canals. There are five CGMs within the cortex: three inner annuli, a fourth annulus followed by a LAG, and a fifth annulus in the external cortex. CGMs three and four are closely spaced.

Ulna

MOR-OST 1648

A LAG is visible in the inner cortex (Fig. S7) but partially lost to medullary expansion. Two LAGs within the mid cortex are easily traced. The cortex is made of highly organized, lamellar fibers except in a region of the inner to mid cortex prior to the second LAG. Here longitudinal primary osteons are found within woven tissue and osteocyte density is high. Elsewhere longitudinal simple primary canals and small primary osteons are sparsely scattered throughout the cortex.

MOR-OST 1649

The medullary cavity is surrounded by a thick endosteal layer, which cuts across a considerable amount of primary as well as secondary tissue in the inner cortex (Fig. S8). The dense concentration of secondary tissue on one side of the medullary cavity suggests that the medullary cavity drifted, prompting infilling via secondary tissue. Within the cortex vascular canals are sparse but concentrate to one side of the cortex and consist of longitudinal primary osteons. Primary tissue is weakly lamellar throughout. There are two LAGs within the cortex and an annulus at the outermost cortex, very close to the periosteal surface. Because of the extent of secondary remodeling in the inner cortex it is likely that inner cortical LAGs are missing.

MOR-OST 1650

The medullary cavity is surrounded by an endosteal layer that cuts across a considerable amount of secondary tissue (Fig. S9) indicating a shift in the position of the medullary cavity with respect to the cortical bone. There is a fairly even scattering of longitudinal simple primary canals and primary osteons from inner to outer cortex. There are three annuli followed by LAGs within the cortex and an annulus bordering the periosteal surface. Two of the CMGs are closely spaced in the mid cortex. Due to the large amount of medullary drift and reconstruction, CGMs may be missing within the innermost cortex. Tissue is predominately lamellar.

Femur

MOR-OST 1648

The inner to mid cortex is well vascularized by longitudinal simple primary canals connected by radial anastomoses (Fig. S10). Vascular canal density is low mid cortex but is high again near the periosteal surface. A partial annulus is present near the medullary cavity. An annulus and a LAG are within the mid cortex. The tissue between the endosteal layer and the innermost partial annulus is parallel fibered, but following the LAG vascular canal density is increased and the tissue is woven until the second CGM. Thereafter, fibers are again parallel and the tissue is less well vascularized. After the LAG vascular canal density is higher but fibers remain parallel.

MOR-OST 1649

The inner cortex bounding the medullary cavity was being resorbed at the time of termination (Fig. S11) and the entire cortex consists of loosely parallel fibers. Vascular canal density is fairly consistent throughout the section, made predominately of longitudinal simple primary canals but with some connecting radial anastomoses. Three annuli are observed within the cortex. There is also an annulus very close to the periosteal surface on the left femur, but it cannot be traced completely. Due to the size of the medullary cavity with respect to the circumference of a hatchling femur, it is likely that some CGMs were destroyed by medullary expansion.

MOR-OST 1650

Active medullary expansion was occurring in the femur at the time of termination as indicated by large, scalloped resorption cavities within the inner cortex surrounding the medullary cavity (Fig. S12). Evidence of medullary drift is revealed by a large concentration of secondary tissue to one side of the medullary cavity. A partial annulus is still visible within the innermost cortex, and there are three more annuli within the cortex. Tissue is organized loosely in parallel or is woven in places. The cortex is vascularized largely by longitudinal simply primary canals and longitudinal primary osteons, but there are also some radiating vascular canals. As with other elements of MOR-OST 1650, the third and fourth growth marks are closely spaced.

Tibia

MOR-OST 1648

There are three CGMs (Fig. S13), but the innermost annulus is all but destroyed. The second growth mark is an annulus, and the third growth mark is an annulus followed by a LAG. The innermost cortex is woven and vascularized by both longitudinal and anastomosing vascular canals. After the second growth mark vascular density is lower in the mid cortex and the fiber orientation is parallel. Although vascular density is high again in the outer cortex, fiber arrangement remains parallel and the vascular canals are largely longitudinal simple primary canals.

MOR-OST 1649

Large resorption cavities with only thin lamellar linings indicate recent resorption and medullary enlargement (Fig. S14). Fibers are organized in parallel throughout the cortex with evenly distributed longitudinal to somewhat radial simple primary canals. Five annuli are present. The innermost annulus is almost fully destroyed and cannot be traced. The second annulus is also partially destroyed by medullary expansion, but enough is present that the circumference can be reasonably estimated. The fifth annulus is very close to the periosteal surface and sometimes appears to merge with it.

MOR-OST 1650

Four annuli are visible (Fig. S15) and the innermost annulus is located very close to the medullary cavity. Tissue is woven between the inner cortex and the second annulus in some areas and loosely parallel from the second to the fourth annulus. Between the fourth annulus and the periosteal surface the tissue is largely loosely parallel but does become woven in areas. Annuli 3 and 4 are closer to each other than either is to another CGM. Vascular density is consistent throughout the cortex and is a combination of longitudinal and anastomosing primary osteons and simple primary canals.

Fibula

MOR-OST 1648

Three CGMs are observed within the cortex (Fig. S16) represented by an innermost annulus partially destroyed by resorption, an annulus in the mid cortex, and a LAG near the outer cortex. The innermost cortex prior to the first LAG is avascular, and formed from lamellar tissue. Between the first and second LAG, tissue is woven in areas with longitudinal vascular canals but is lamellar again from the mid cortex to the surface. Sharpey’s fibers are densely scattered throughout the section.

MOR-OST 1649

To one side the mid cortex is sparsely vascularized by longitudinal simple primary canals and nearly avascular on the opposite side (Fig. S17). Tissue consists of highly organized, lamellar fibers throughout. Five LAGs are visible, but the innermost is not fully traceable. The outer-most LAG is very close to the periosteal surface and often merges with it. Sharpey’s fibers are frequent.

MOR-OST 1650

The cortex is largely lamellar but with some parallel fibered tissue, and contains sparse longitudinal simple primary canals (Fig. S18). Four annuli are visible in the cortex, including a closely spaced third and fourth annulus. A fifth annulus is very close to the periosteal surface, often merging with it.

Coracoid

MOR-OST 1648

There are struts of secondary bone within the medullary cavity (Fig. S19). Both the trabecular struts as well as the medullary cavity are lined by lamellar tissue. The entire cortex is lamellar with scattered longitudinal vascular canals. Two annuli are visible and it is very likely inner growth marks were destroyed by medullary expansion.

MOR-OST 1649

The medullary cavity and the trabecular struts within it are lined by endosteal bone (Fig. S20). The lamellar cortex contains longitudinal and radial simple primary canals. Four annuli are visible, although the innermost is partially destroyed by medullary expansion. The fourth annulus is faint in areas.

MOR-OST 1650

Struts of remodeled bony trabeculae are present within the medullary cavity, lined by lamellar endosteal bone (Fig. S21). Scattered resorption cavities are present in the innermost cortex and tissue ranges from lamellar to loosely parallel fibered throughout the cortex. Vascular canals are longitudinal simple primary canals as well as primary osteons. Four LAGs are clearly visible, but the innermost is nearly completely resorbed. Two LAGs in the mid cortex are closely spaced, as in other elements of MOR-OST 1650.

Scapula

MOR-OST 1648

The left and right scapulae were not sectioned at approximately the same location, so the overall shape of the cross sections are different. Despite this, the same number of growth marks is found in both scapulae. A partial LAG remains near the medullary cavity and two annuli are observed within the mid cortex. The entire cortex is made of largely avascular lamellar tissue, although nearer the medullary cavity in places vascularity consists of longitudinal canals (Fig. S22).

MOR-OST 1649

Medullary expansion was active at the time of termination, evident by the large resorption cavities present and the scalloped border of the medullary cavity (Fig. S23). Tissue within the cortex ranges from lamellar to loosely parallel fibered. As with MOR-OST 1648, the right and left scapulae were not sectioned at approximately the same region, resulting in different shapes for cross sections. However, there are five annuli in both scapulae and the innermost CGM is nearly destroyed by medullary expansion. The fifth annulus is so close to the periosteal surface that it often merges with it. Vascular canals are longitudinal and radial simple primary canals and primary osteons.

MOR-OST 1650

There are large resorption cavities and patches of secondary remodeling within the innermost cortex (Fig. S24). Tissue is lamellar to loosely parallel fibered with longitudinal simple primary canals and primary osteons. Four annuli followed by LAGs are present. The inner two growth marks are partially destroyed by medullary drift. The third and fourth CGMs are fairly closely spaced.

Osteoderms

For each alligator it was very difficult to count CGMs within the osteoderms, and impossible to fully trace their extent (Figs. S25-S27). As Tucker ([1997](#_ENREF_59)) observed, remodeling was reduced on the ventral surface, and therefore should preserve the most CGMs compared with other surfaces. The smallest osteoderms are from MOR-OST 1648 (Fig. S25), and they are the most symmetrical in transverse section. In general, tissue within osteoderms is woven, but more parallel fibered near the exterior. Osteoderms are richly vascularized, with vascular canal density highest in the center and in patches near the surface. Approaching the keel, bone density decreases and osteoderms are more porous. Sharpey’s fibers are frequent and often dense throughout the base of the osteoderm which may obscure some CGMs.

Supplemental Figures

Figure S1. Osteohistology of the mid-diaphyseal humerus of alligator MOR-OST 1648. A) Transverse section. Vascular canal density is low in the mid cortex and higher towards the periosteal surface. The arrow indicates Sharpey’s fibers running parallel to an annulus. Scale bar, 1 mm. B) Enlargement of the area from A) within the green box, photographed using a full lambda (530 nm) plate to reveal fiber orientation, but which also tends to obscure growth marks. The endosteal layer (green arrow) cuts across the well vascularized and woven primary tissue of the inner cortex. The mid cortex is less well vascularized and is parallel fibered, while the outer cortex is again well vascularized but remains parallel fibered. Sharpey’s fibers (white arrow) appear as wavy black lines and are here arranged perpendicular to bone tissue orientation. Scale bar, 500 µm.

Figure S2. Osteohistology of the mid-diaphyseal humerus of alligator MOR-OST 1649. A) Transverse section. Scale bar, 1 mm. B) Enlargement of the area from A) within the green box, photographed using a full lambda (530 nm) plate to reveal fiber orientation. Three annuli are in view (green arrows). Resorption cavities (white arrow) are present near the medullary cavity, and tissue is parallel fibered. Vascular canal density is uniform throughout the cortex, consisting of longitudinal simple primary canals as well as obliquely anastomosing canals. Scale bar, 500 µm.

Figure S3. Osteohistology of the mid-diaphyseal humerus of alligator MOR-OST 1650. A) Transverse section. Scale bar, 1 mm. B) Enlargement of the area from A) within the green box, photographed using a full lambda (530 nm) plate to reveal fiber orientation, but which also tends to obscure growth marks. Regardless, three annuli (arrows) are visible in this portion of the cortex. The cortex is parallel fibered throughout, and vascular density is uniform. Vascular orientation is mostly longitudinal simple primary canals within the inner cortex, becoming predominately obliquely anastomosing by mid cortex. Scale bar, 500 µm.

Figure S4. Osteohistology of the mid-diaphyseal radius of alligator MOR-OST 1648. A) Transverse section. Scale bar, 1 mm. B) Enlargement of the area from A) within the green box, photographed using a full lambda (530 nm) plate to reveal fiber orientation. Two growth marks are visible in this enlargement (arrows). The cortex is of highly organized lamellar tissue with a sparse scattering of longitudinal simple primary canals. Scale bar, 100 µm.

Figure S5. Osteohistology of the mid-diaphyseal radius of alligator MOR-OST 1649. A) Transverse section. Scale bar, 1 mm. B) Enlargement of the area from A) within the green box, photographed using a full lambda (530 nm) plate to reveal fiber orientation. The cortex is of highly organized lamellar tissue and is nearly avascular. Green arrows point to four CGMs in this region. A well-developed endosteal layer (left) cuts across primary tissue and in places consists of secondary osteons, indicating medullary drift. Scale bar, 500 µm.

Figure S6. Osteohistology of the mid-diaphyseal radius of alligator MOR-OST 1650. A) Transverse section. The well-developed endosteal layer cuts across primary tissue and in some areas is replaced by secondary osteons, indicating medullary drift. Scale bar, 1 mm. B) Enlargement of the area from A) within the green box, photographed using a full lambda (530 nm) plate to reveal fiber orientation. The cortex is made of highly organized lamellar tissue containing scattered longitudinal simple primary canals and primary osteons. Three CGMs (arrows) are evident in the enlargement. Scale bar, 100 µm.

Figure S7. Osteohistology of the mid-diaphyseal ulna of alligator MOR-OST 1648. A) Transverse section. Sharpey’s fibers (arrow) arranged parallel to a CGM are evident even at low magnification. Scale bar, 1 mm. B) Enlargement of the area from A) within the green box, photographed using a full lambda (530 nm) plate to reveal fiber orientation. Here the cortex is lamellar, with sparsely scattered longitudinal simple primary canals. The innermost LAG is partially destroyed by medullary expansion and two more are visible within the cortex (green arrows). Scale bar, 100 µm.

Figure S8. Osteohistology of the mid-diaphyseal ulna of alligator MOR-OST 1649. A) Transverse section. The high degree of endosteal remodeling is apparent even at low magnification, especially in the region highlighted within the green box. Scale bar, 1 mm. B) Enlargement of the area from A) within the green box, photographed using a full lambda (530 nm) plate to reveal fiber orientation. Secondary reconstruction occurred within the inner cortex due to medullary drift, resulting in large, overlapping secondary osteons. Scale bar, 100 µm.

Figure S9. Osteohistology of the mid-diaphyseal ulna of alligator MOR-OST 1650. A) Transverse section. Scale bar, 1 mm. B) Enlargement of the area from A) within the green box, photographed using a full lambda (530 nm) plate to reveal fiber orientation. Two annuli followed by LAGs are marked by green arrows. Medullary expansion was active in this region as indicated by the partially resorbed endosteal layer (white arrow) and resorption cavity within the inner cortex. Tissue organization within the cortex is lamellar, and vascularity is longitudinal. Scale bar, 500 µm.

Figure S10. Osteohistology of the mid-diaphyseal femur of alligator MOR-OST 1648. A) Transverse section shows a combination of anastomosing and radial vascular canals. Scale bar, 1 mm. B) Enlargement of the area from A) within the green box, photographed using a full lambda (530 nm) plate to reveal fiber orientation, but which also tends to obscure growth marks. Tissue within the innermost cortex is woven but is parallel fibered by mid cortex. Scale bar, 500 µm.

Figure S11. Osteohistology of the mid-diaphyseal femur of alligator MOR-OST 1649. A) Transverse section, stained with Toluidine chloride. Scale bar, 1 mm. B) Enlargement of the area from A) within the green box, photographed using a full lambda (530 nm) plate to reveal fiber orientation. The cortical tissue is loosely parallel fibered. Scale bar, 500 µm.

Figure S12. Osteohistology of the mid-diaphyseal femur of alligator MOR-OST 1650. A) Transverse section photographed using circularly polarized light. Medullary drift is evident by the concentration of secondary tissue endosteally, and active medullary expansion is revealed by large resorption cavities within the inner cortex. Scale bar, 1 mm. B) Enlargement of the area from A) within the green box, photographed using a full lambda (530 nm) plate to reveal fiber orientation. The cortical tissue is loosely parallel fibered. Scale bar, 500 µm.

Figure S13. Osteohistology of the mid-diaphyseal tibia of alligator MOR-OST 1648. A) Transverse section. Scale bar, 1 mm. B) Enlargement of the area from A) within the green box, photographed using a full lambda (530 nm) plate to reveal fiber orientation. Fibers are woven within the inner cortex but are parallel fibered by mid cortex. Vascular canals are obliquely anastomosing. Scale bar, 500 µm.

Figure S14. Osteohistology of the mid-diaphyseal tibia of alligator MOR-OST 1649. A) Transverse section, stained with Toluidine chloride. Scale bar, 1 mm. B) Enlargement of the area from A) within the green box, photographed using a full lambda (530 nm) plate to reveal fiber orientation. Medullary expansion is evident by the resorbed region of inner cortex (white arrow) and the partial destruction of the innermost annulus (green arrow). Two more annuli (green arrows) are present within the cortex. Tissue is parallel fibered and most vascular canals are either longitudinal simple primary or somewhat anastomosing. Scale bar, 500 µm.

Figure S15. Osteohistology of the mid-diaphyseal tibia of alligator MOR-OST 1650. A) Transverse section. Scale bar, 1 mm. B) Enlargement of the area from A) within the green box, photographed using a full lambda (530 nm) plate to reveal fiber orientation, but which also tends to obscure growth marks. Here, tissue fibers are arranged loosely in parallel. Vascular canals are longitudinal and anastomosing. Scale bar, 500 µm.

Figure S16. Osteohistology of the mid-diaphyseal fibula of alligator MOR-OST 1648. A) Transverse section. The innermost cortex is partially destroyed by medullary drift. Scale bar, 1 mm. B) Enlargement of the area from A) within the green box, photographed using a full lambda (530 nm) plate to reveal fiber orientation. This region is largely avascular and the lamellar tissue contains numerous Sharpey’s fibers, visible as radiating, thin wavy black lines. Scale bar, 100 µm.

Figure S17. Osteohistology of the mid-diaphyseal fibula of alligator MOR-OST 1649. A) Transverse section. Scale bar, 1 mm. B) Enlargement of the area from A) within the green box, photographed using a full lambda (530 nm) plate to reveal fiber orientation. Highly organized lamellar fibers contain scattered osteocytes. Sharpey’s fibers are abundant and appear as radiating, thin wavy black lines. Circles on the right of the image are preparation artifacts. Scale bar, 100 µm.

Figure S18. Osteohistology of the mid-diaphyseal fibula of alligator MOR-OST 1650. A) Transverse section. Medullary expansion and resorption cavities removed the innermost primary cortex. Scale bar, 1 mm. B) Enlargement of the area from A) within the green box, photographed using a full lambda (530 nm) plate to reveal fiber orientation. Two LAGs are visible in this region (arrows), partially destroyed by medullary expansion. Tissue within the cortex is lamellar to somewhat parallel fibered and vascular canals are longitudinal. Scale bar, 100 µm.

Figure S19. Osteohistology of the coracoid blade of alligator MOR-OST 1648. A) Transverse section. Due to the size of the medullary cavity, it is likely some CGMs were lost to resorption. Scale bar, 1 mm. B) Enlargement of the area from A) within the green box, photographed using a full lambda (530 nm) plate to reveal fiber orientation. The lower half of the image contains a strut of remodeled lamellar bone within the medullary cavity, and the upper half shows the largely avascular inner cortex. Scale bar, 200 µm.

Figure S20. Osteohistology of the coracoid blade of alligator MOR-OST 1649. A) Transverse section. The innermost annulus (arrow) is partially destroyed by expansion of the large medullary cavity. Scale bar, 1 mm. B) Enlargement of the area from A) within the green box, photographed using a full lambda (530 nm) plate to reveal fiber orientation. Tissue in the region is avascular and lamellar. Sharpey’s fibers radiate outward within the cortex. Scale bar, 500 µm.

Figure S21. Osteohistology of the coracoid blade of alligator MOR-OST 1650. A) Transverse section. The innermost LAG is nearly destroyed by the large marrow cavity and surrounding resorption cavities. Missing cortex in the bottom of the image is an artifact of preparation. Scale bar, 1 mm. B) Enlargement of the area from A) within the green box, photographed using a full lambda (530 nm) plate to reveal fiber orientation. Tissue in this area is lamellar to parallel fibered. Sharpey’s fibers (arrows) radiate frequently within the cortex. Scale bar, 200 µm.

Figure S22. Osteohistology of the scapula blade of alligator MOR-OST 1648. A) Transverse section. Scale bar, 1 mm. B) Enlargement of the area from A) within the green box, photographed using a full lambda (530 nm) plate to reveal fiber orientation. The lamellar cortex contains scattered vascular canals, but the mid cortex is largely avascular. Scale bar, 500 µm.

Figure S23. Osteohistology of the scapula blade of alligator MOR-OST 1649. A) Transverse section. Medullary expansion has partially destroyed several CGMs within the cortex. Scale bar, 1 mm. B) Enlargement of the area from A) within the green box, photographed using a full lambda (530 nm) plate to reveal fiber orientation. This region is predominately avascular and lamellar with scattered osteocytes. Five annuli (arrows) appear as lighter bands within the cortex. Scale bar, 500 µm.

Figure S24. Osteohistology of the scapula blade of alligator MOR-OST 1650. A) Transverse section. In several areas CGMs are partially destroyed by large resorption cavities. Scale bar, 1 mm. B) Enlargement of the area from A) within the green box, photographed using a full lambda (530 nm) plate to reveal fiber orientation. Near the center of the image, remodeled secondary tissue borders the medullary cavity, and resorption cavities are present in the lower half. Primary tissue in this area is fairly avascular and lamellar. Four annuli followed by LAGs (arrows) are present within the cortex. Scale bar, 500 µm.

Figure S25. Osteohistology of a nuchal osteoderm from alligator MOR-OST 1648. A) Transverse section, stained with Toluidine chloride. Scale bar, 1 mm. B) Enlargement of the area from A) within the green box, photographed using a full lambda (530 nm) plate to reveal fiber orientation. Tissue is highly disorganized and richly vascularized. The vascular canals appear transparent and do not have a common orientation. Sharpey’s fibers are also frequent and disorganized. Scale bar, 500 µm.

Figure S26. Osteohistology of a nuchal osteoderm from alligator MOR-OST 1649. A) Transverse section, stained with Toluidine chloride. Three to four CGMs are observed near the ventral surface at low magnification. Scale bar, 1 mm. B) Enlargement of the area from A) within the green box, photographed using a full lambda (530 nm) plate to reveal fiber orientation. Two dense lines of Sharpey’s fibers may indicate the location of CGMs. These fibers are also frequent and disorganized within the tissue matrix. Scale bar, 500 µm.

Figure S27. Osteohistology of a nuchal osteoderm from alligator MOR-OST 1650. A) Transverse section, stained with Toluidine chloride. One CGM is observed near the ventral surface at low magnification, seeming to outline the shape of the osteoderm earlier in ontogeny. Scale bar, 1 mm. B) Enlargement of the area from A) within the green box, photographed using a full lambda (530 nm) plate to reveal fiber orientation. The region near the apex of the keel is richly vascularized and bone is cancellous. Scale bar, 500 µm.
