# Supplementary figures and images for: Quantification of intraskeletal histovariability in Alligator mississippiensis and implications for vertebrate osteohistology
Source: PeerJ. 2014 Jun 3;2:e422. doi: 10.7717/peerj.422 (PMC4060058; doi:10.7717/peerj.422)

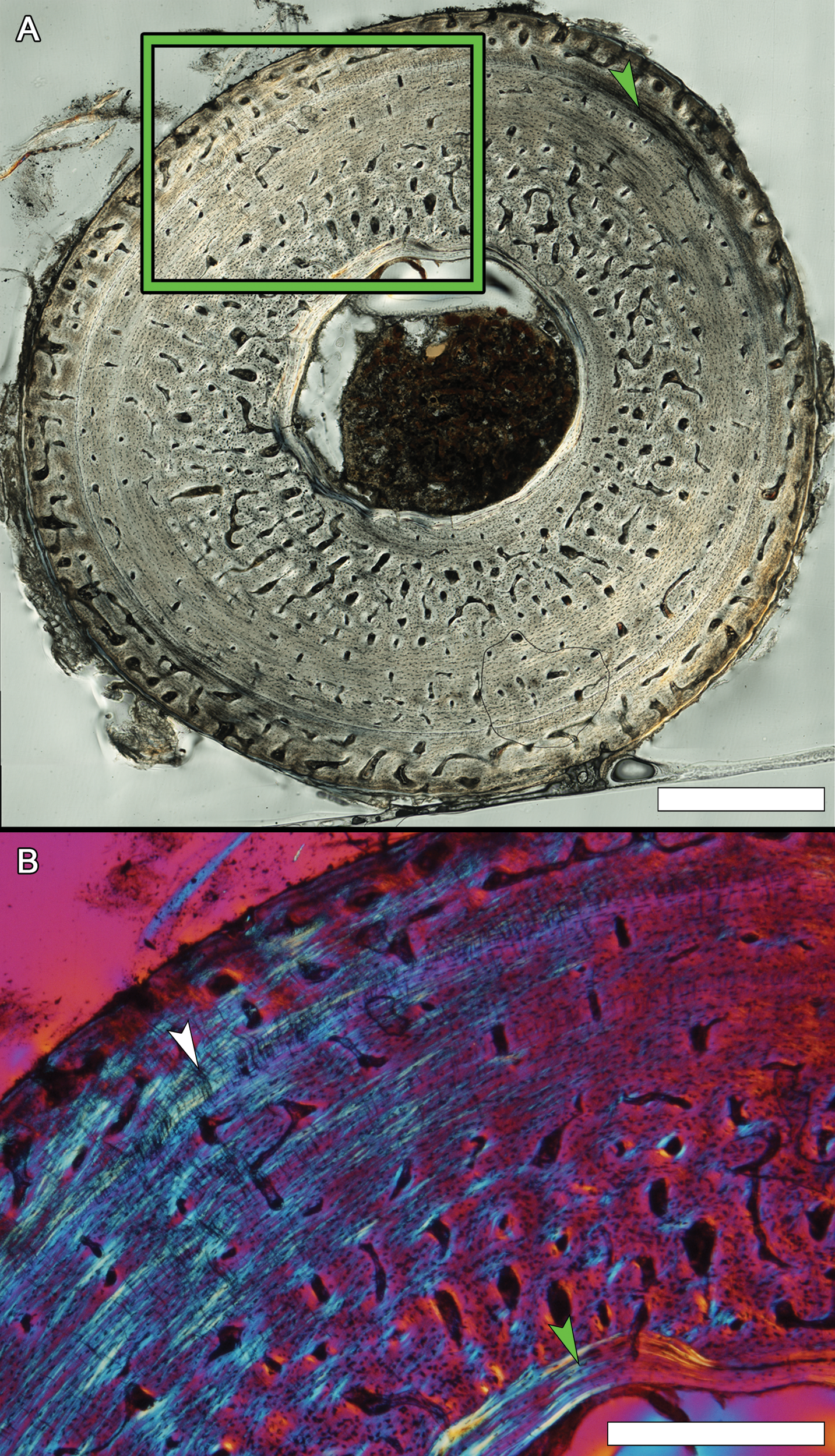

Supplement: Figure S1 — Vascular canal density is low in the mid cortex and higher towards the periosteal surface. The arrow indicates periosteal attachment fibers running parallel to growth mark. Scale bar, 1 mm. (B) Enlargement of the area from (A) within the green box, photographed using a full lambda (530 nm) plate to reveal fiber orientation, but which also tends to obscure growth marks. The endosteal layer (green arrow) cuts across the well vascularized and woven primary tissue of the inner cortex. The mid cortex is less well vascularized and is parallel-fibered, while the outer cortex is again well vascularized but remains parallel-fibered. Periosteal attachment fibers (white arrow) are arranged perpendicular to bone tissue orientation. Scale bar, 500 µm. [file peerj-02-422-s003.png]

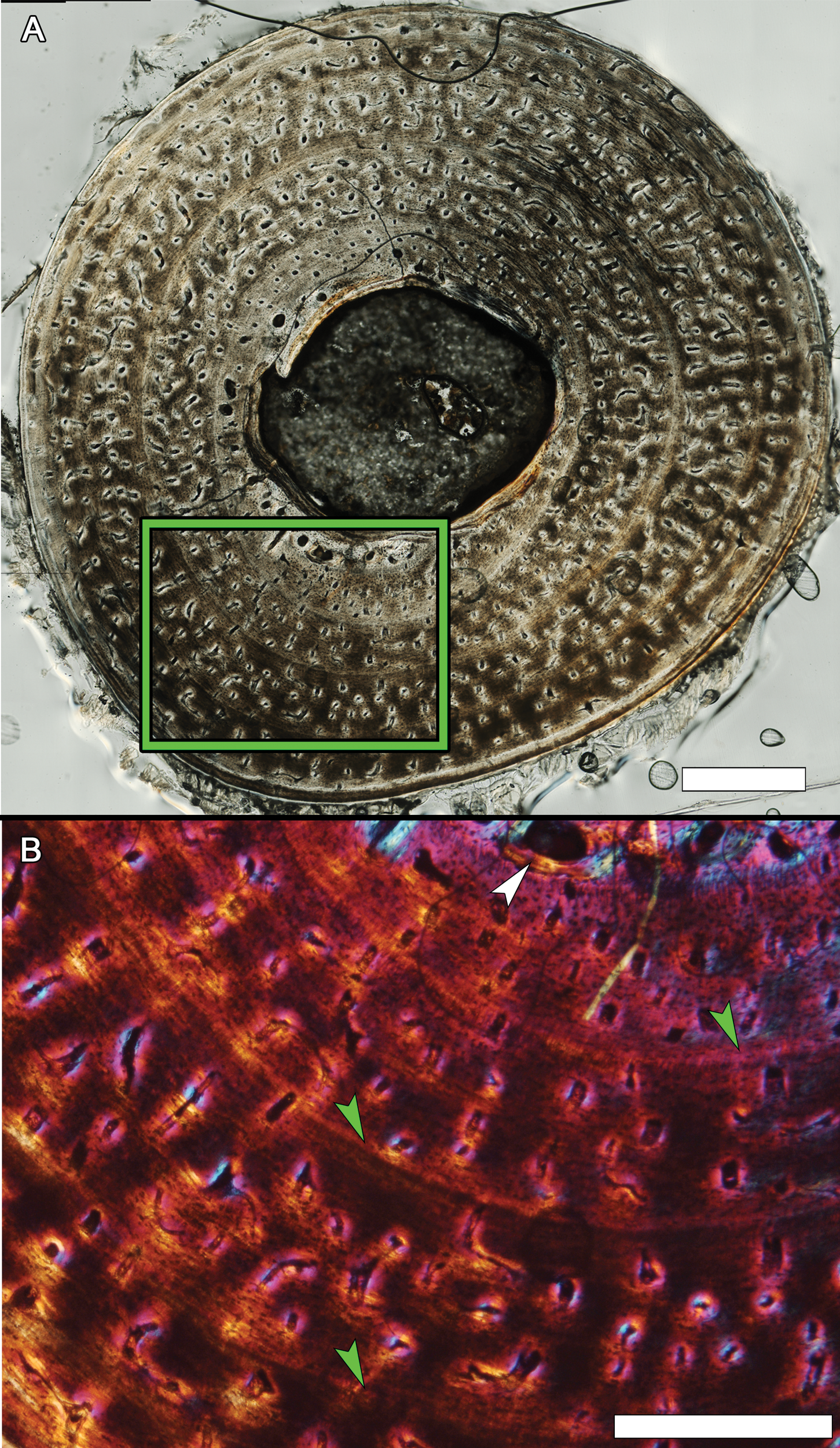

Supplement: Figure S2 — (A) Transverse section. Scale bar, 1 mm. (B) Enlargement of the area from (A) within the green box, photographed using a full lambda (530 nm) plate to reveal fiber orientation. Three growth marks are in view (green arrows). Resorption cavities (white arrow) are present near the medullary cavity, and tissue is parallel-fibered. Vascular canal density is uniform throughout the cortex, consisting of longitudinal as well as obliquely anastomosing canals. Scale bar, 500 µm. [file peerj-02-422-s004.png]

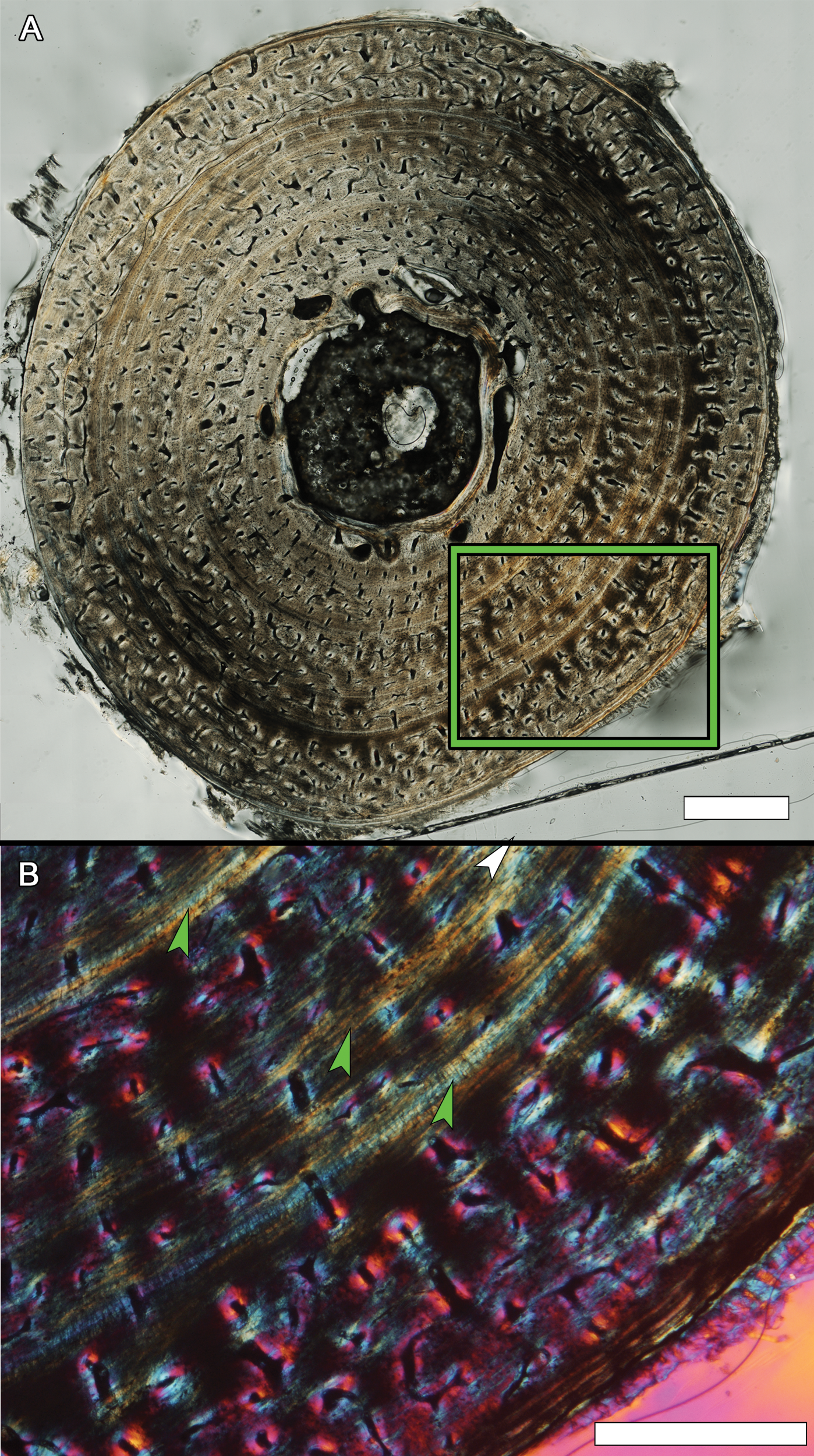

Supplement: Figure S3 — (A) Transverse section. Scale bar, 1 mm. (B) Enlargement of the area from (A) within the green box, photographed using a full lambda (530 nm) plate to reveal fiber orientation, but which also tends to obscure growth marks. Regardless, three growth marks (arrows) are visible in this portion of the cortex. The cortex is parallel-fibered throughout, and vascular density is uniform. Vascular orientation is mostly longitudinal within the inner cortex, becoming predominately obliquely anastomosing by mid cortex. Scale bar, 500 µm. [file peerj-02-422-s005.png]

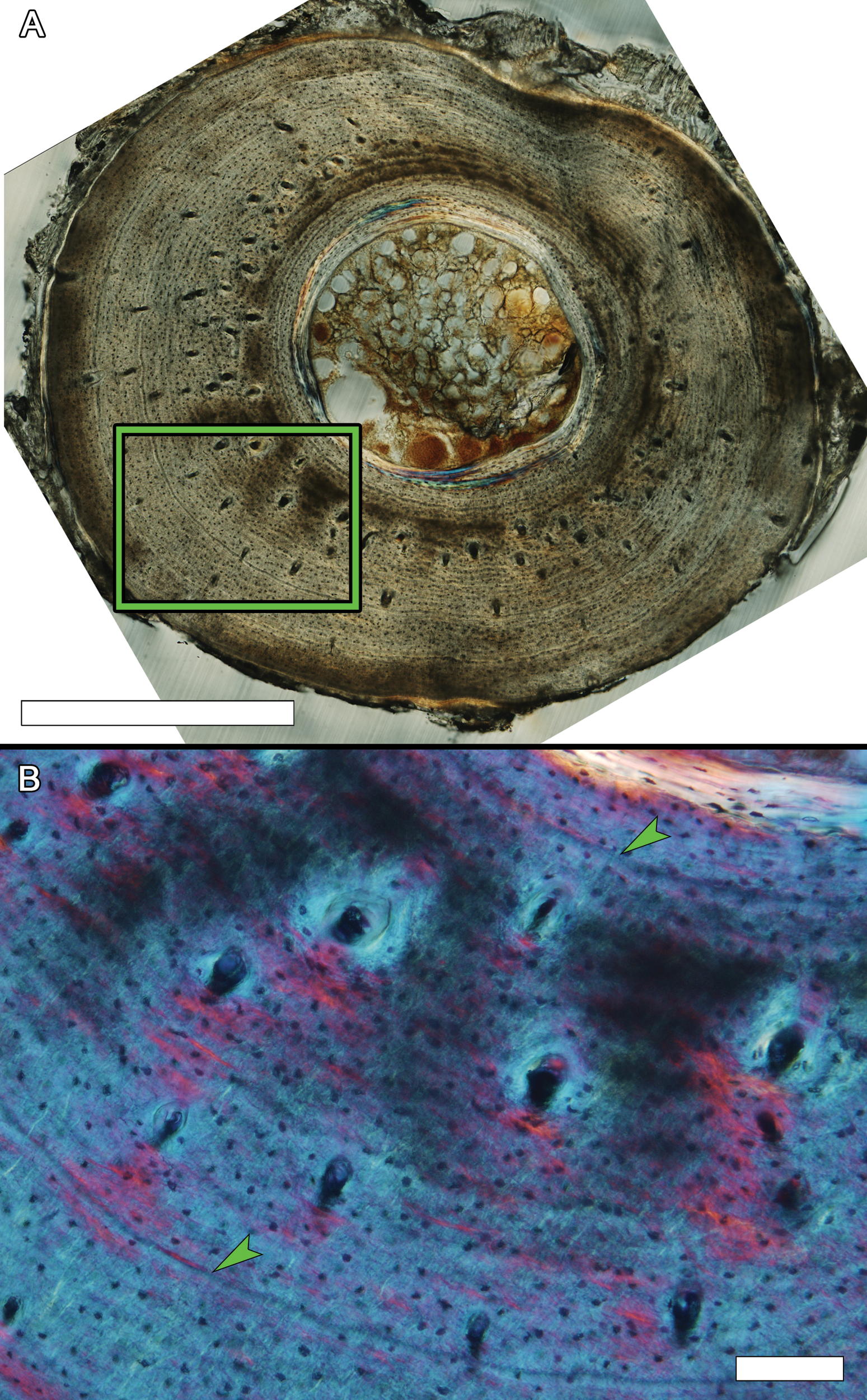

Supplement: Figure S4 — (A) Transverse section. Scale bar, 1 mm. (B) Enlargement of the area from (A) within the green box, photographed using a full lambda (530 nm) plate to reveal fiber orientation. Two growth marks are visible in this enlargement (arrows). The cortex is of highly organized lamellar tissue with a sparse scattering of longitudinal vascular canals. Scale bar, 100 µm. [file peerj-02-422-s006.png]

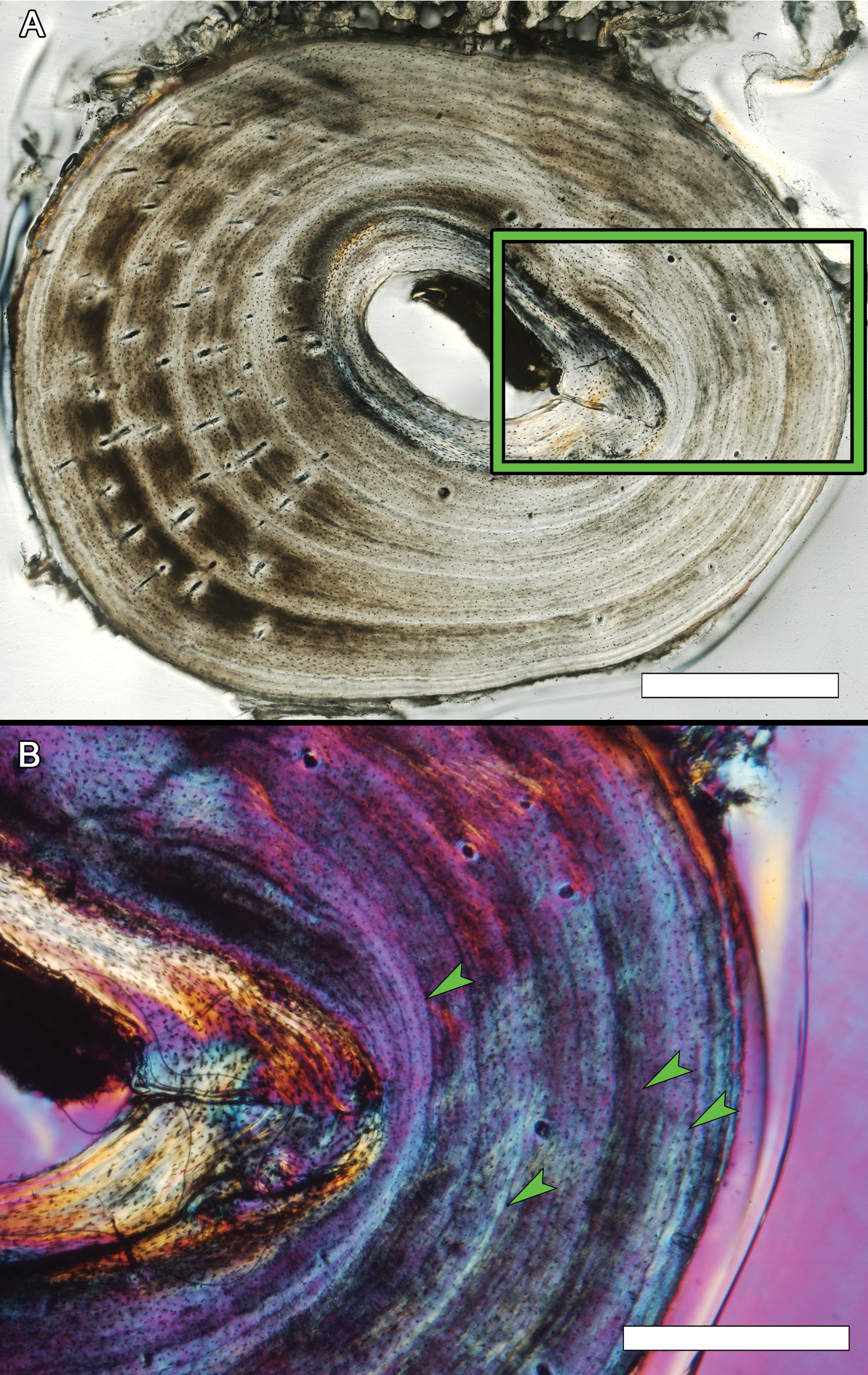

Supplement: Figure S5 — (A) Transverse section. Scale bar, 1 mm. (B) Enlargement of the area from (A) within the green box, photographed using a full lambda (530 nm) plate to reveal fiber orientation. The cortex is of highly organized lamellar tissue and is nearly avascular. Green arrows point to four LAGs in this region. A well-developed endosteal layer (left) cuts across primary tissue and in places consists of secondary osteons, indicating medullary drift. Scale bar, 500 µm. [file peerj-02-422-s007.png]

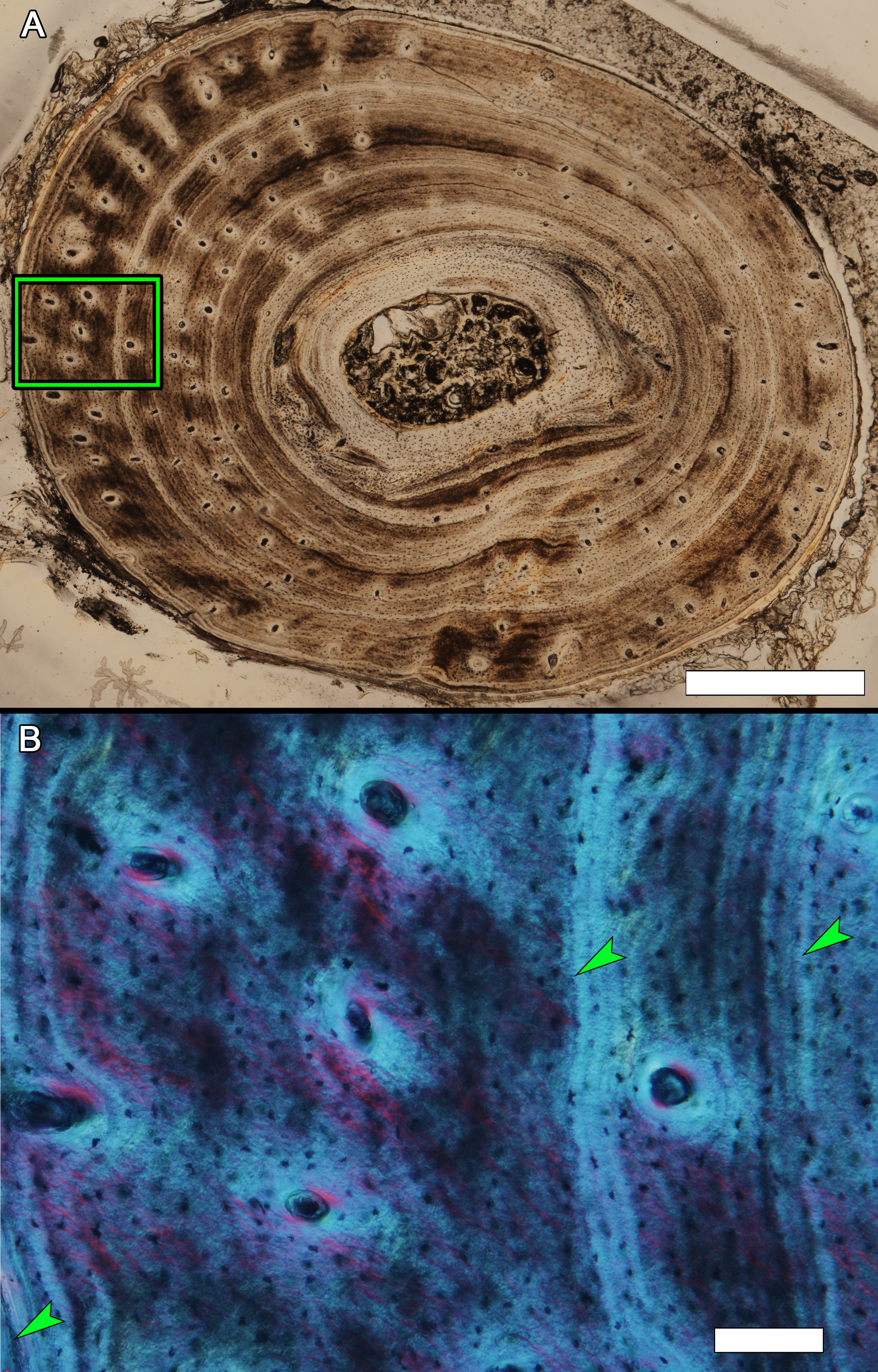

Supplement: Figure S6 — (A) Transverse section. The well-developed endosteal layer cuts across primary tissue and in some areas is replaced by secondary osteons, indicating medullary drift. Scale bar, 1 mm. (B) Enlargement of the area from (A) within the green box, photographed using a full lambda (530 nm) plate to reveal fiber orientation. The cortex is made of highly organized lamellar tissue containing scattered longitudinal simple primary canals and primary osteons. Three CGMs (arrows) are evident in the enlargement. Scale bar, 100 µm. [file peerj-02-422-s008.png]

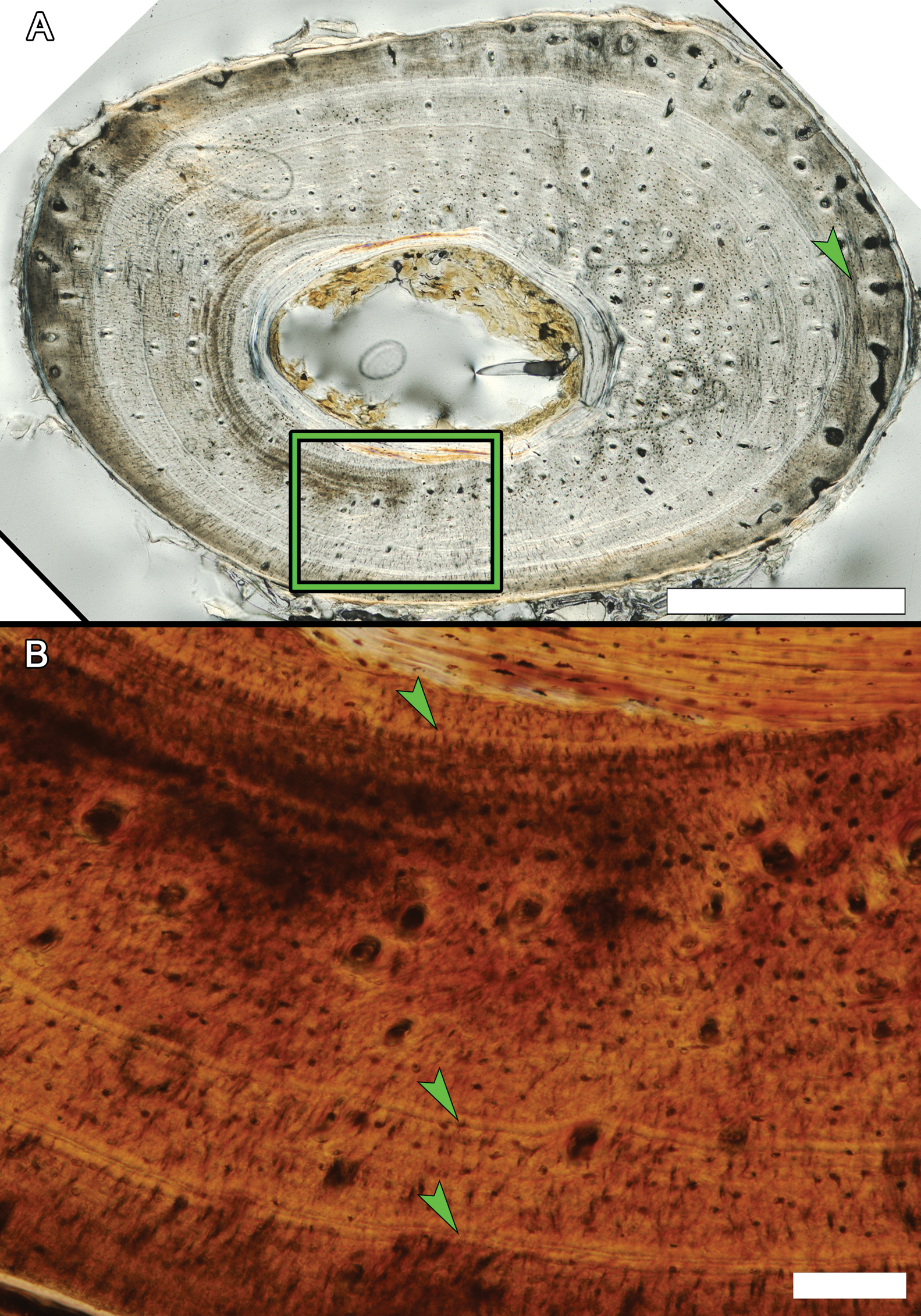

Supplement: Figure S7 — (A) Transverse section. Periosteal attachment fibers (arrow) are evident even at low magnification. Scale bar, 1 mm. (B) Enlargement of the area from (A) within the green box, photographed using a full lambda (530 nm) plate to reveal fiber orientation. Here the cortex is lamellar, with sparsely scattered longitudinal vascular canals. The innermost LAG is partially destroyed by medullary expansion and two more are visible within the cortex (green arrows). Scale bar, 100 µm. [file peerj-02-422-s009.png]

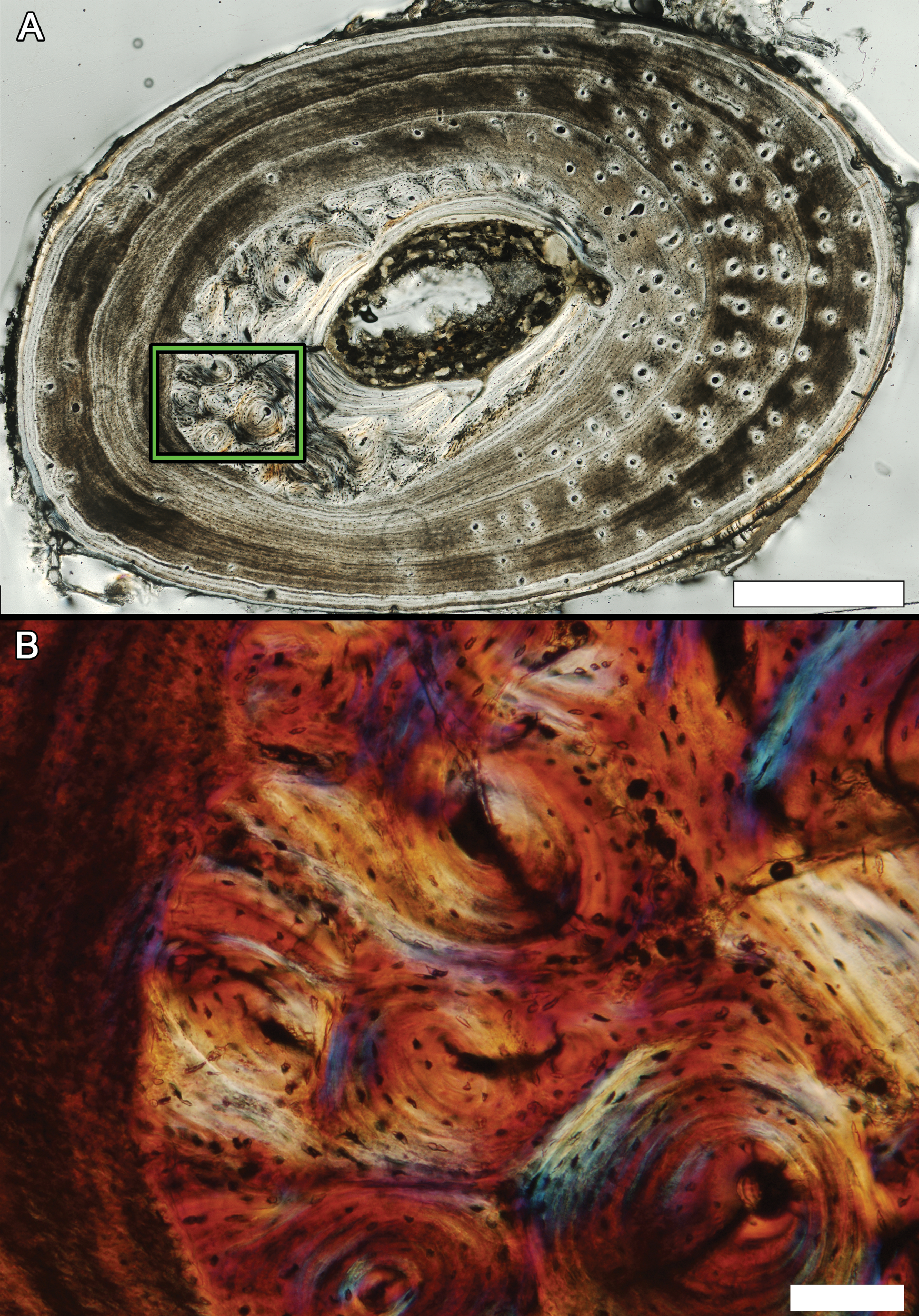

Supplement: Figure S8 — (A) Transverse section. The high degree of endosteal remodeling is apparent even at low magnification, especially in the region highlighted within the green box. Scale bar, 1 mm. (B) Enlargement of the area from (A) within the green box, photographed using a full lambda (530 nm) plate to reveal fiber orientation. Secondary reconstruction occurred within the inner cortex due to medullary drift, resulting in large, overlapping secondary osteons. Scale bar, 100 µm. [file peerj-02-422-s010.png]

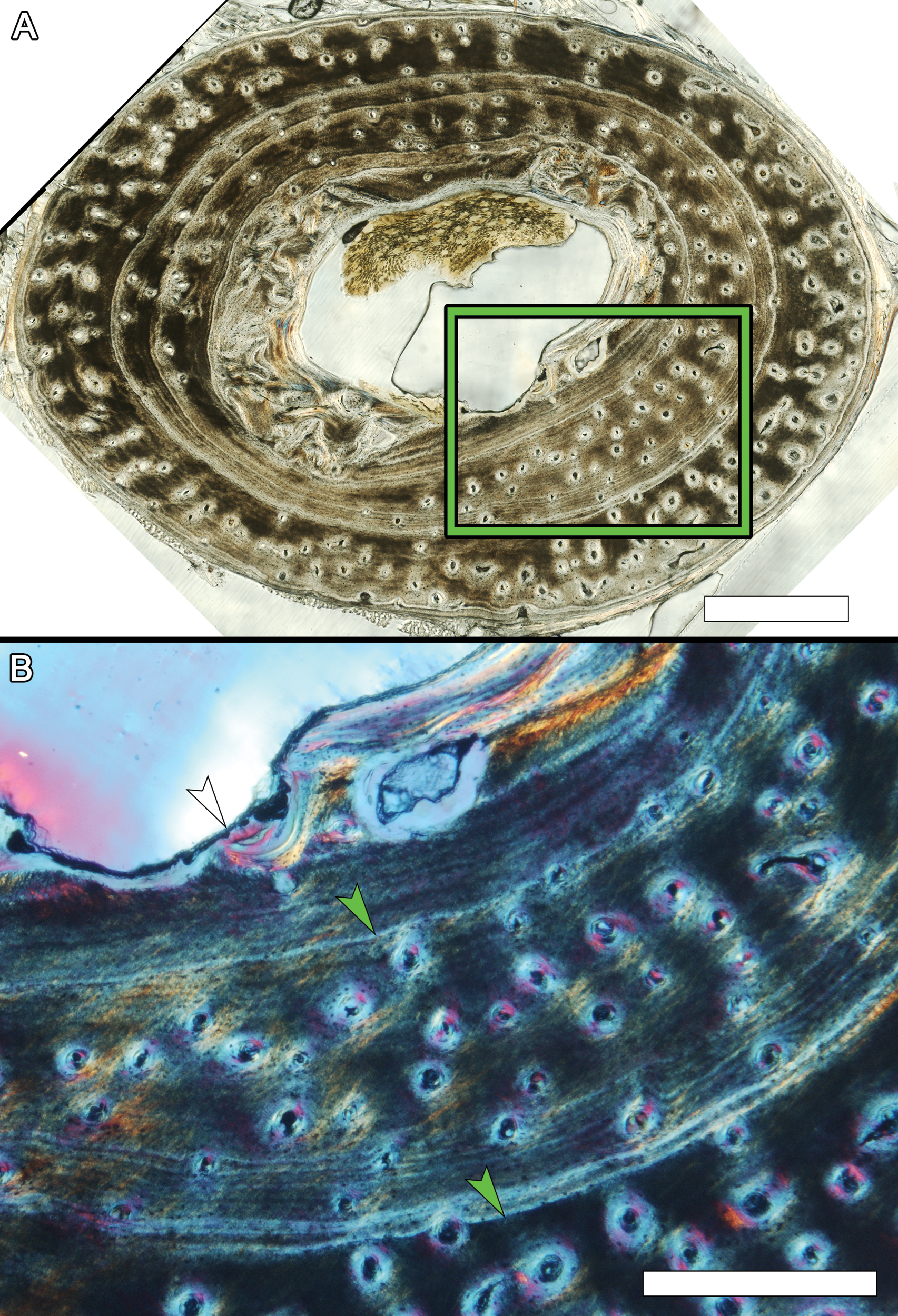

Supplement: Figure S9 — (A) Transverse section. Scale bar, 1 mm. (B) Enlargement of the area from (A) within the green box, photographed using a full lambda (530 nm) plate to reveal fiber orientation. Two LAGs are marked by green arrows. Medullary expansion was active in this region as indicated by the partially resorbed endosteal layer (white arrow) and resorption cavity within the inner cortex. Tissue organization within the cortex is lamellar, and vascularity is longitudinal. Scale bar, 500 µm. [file peerj-02-422-s011.png]

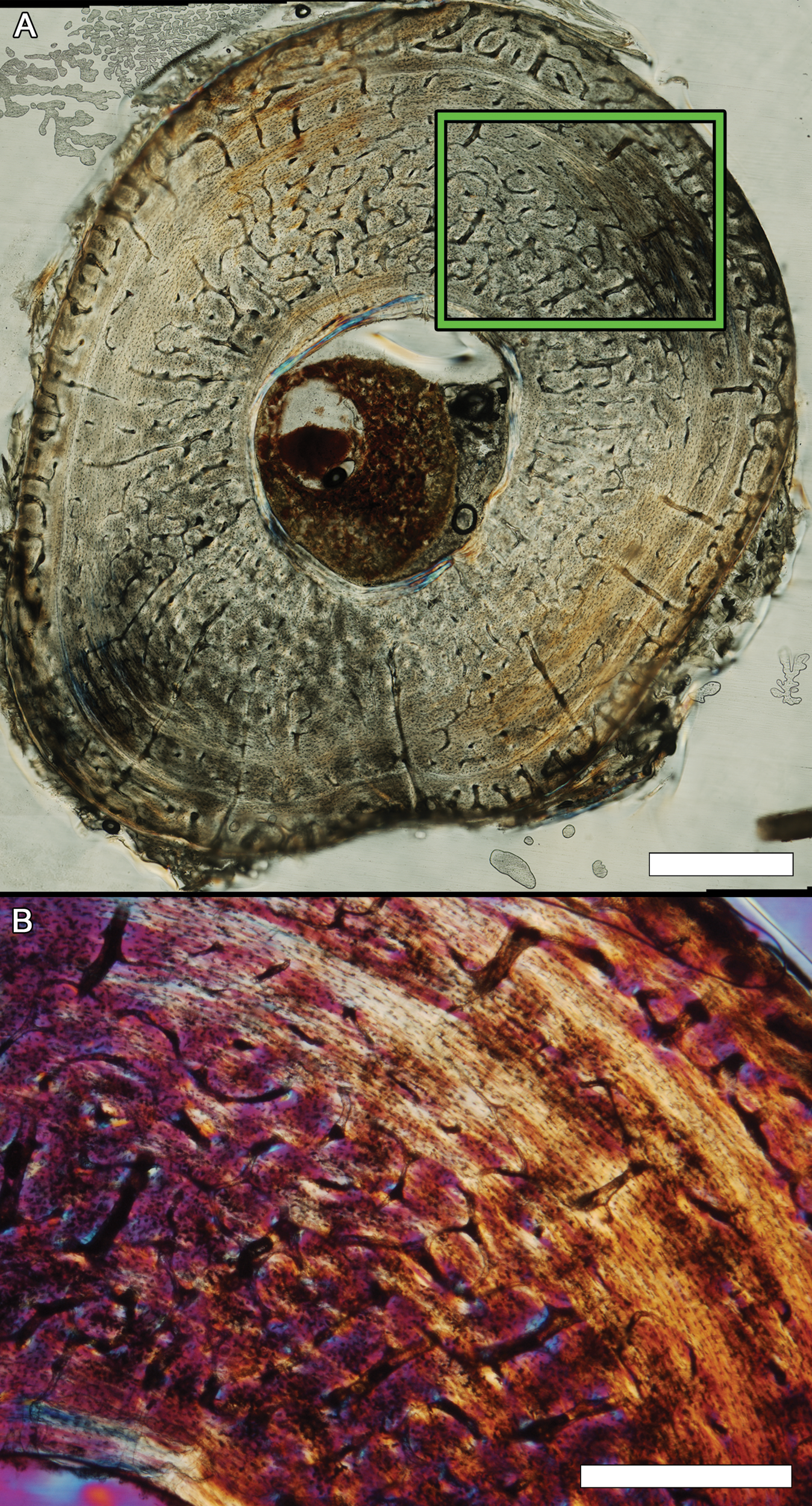

Supplement: Figure S10 — (A) Transverse section shows a combination of anastomosing and radial vascular canals. Scale bar, 1 mm. (B) Enlargement of the area from (A) within the green box, photographed using a full lambda (530 nm) plate to reveal fiber orientation. Tissue within the innermost cortex is woven but is parallel-fibered by mid cortex. Scale bar, 500 µm. [file peerj-02-422-s012.png]

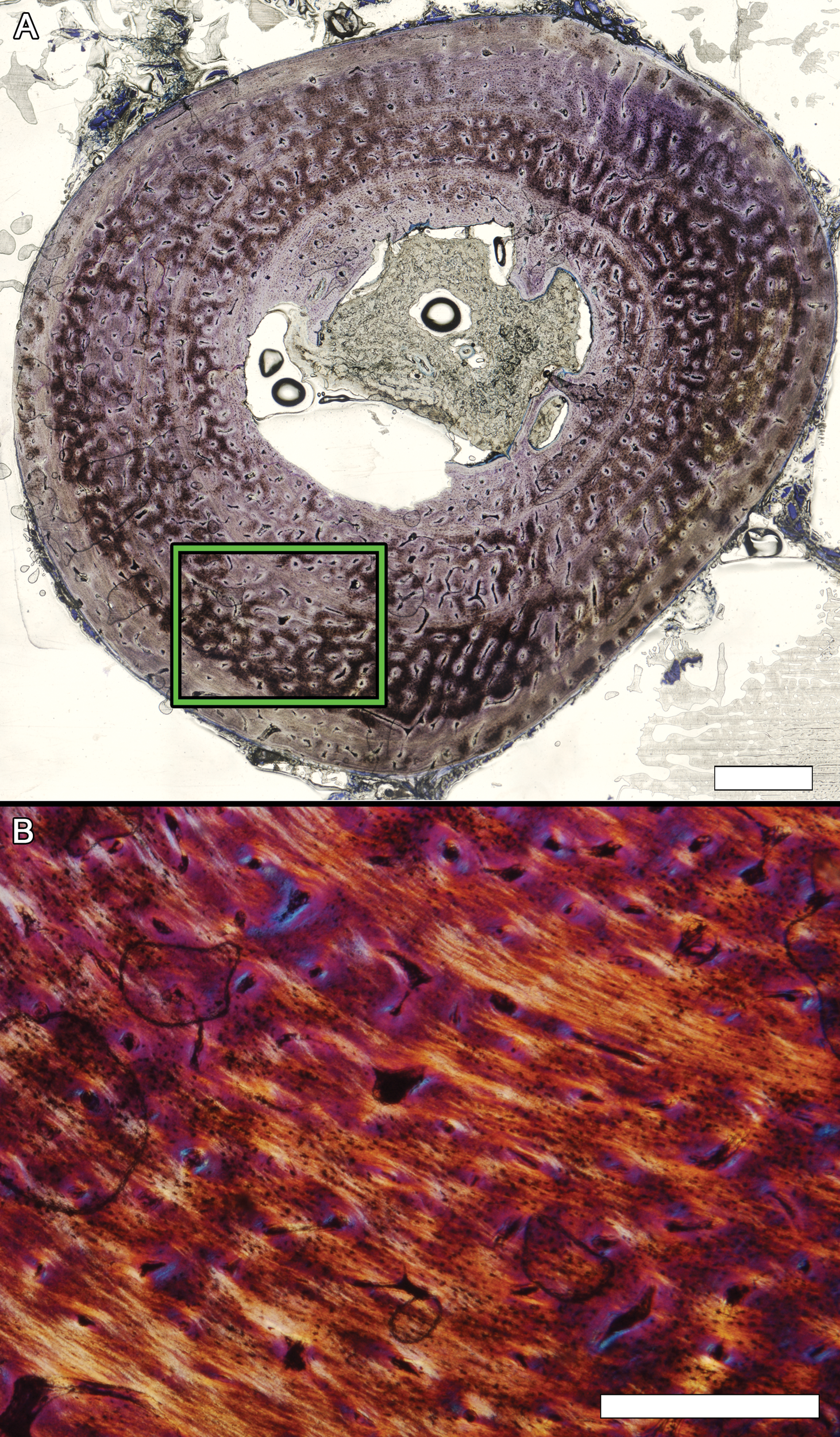

Supplement: Figure S11 — (A) Transverse section, stained with Toluidine chloride. Scale bar, 1 mm. (B) Enlargement of the area from (A) within the green box, photographed using a full lambda (530 nm) plate to reveal fiber orientation. The cortical tissue is loosely parallel-fibered. Scale bar, 500 µm. [file peerj-02-422-s013.png]

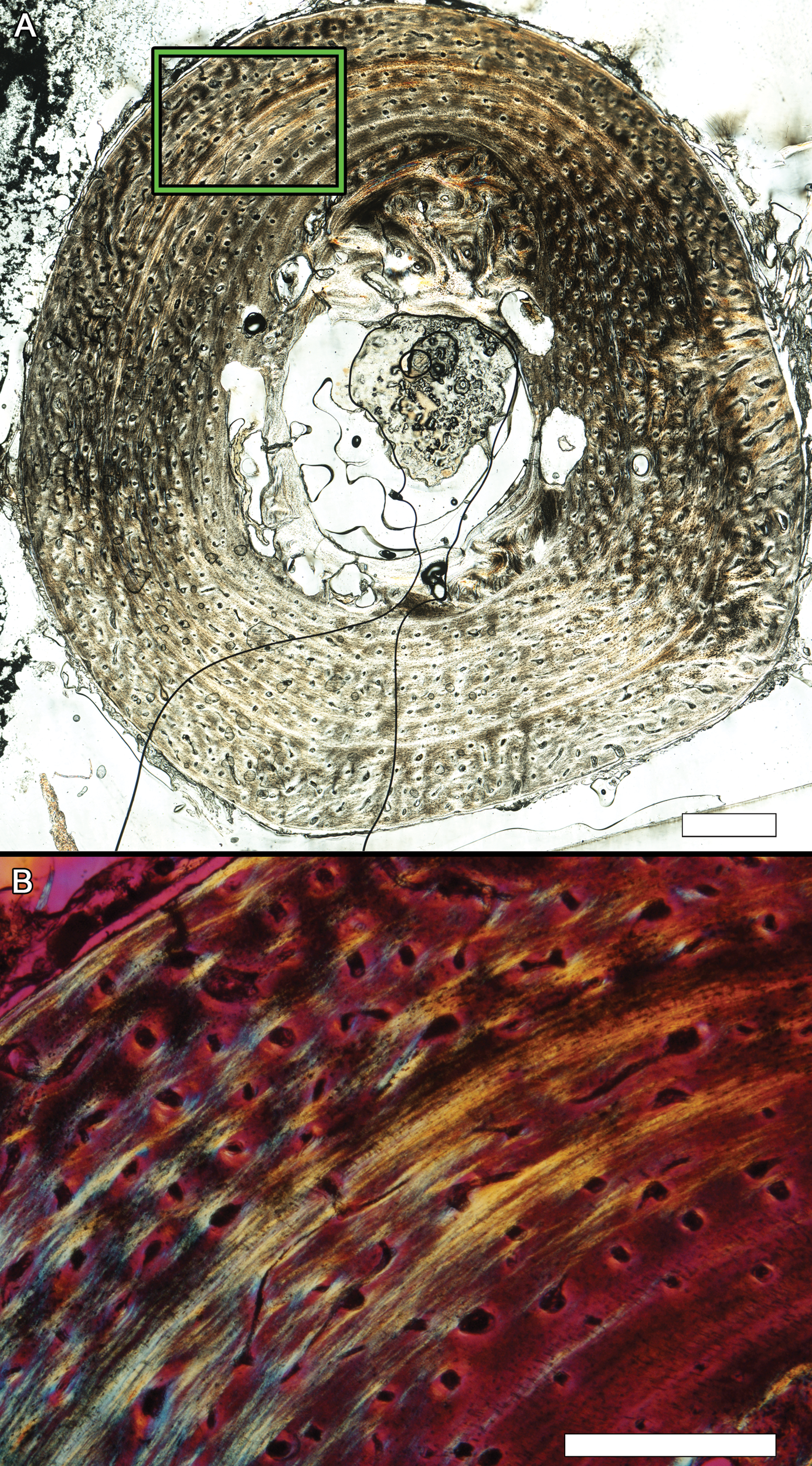

Supplement: Figure S12 — (A) Transverse section photographed using circularly polarized light. Medullary drift is evident by the concentration of secondary tissue endosteally, and active medullary expansion is revealed by large resorption cavities within the inner cortex. Scale bar, 1 mm. (B) Enlargement of the area from (A) within the green box, photographed using a full lambda (530 nm) plate to reveal fiber orientation. The cortical tissue is loosely parallel-fibered. Scale bar, 500 µm. [file peerj-02-422-s014.png]

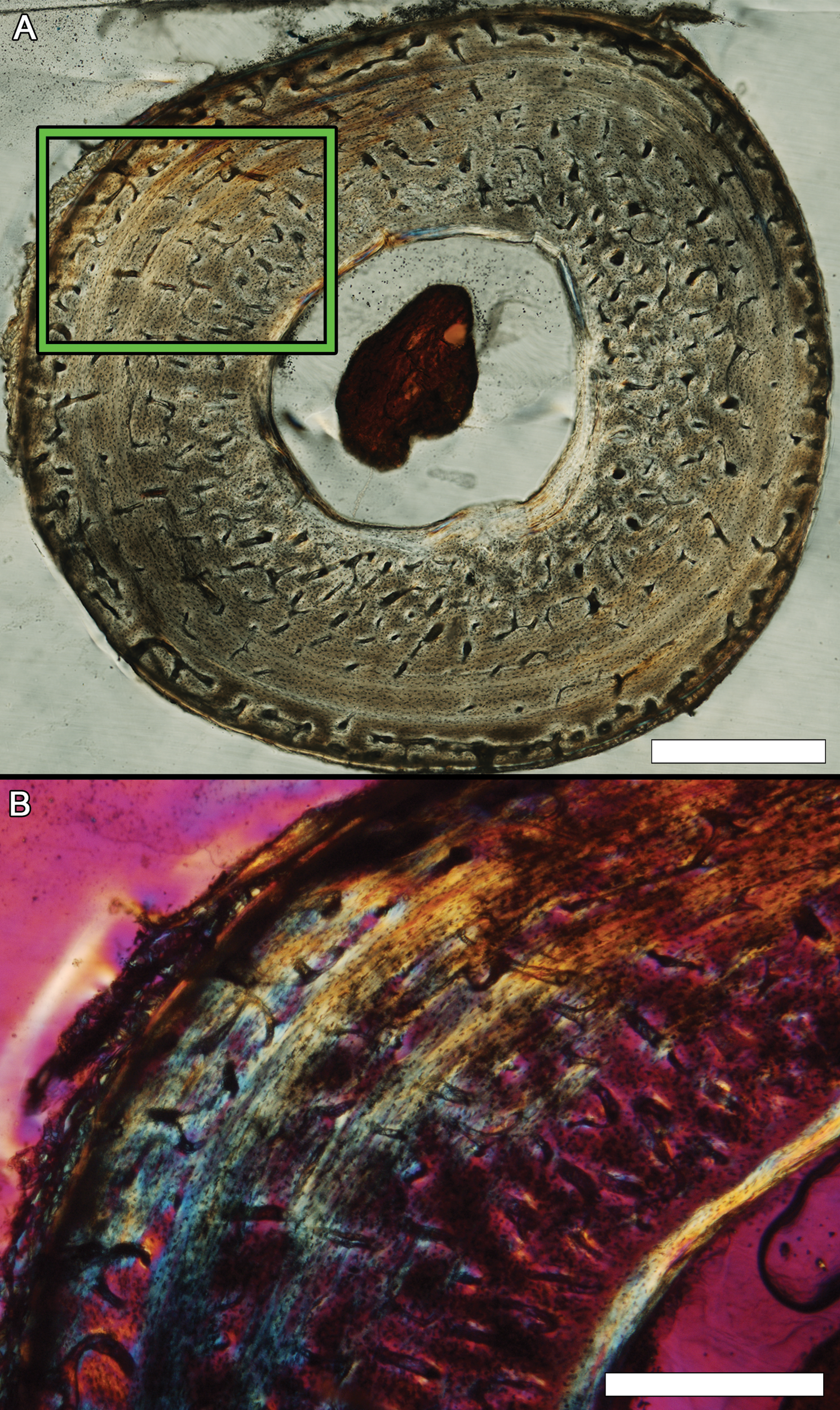

Supplement: Figure S13 — (A) Transverse section. Scale bar, 1 mm. (B) Enlargement of the area from (A) within the green box, photographed using a full lambda (530 nm) plate to reveal fiber orientation. Fibers are woven within the inner cortex but are parallel-fibered by mid cortex. Vascular canals are obliquely anastomosing. Scale bar, 500 µm. [file peerj-02-422-s015.png]

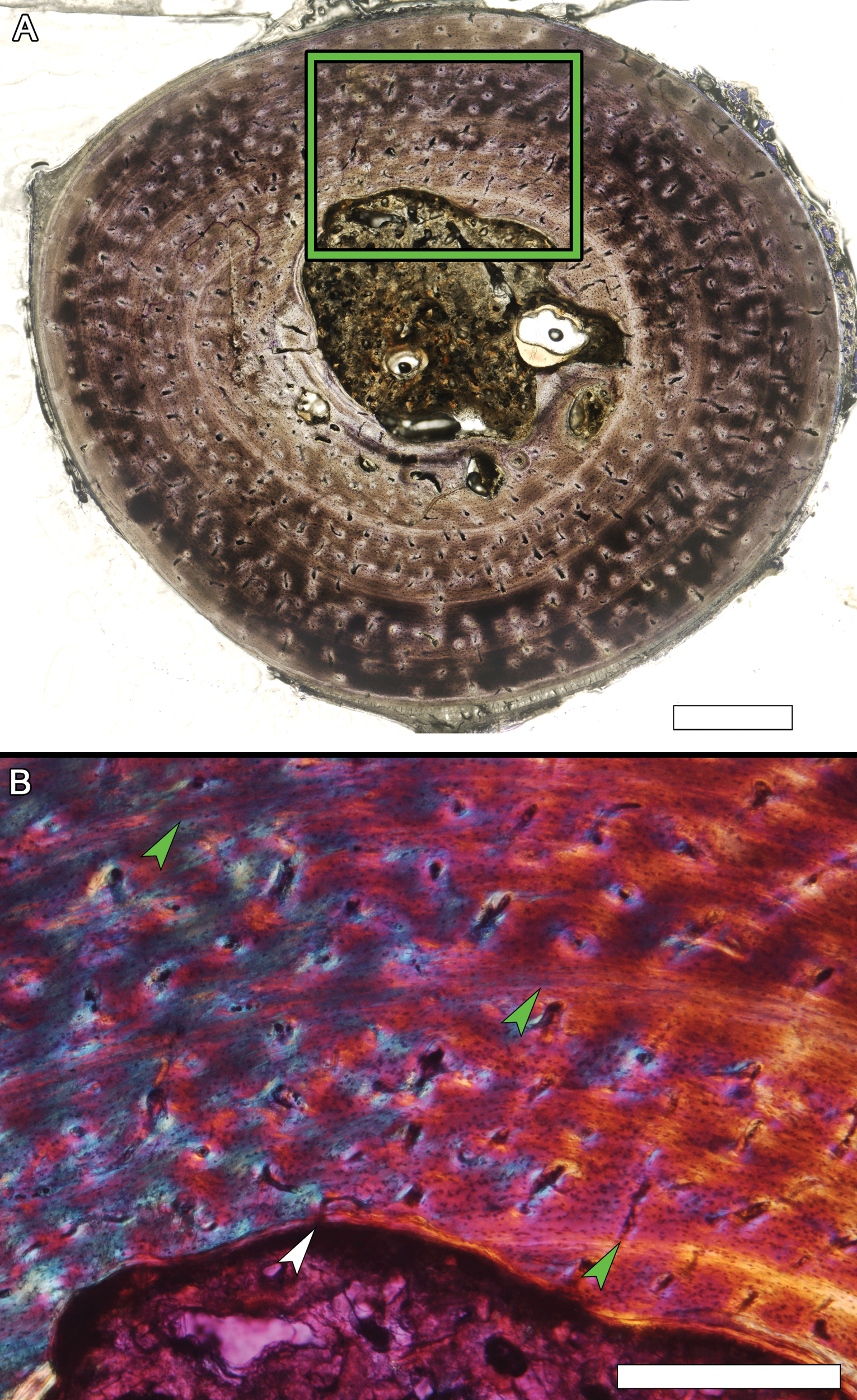

Supplement: Figure S14 — (A) Transverse section, stained with Toluidine chloride. Scale bar, 1 mm. (B) Enlargement of the area from (A) within the green box, photographed using a full lambda (530 nm) plate to reveal fiber orientation. Medullary expansion is evident by the resorbed region of inner cortex (white arrow) and the partial destruction of the innermost annulus (green arrow). Two more annuli (green arrows) are present within the cortex. Tissue is parallel-fibered and most vascular canals are either longitudinal or somewhat anastomosing. Scale bar, 500 µm. [file peerj-02-422-s016.png]

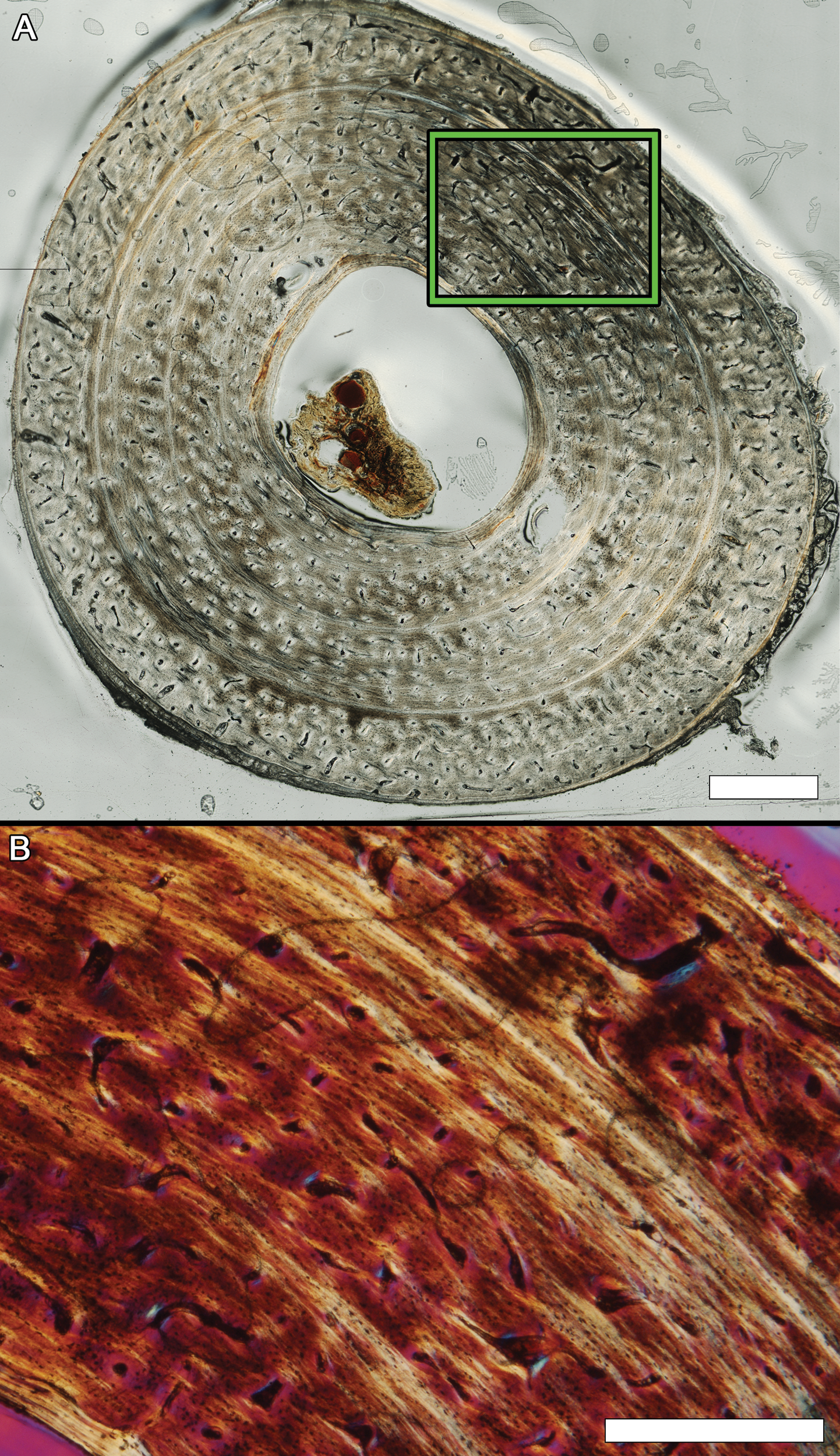

Supplement: Figure S15 — (A) Transverse section. Scale bar, 1 mm. (B) Enlargement of the area from (A) within the green box, photographed using a full lambda (530 nm) plate to reveal fiber orientation. Here, tissue fibers are arranged loosely in parallel. Vascular canals are longitudinal and anastomosing. Scale bar, 500 µm. [file peerj-02-422-s017.png]

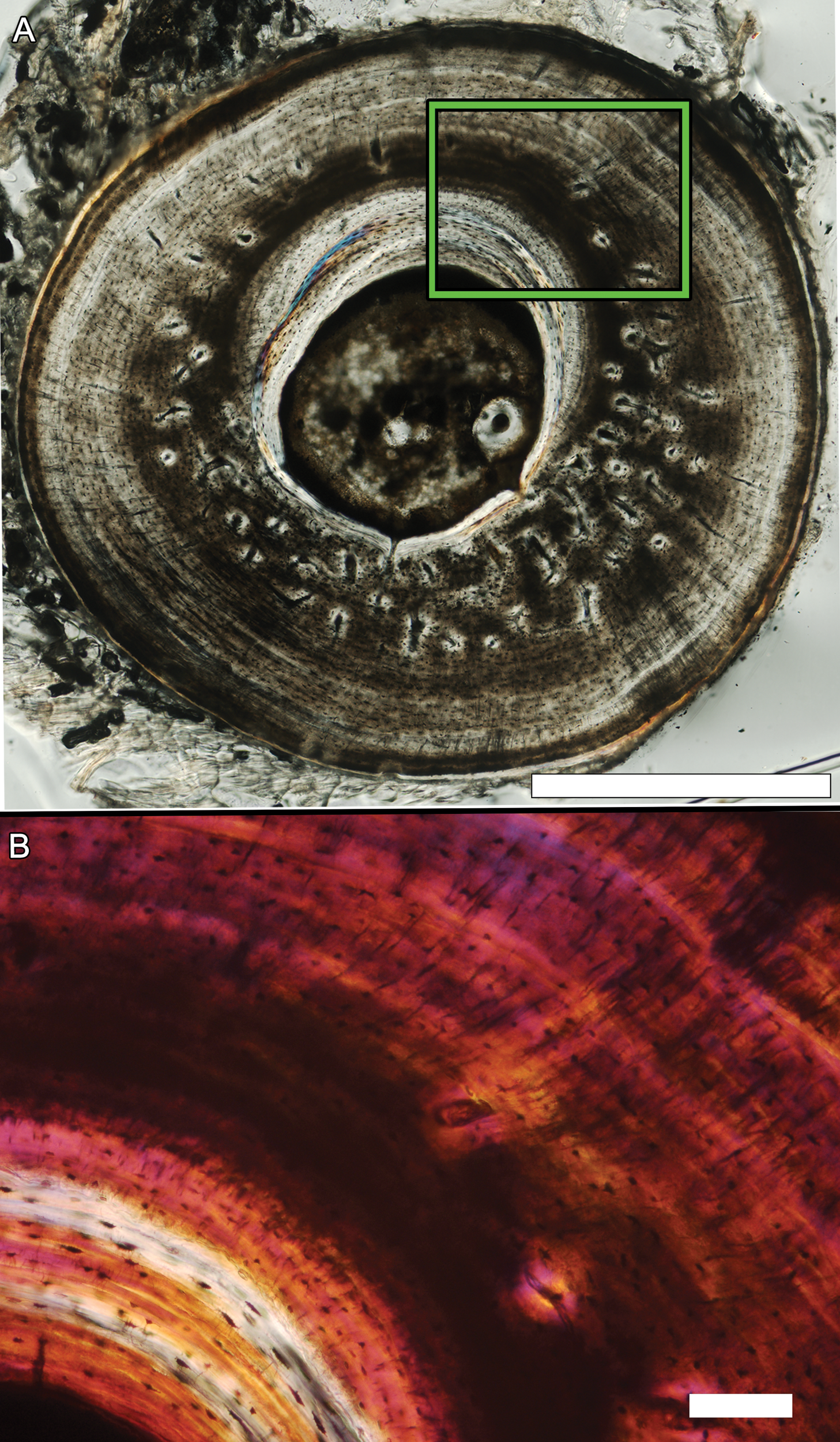

Supplement: Figure S16 — (A) Transverse section. The innermost cortex is partially destroyed by medullary drift. Scale bar, 1 mm. (B) Enlargement of the area from (A) within the green box, photographed using a full lambda (530 nm) plate to reveal fiber orientation. This region is largely avascular and the lamellar tissue contains numerous periosteal attachment fibers. Scale bar, 100 µm. [file peerj-02-422-s018.png]

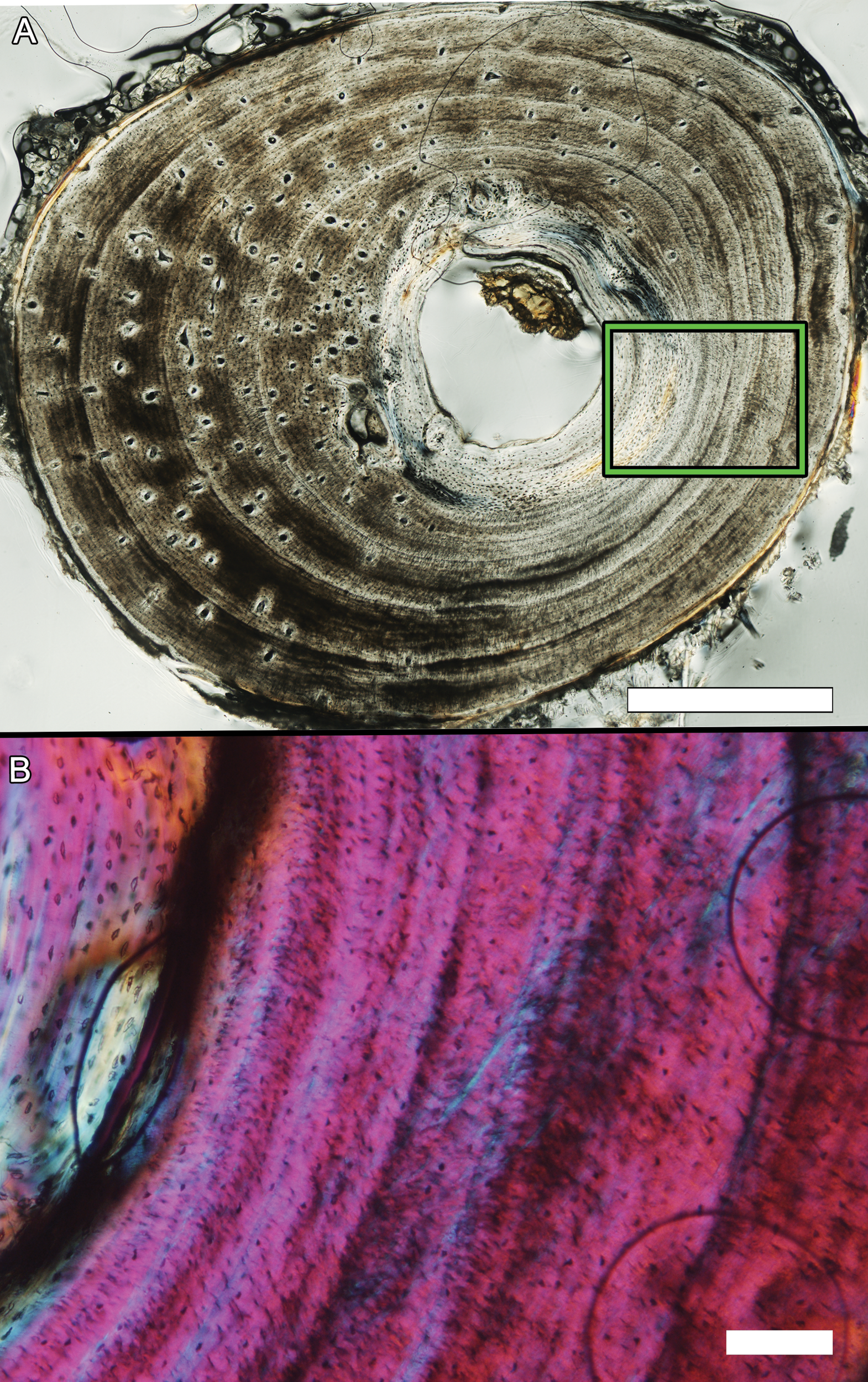

Supplement: Figure S17 — (A) Transverse section. Scale bar, 1 mm. (B) Enlargement of the area from (A) within the green box, photographed using a full lambda (530 nm) plate to reveal fiber orientation. Highly organized lamellar fibers contain scattered osteocytes. Periosteal attachment fibers are abundant. Circles on the right of the image are preparation artifacts. Scale bar, 100 µm. [file peerj-02-422-s019.png]

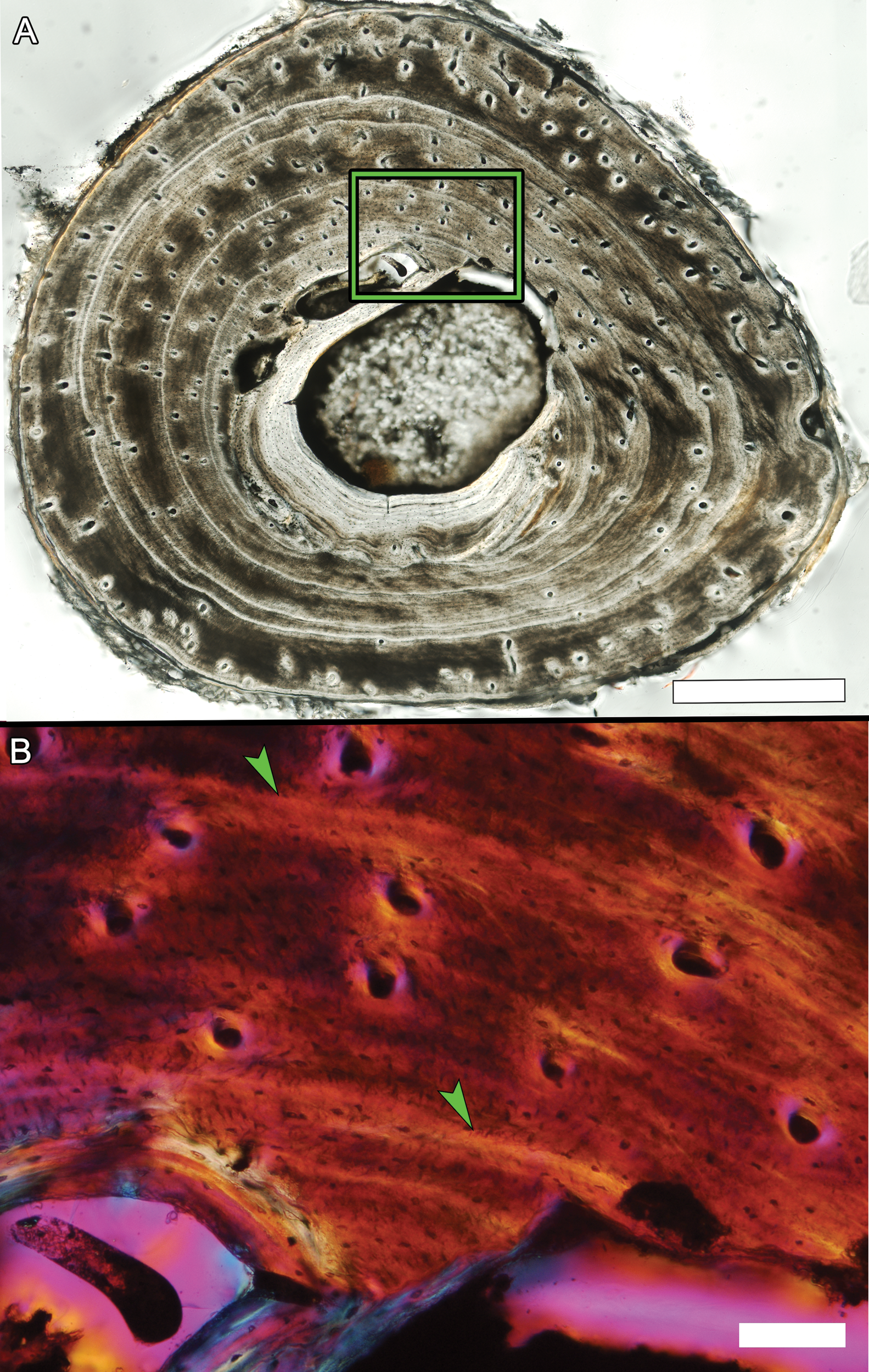

Supplement: Figure S18 — (A) Transverse section. Medullary expansion and resorption cavities removed the innermost primary cortex. Scale bar, 1 mm. (B) Enlargement of the area from (A) within the green box, photographed using a full lambda (530 nm) plate to reveal fiber orientation. Two LAGs are visible in this region (arrows), partially destroyed by medullary expansion. Tissue within the cortex is lamellar to somewhat parallel-fibered and vascular canals are longitudinal. Scale bar, 100 µm. [file peerj-02-422-s020.png]

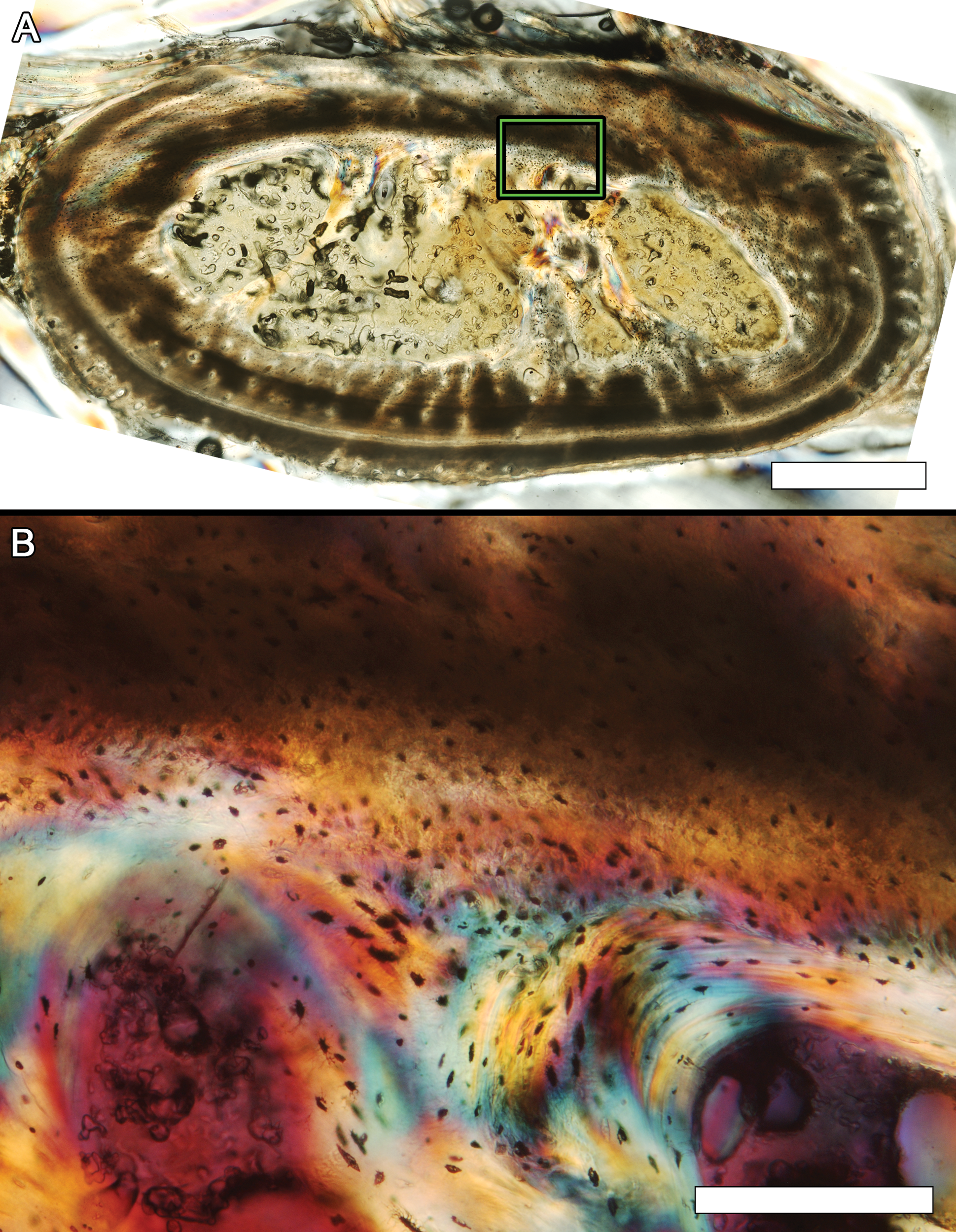

Supplement: Figure S19 — (A) Transverse section. Due to the size of the medullary cavity, it is likely some CGMs were lost to resorption. Scale bar, 1 mm. (B) Enlargement of the area from (A) within the green box, photographed using a full lambda (530 nm) plate to reveal fiber orientation. The lower half of the image contains a strut of remodeled bone within the medullary cavity, and the upper half shows the largely avascular inner cortex. Scale bar, 200 µm. [file peerj-02-422-s021.png]

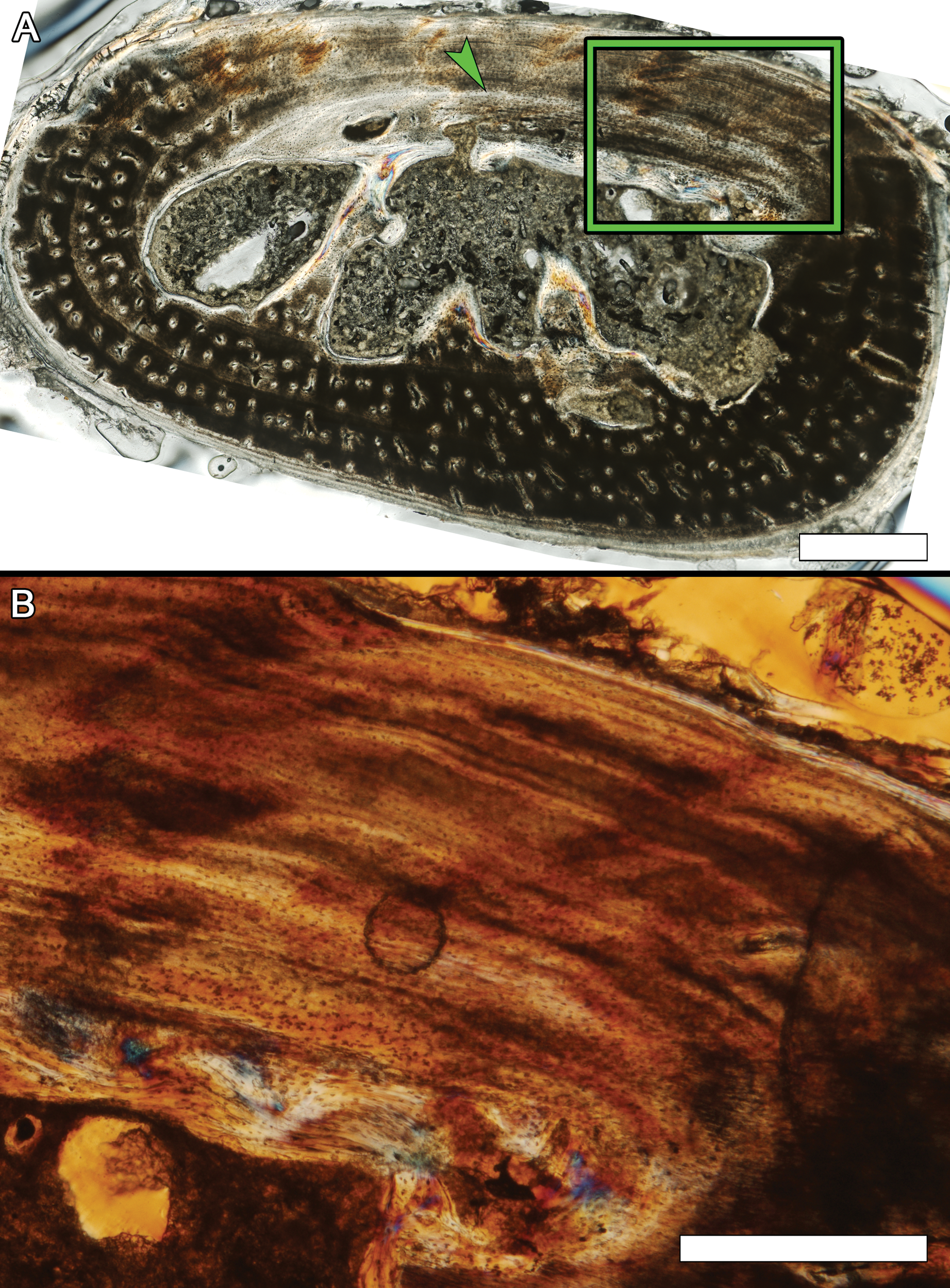

Supplement: Figure S20 — (A) Transverse section. The innermost annulus (arrow) is partially destroyed by expansion of the large medullary cavity. Scale bar, 1 mm. (B) Enlargement of the area from (A) within the green box, photographed using a full lambda (530 nm) plate to reveal fiber orientation. Tissue in the region is avascular and lamellar. Periosteal attachment fibers radiate outward within the cortex. Scale bar, 500 µm. [file peerj-02-422-s022.png]

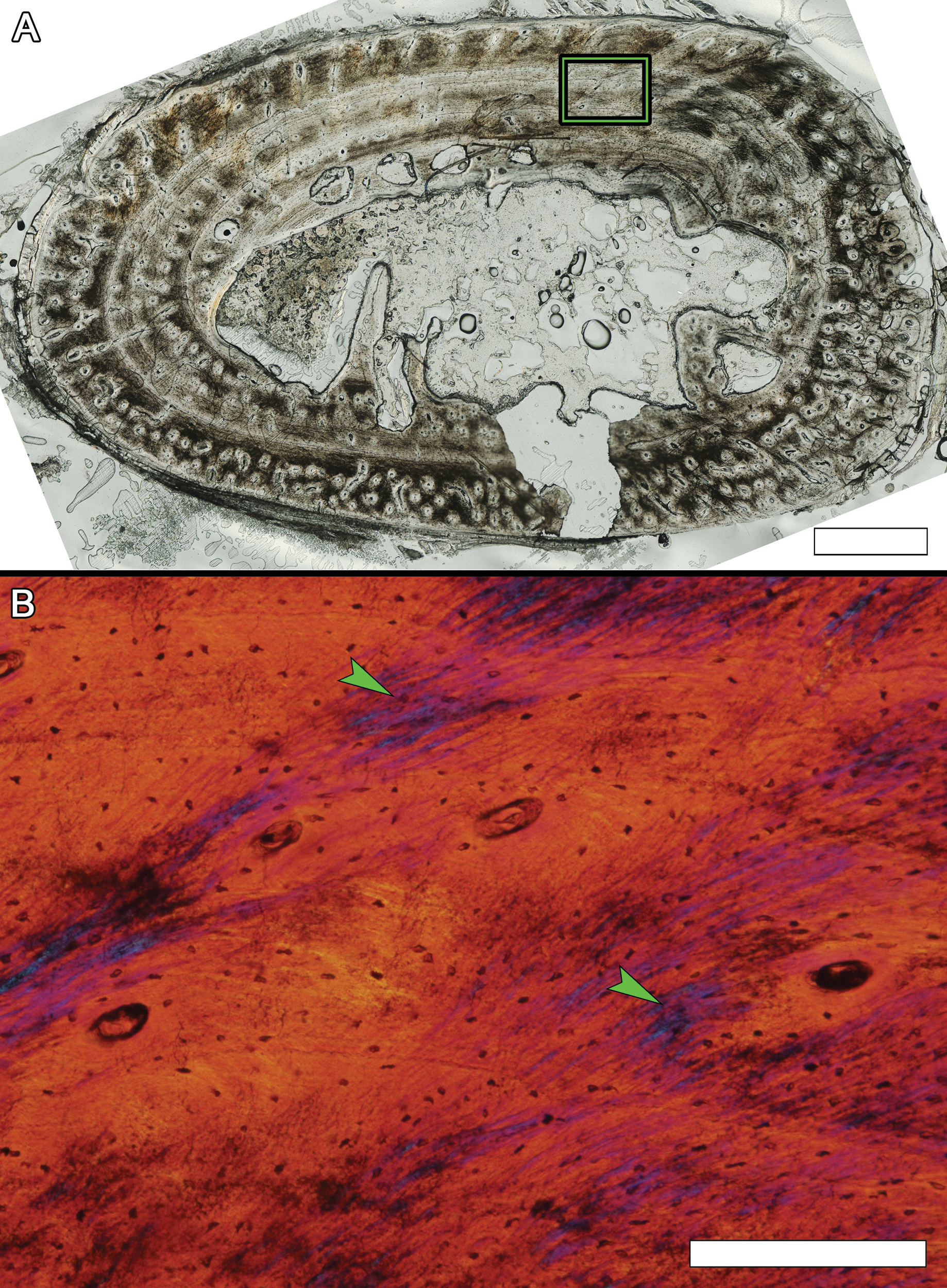

Supplement: Figure S21 — (A) Transverse section. The innermost CGM is nearly destroyed by the large marrow cavity and surrounding resorption cavities. Missing cortex in the bottom of the image is an artifact of preparation. Scale bar, 1 mm. (B) Enlargement of the area from (A) within the green box, photographed using a full lambda (530 nm) plate to reveal fiber orientation. Tissue in this area is lamellar to parallel-fibered. Periosteal attachment fibers (arrows) radiate frequently within the cortex. Scale bar, 200 µm. [file peerj-02-422-s023.png]

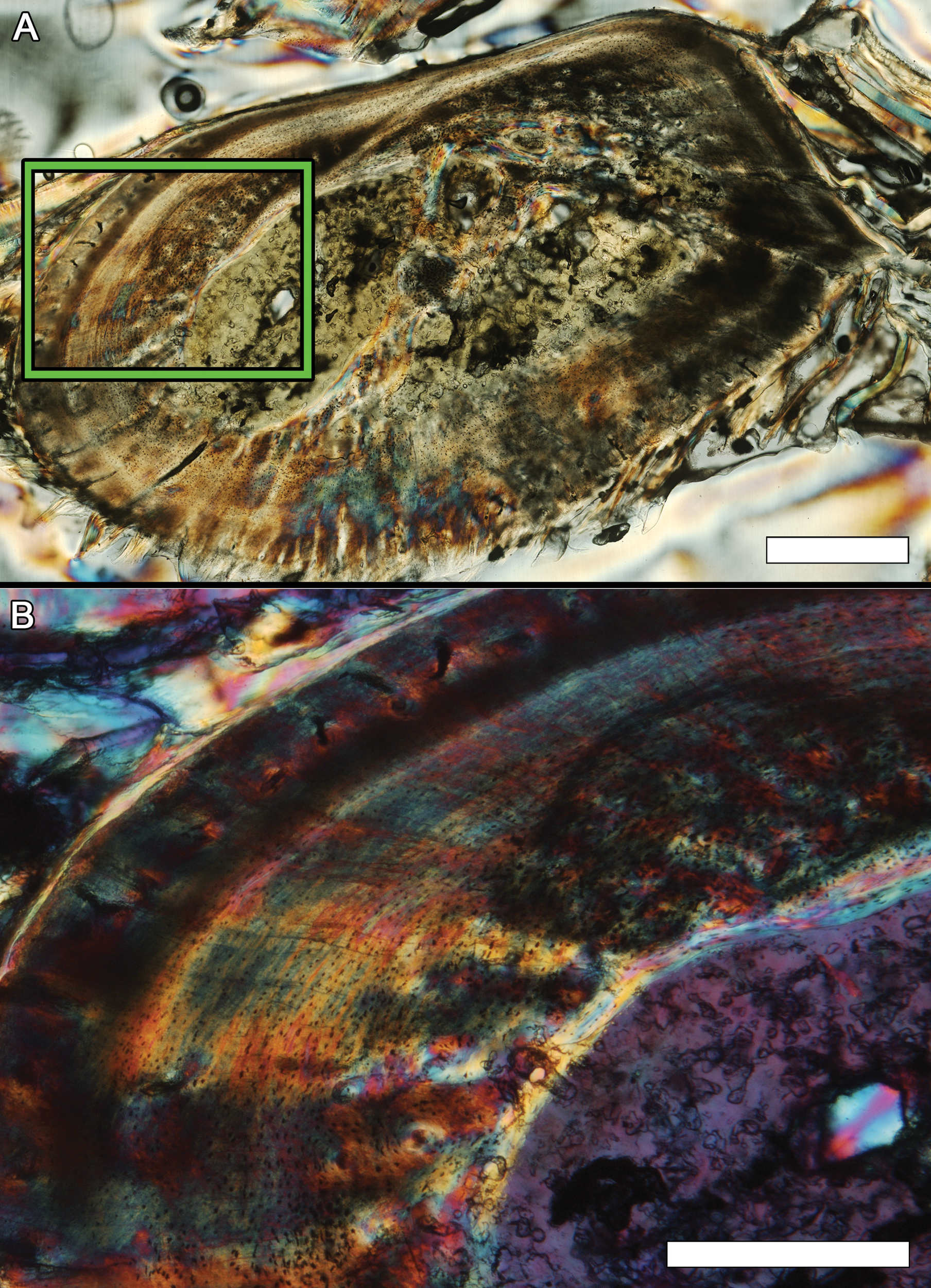

Supplement: Figure S22 — (A) Transverse section. Scale bar, 1 mm. (B) Enlargement of the area from (A) within the green box, photographed using a full lambda (530 nm) plate to reveal fiber orientation. The lamellar cortex contains scattered vascular canals, but the mid cortex is largely avascular. Scale bar, 500 µm. [file peerj-02-422-s024.png]

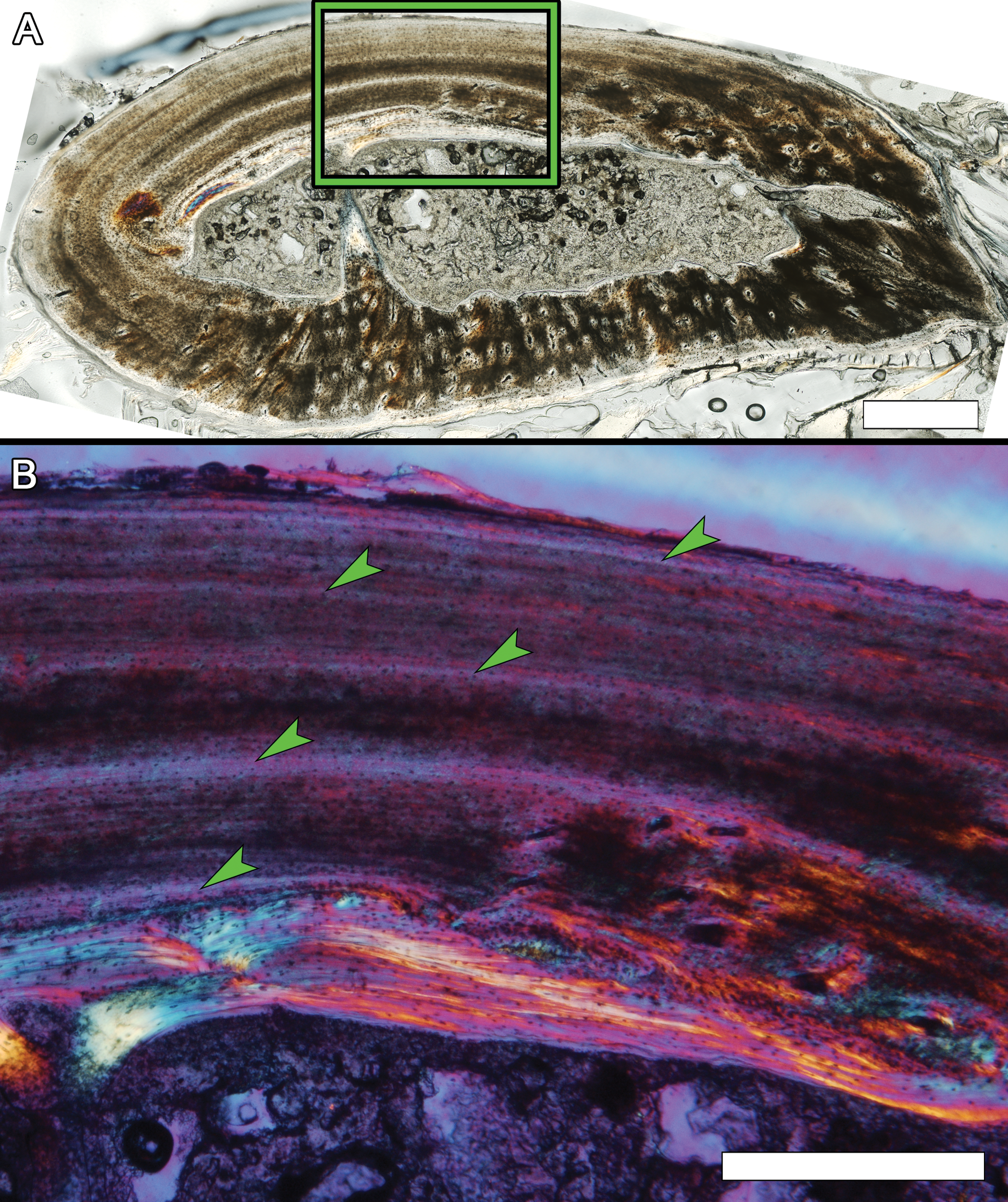

Supplement: Figure S23 — (A) Transverse section. Medullary expansion has partially destroyed several CGMs within the cortex. Scale bar, 1 mm. (B) Enlargement of the area from (A) within the green box, photographed using a full lambda (530 nm) plate to reveal fiber orientation. This region is predominately avascular and lamellar with scattered osteocytes. Five annuli followed by LAGs (arrows) appear as lighter bands within the cortex. Scale bar, 500 µm. [file peerj-02-422-s025.png]

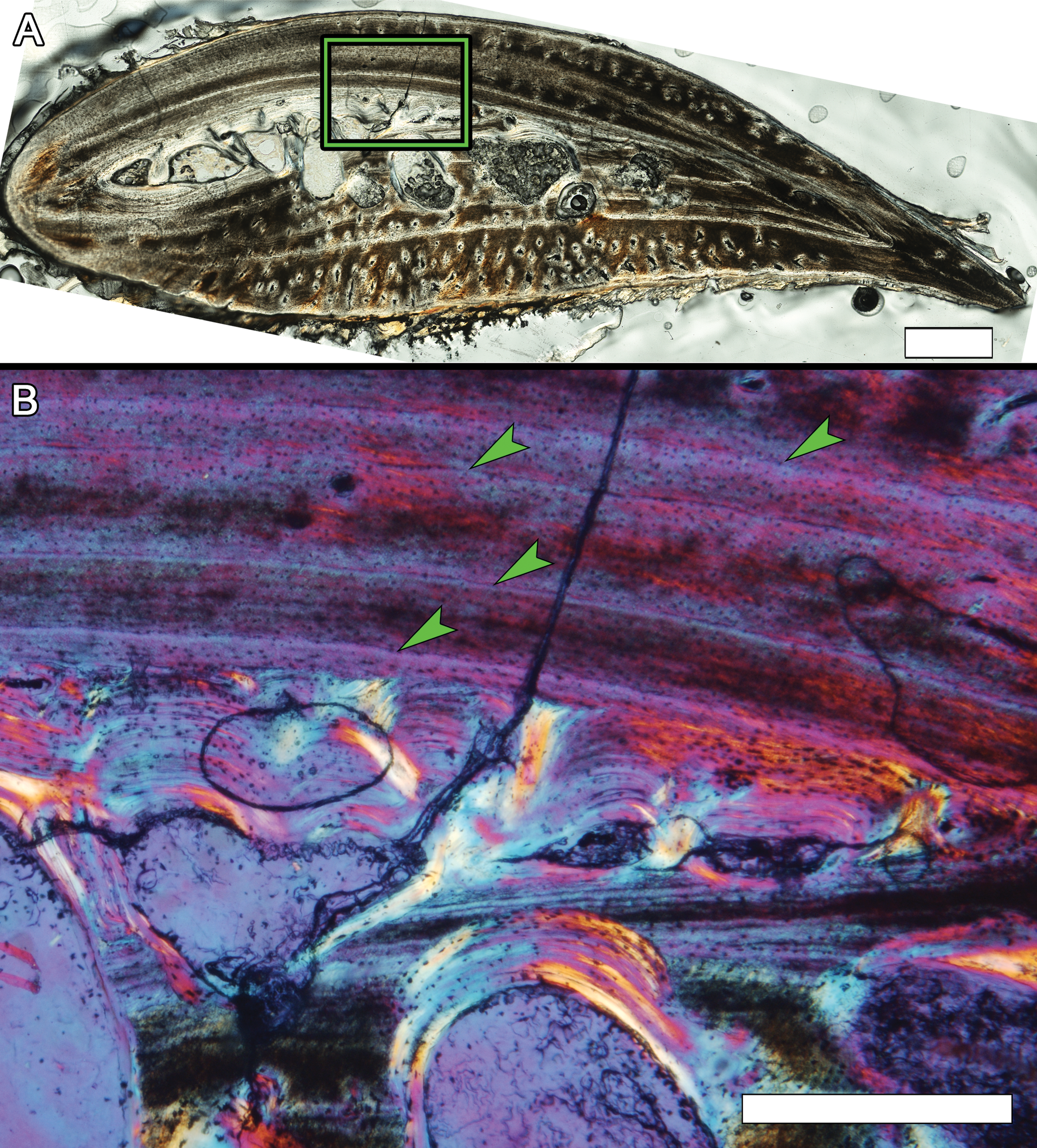

Supplement: Figure 24 — (A) Transverse section. In several areas CGMs are partially destroyed by large resorption cavities. Scale bar, 1 mm. (B) Enlargement of the area from (A) within the green box, photographed using a full lambda (530 nm) plate to reveal fiber orientation. Near the center of the image, remodeled secondary tissue borders the medullary cavity, and resorption cavities are present in the lower half. Primary tissue in this area is fairly avascular and lamellar. Four annuli followed by LAGs (arrows) appear as light bands within the cortex. Scale bar, 500 µm. [file peerj-02-422-s026.png]

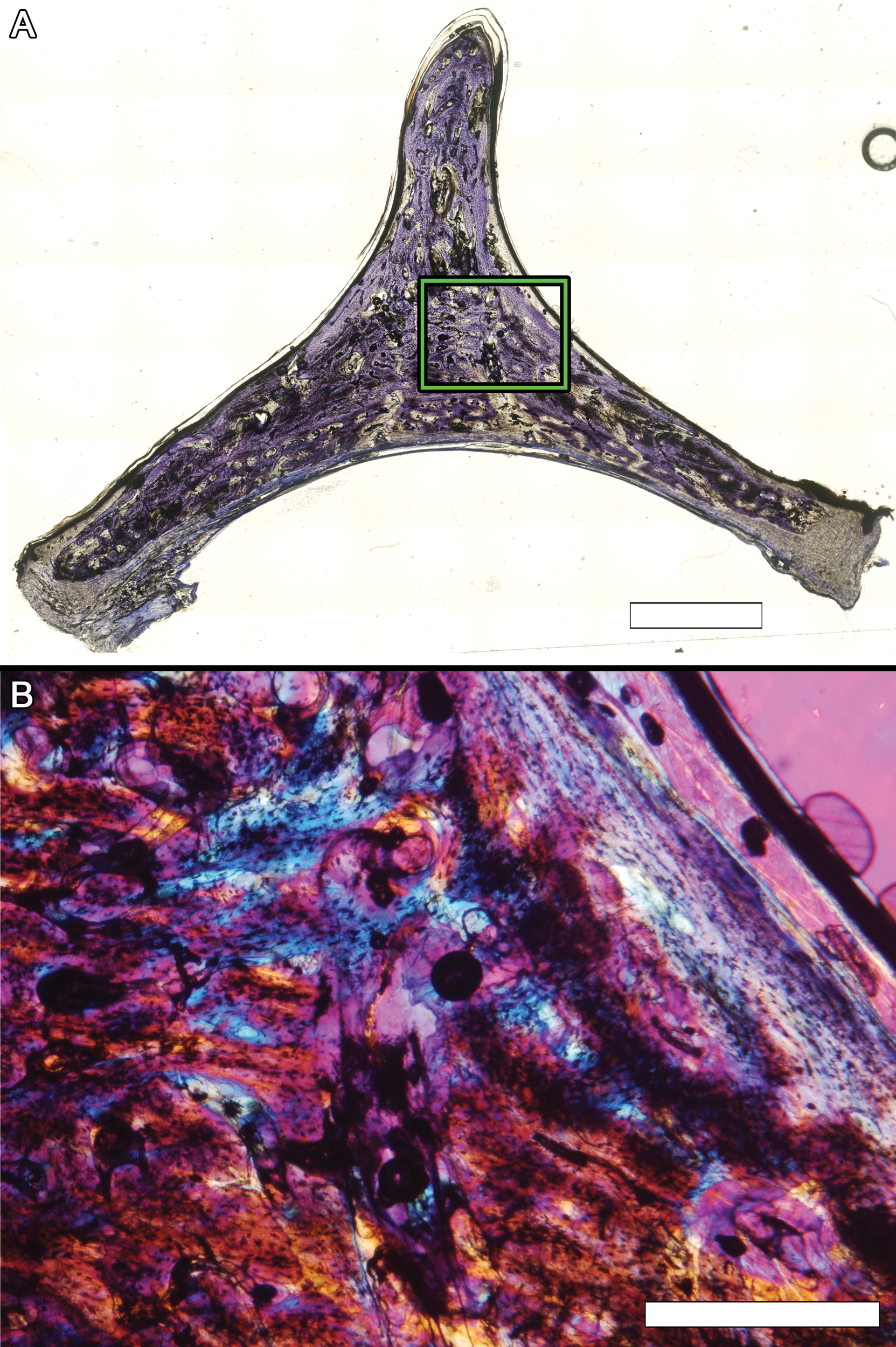

Supplement: Figure S25 — (A) Transverse section, stained with Toluidine chloride. Scale bar, 1 mm. (B) Enlargement of the area from (A) within the green box, photographed using a full lambda (530 nm) plate to reveal fiber orientation. Tissue is highly disorganized and richly vascularized. The vascular canals appear transparent and do not have a common orientation. Periosteal attachment fibers are also frequent and disorganized. Scale bar, 500 µm. [file peerj-02-422-s027.png]

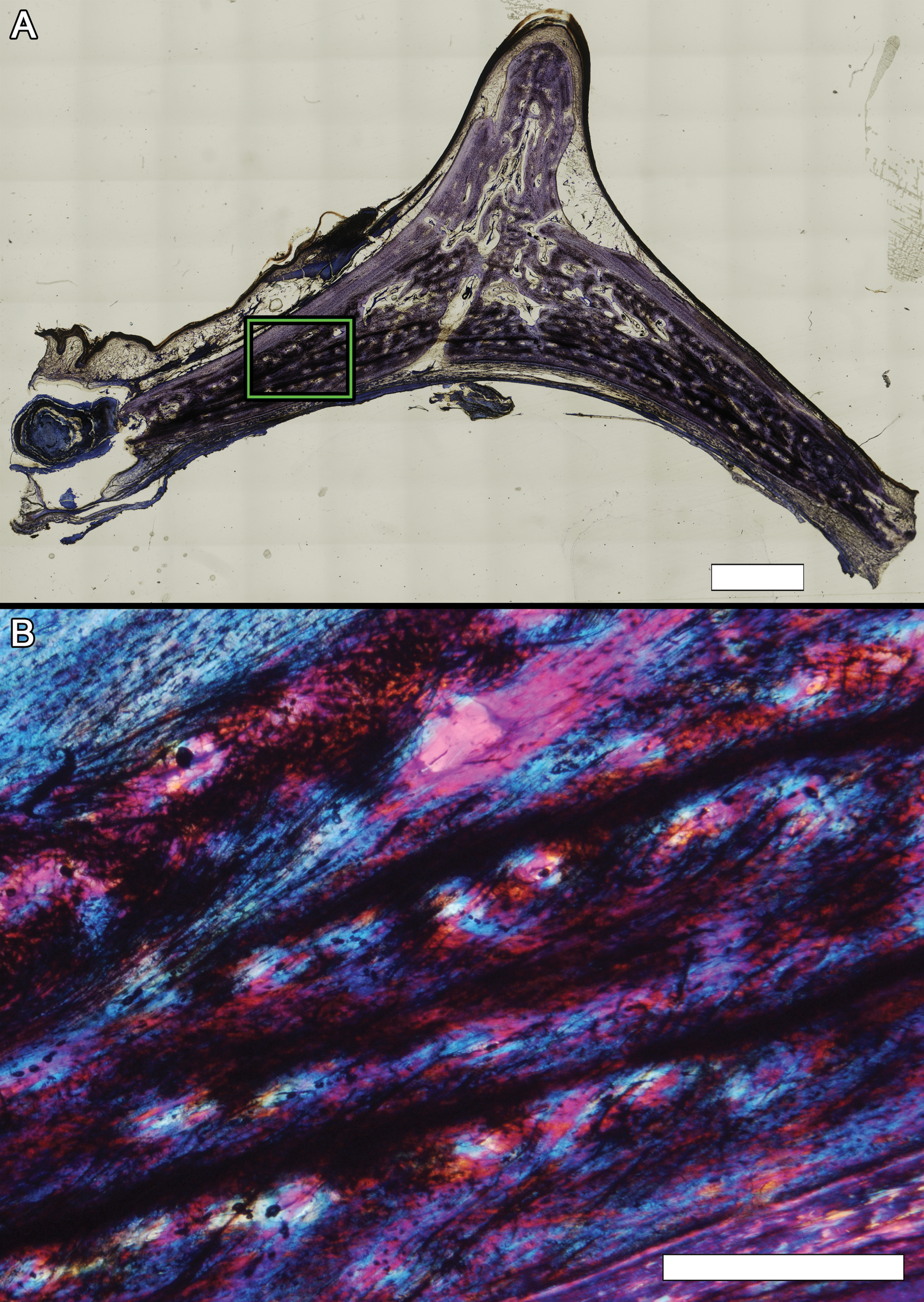

Supplement: Figure 26 — (A) Transverse section, stained with Toluidine chloride. Three to four CGMs are observed near the ventral surface at low magnification. Scale bar, 1 mm. (B) Enlargement of the area from (A) within the green box, photographed using a full lambda (530 nm) plate to reveal fiber orientation. Two dense lines of periosteal attachment fibers may indicate the location of CGMs. These fibers are also frequent and disorganized within the tissue matrix. Scale bar, 500 µm. [file peerj-02-422-s028.png]

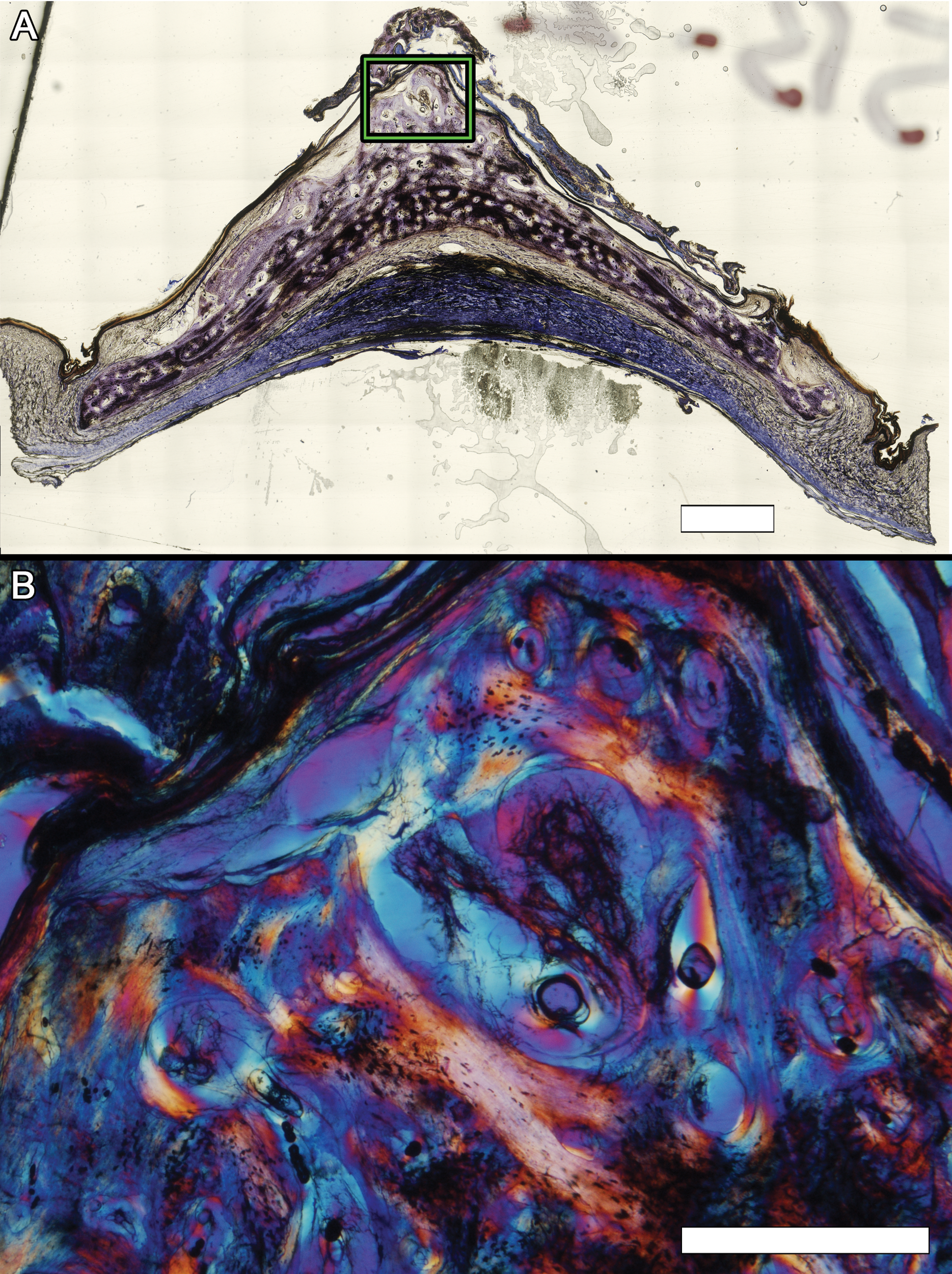

Supplement: Figure S27 — (A) Transverse section, stained with Toluidine chloride. One CGM is observed near the ventral surface at low magnification, seeming to outline the shape of the osteoderm earlier in ontogeny. Scale bar, 1 mm. (B) Enlargement of the area from (A) within the green box, photographed using a full lambda (530 nm) plate to reveal fiber orientation. The region near the apex of the keel is richly vascularized and bone is cancellous. Scale bar, 500 µm. [file peerj-02-422-s029.png]
